# Natural Variation in the *VELVET* Gene *bcvel1* Affects Virulence and Light-Dependent Differentiation in *Botrytis cinerea*

**DOI:** 10.1371/journal.pone.0047840

**Published:** 2012-10-31

**Authors:** Julia Schumacher, Jean-Marc Pradier, Adeline Simon, Stefanie Traeger, Javier Moraga, Isidro González Collado, Muriel Viaud, Bettina Tudzynski

**Affiliations:** 1 Institut für Biologie und Biotechnologie der Pflanzen (IBBP), Westfälische Wilhelms-Universität Münster, Münster, Germany; 2 INRA, BIOGER, Grignon, France; 3 Organic Chemistry Department, Cádiz University, Puerto Real, Cádiz, Spain; University of Nebraska, United States of America

## Abstract

*Botrytis cinerea* is an aggressive plant pathogen causing gray mold disease on various plant species. In this study, we identified the genetic origin for significantly differing phenotypes of the two sequenced *B. cinerea* isolates, B05.10 and T4, with regard to light-dependent differentiation, oxalic acid (OA) formation and virulence. By conducting a map-based cloning approach we identified a single nucleotide polymorphism (SNP) in an open reading frame encoding a VELVET gene (*bcvel1*). The SNP in isolate T4 results in a truncated protein that is predominantly found in the cytosol in contrast to the full-length protein of isolate B05.10 that accumulates in the nuclei. Deletion of the full-length gene in B05.10 resulted in the T4 phenotype, namely light-independent conidiation, loss of sclerotial development and oxalic acid production, and reduced virulence on several host plants. These findings indicate that the identified SNP represents a loss-of-function mutation of *bcvel1*. In accordance, the expression of the B05.10 copy in T4 rescued the wild-type/B05.10 phenotype. BcVEL1 is crucial for full virulence as deletion mutants are significantly hampered in killing and decomposing plant tissues. However, the production of the two best known secondary metabolites, the phytotoxins botcinic acid and botrydial, are not affected by the deletion of *bcvel1* indicating that other factors are responsible for reduced virulence. Genome-wide expression analyses of B05.10- and Δ*bcvel1*-infected plant material revealed a number of genes differentially expressed in the mutant: while several protease- encoding genes are under-expressed in Δ*bcvel1* compared to the wild type, the group of over-expressed genes is enriched for genes encoding sugar, amino acid and ammonium transporters and glycoside hydrolases reflecting the response of Δ*bcvel1* mutants to nutrient starvation conditions.

## Introduction

The ascomycetous fungus *Botrytis cinerea* Persoon:Fries [teleomorph *Botryotinia fuckeliana* (de Bary) Whetzel] is the causative agent of gray mold disease in more than 200, mainly dicotyledonous, plant species including grapevine and strawberry. The fungus is a typical necrotroph whose infection cycle includes the induction of plant cell death followed by the maceration of the plant tissue and reproduction by forming asexual spores on the rotted plant material. Disease symptoms depend on the host plant, the infected part of the plant and the environmental conditions. In general, *B. cinerea* is responsible for severe economic losses that are either due to the damage of growing plants in the field or the rot of harvested fruits, flowers and vegetables during storage under cold and humid conditions [1]–[3].

To date, very few virulence determinants have been identified by gene replacement approaches as most potential virulence factors are redundant in the genome [4]–[6]. For instance, an effect of phytotoxins on virulence is only visible when strains are affected in both botrydial (BOT) and botcinic acid (BOA) biosynthesis [7]–[9]. In accordance, the study by Reino et al. showed that from eleven *B. cinerea* isolates tested only the more aggressive ones were able to produce BOA in addition to BOT [10]. The endo-β-1,4-xylanase BcXYN11A, the endopolygalacturonase BcPG2 and the cerato-platanin family protein BcSPL1 are necrosis-inducing proteins representing bona-fide virulence factors as they are essential for lesion spread [11]–[14].

*B. cinerea* can reproduce asexually by forming multinucleate macroconidia on branched conidiophores for dispersal (summer cycle) or sclerotia for survival in adverse weather conditions (e.g. for over-wintering). The sclerotia can germinate either vegetatively to produce mycelia and conidia, or carpogenically to initiate the sexual cycle including the formation of apothecia that contain the ascospores [15]. *B. cinerea* is a heterothallic fungus in which sexual recombination requires partners carrying the opposite mating types (*MAT1-1* and *MAT1-2*, respectively). Sclerotia act as ‘female’ parent and microconidia (spermatia) formed by phialides arising from basal hyphae act as the ‘male’ parent. As isolates produce both sclerotia and microconidia they can usually function as the ‘female’ and ‘male’ parent in reciprocal crosses [16], [17]. The laboratory crossing of *B. cinerea* isolates can be readily induced under standardized conditions including temperature shifts and different light conditions [18]. The differentiation of reproductive structures is especially controlled by the applied light conditions: while formation of melanized conidiophores with conidia is induced by near-UV/blue light [19], sclerotia are readily formed when *B. cinerea* is incubated in continuous darkness. Small dosages of white light and blue light (60 min) are sufficient for inhibition of sclerotial development while yellow, red and far-red light promote the formation of sclerotia [19], [20]. The development of apothecia also requires light as in the close relative *Sclerotinia sclerotiorum* in which normal apothecial development is strictly dependent on near-UV light or daylight as other qualities of light result in misshaped apothecia [21].

Light strongly affects asexual and/or sexual development also in several other fungal species. A regulatory protein (VeA/VELVET) linking light signals with development and secondary metabolism was firstly identified in *Aspergillus nidulans*: Δ*veA* mutants showed light-independent conidiation (restricted to light conditions in wild type), loss of fruiting body formation (predominantly produced in darkness by the wild type) [22], and reduced production of secondary metabolites such as penicillin and sterigmatocystin [23]. Further studies revealed that VeA acts as a bridging factor in a heteromeric protein (VELVET) complex that furthermore includes two other members of the VELVET protein family (VelB and VosA), the putative histone methyltransferase LaeA, the red light-sensing phytochrome FphA, and the blue light-responding GATA transcription factors LreA/LreB [24]. Functional characterization of VeA homologues in *Aspergillus parasiticus* [25], *Aspergillus fumigatus* [26], *Fusarium verticillioides* [27], [28], *Aspergillus flavus* [29], *Acremonium chrysogenum* [30], *Neurospora crassa* [31], *Fusarium fujikuroi* [32], *Penicillium chrysogenum* [33], *Trichoderma virens* [34], *Mycosphaerella graminicola* [35], *Fusarium graminearum* [36], [37], *Dothistroma septosporum* [38], *Cochliobolus heterostrophus* [39], and *Histoplasma capsulatum* [40], confirmed the universal role of VELVET as a global regulator of development and secondary metabolism in ascomycetes.

*B. cinerea* field populations are known for high genetic variation regarding their aggressiveness on different plant species, their spectra of produced phytotoxins, their resistance to fungicides, and their preferred mode of reproduction [10], [41]–[46]. Thus, field populations represent natural collections of genotypes and phenotypes that arise by random mutations resulting in single-nucleotide polymorphisms (SNP) or even in chromosome rearrangements. The aim of this study was the elucidation of the genetic basis for phenotypic differences with regard to virulence, oxalic acid (OA) production and light-dependent differentiation between the two sequenced *B. cinerea* isolates: the aggressive and sclerotia-forming isolate B05.10 and the less aggressive, non-sclerotia-forming isolate T4 [47]. We pursued a map-based cloning approach using the progeny of a cross between strain T4 and a strain that was phenotypically identical with B05.10. The analysis of the progeny provided evidence that these three markers (virulence, OA production, sclerotia formation) are genetically linked and revealed the *VELVET* gene *bcvel1* as candidate for the gene locus responsible for the observed phenotypic differences. Deletion and complementation analyses confirmed the role of BcVEL1 in light-dependent differentiation, OA formation and virulence.

## Results

### Identification of *bcvel1* as a candidate for the sclerotia gene locus

*B. cinerea* strain B05.10 is used for molecular studies in several laboratories and exhibits light-dependent development similar to that of other natural isolates. In contrast, strain T4 conidiates in light and darkness and does not produce any sclerotia. This strain is also reduced in virulence and OA production when compared to B05.10, and produces only one of the two phytotoxins [7], [8]. Nevertheless, strain T4 is still able to form microconidia and to undergo sexual recombination. A cross between T4 (*MAT1-2*) and the sclerotia-forming strain 32 (*MAT1-1*) recently provided the first genetic map of *B. cinerea* [47].

**Figure 1:**
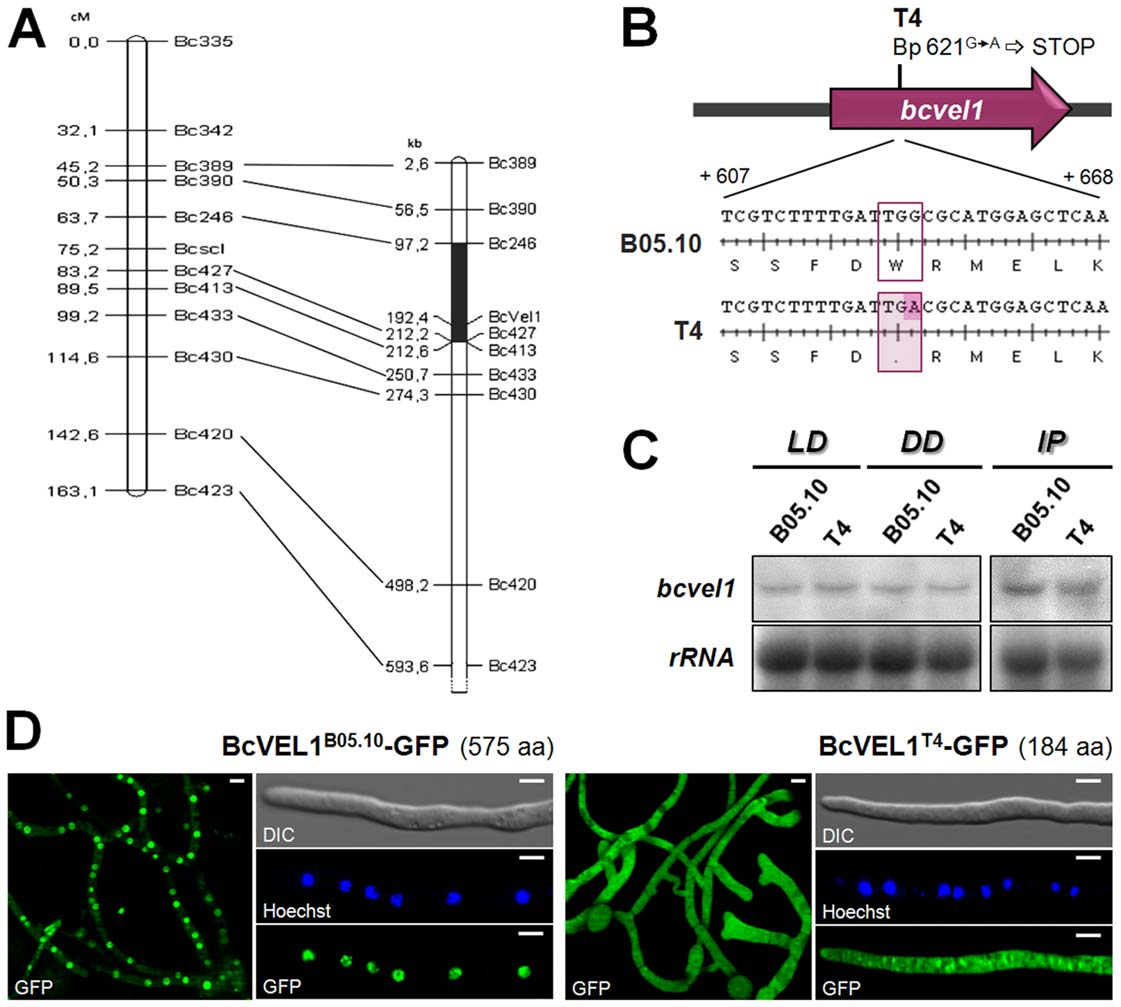
Identification of the VELVET gene *bcvel1* as candidate for the sclerotia gene locus. (A) Genetic and physical maps of the locus linked to sclerotia formation (Bcscl). The genetic map (on the left; units in centiMorgans, cM) was obtained by analyzing the progeny from a cross between strains T4 and 32. The ability to form sclerotia in 68 ascospore isolates was tested by three independent experiments. The segregation data indicated that the *Bcscl* marker is genetically linked with the microsatellite markers (Bc335, Bc342, Bc389, Bc246) of the genetic group 15. The design of additional markers (Bc427, Bc413, Bc433, Bc420 and Bc423) allowed locating *Bcscl* between markers Bc246 and Bc427 (for details see Materials and Methods). This 19.5 cM region corresponds to 115 kb of the supercontig 2.1 of the T4 genome sequence that comprises *bcvel1* (shaded in black on the right; see also Figure S1). (B) Comparison of *bcvel1* sequences derived from wild-type isolates B05.10 and T4. A SNP (base pair 621 of the coding region) in T4 results in a stop codon. (C) Expression of *bcvel1* in B05.10 and T4. Strains were grown for 3 days on solid complete medium covered with a cellophane overlay in light-dark (LD) conditions or in continuous darkness (DD). Infected plant material (48 hpi on primary leaves of *P. vulgaris*) was used to detect the transcript levels *in planta* (IP). rRNA is shown as loading control. (D) Subcellular localization of GFP-tagged BcVEL1 proteins derived from B05.10 and T4 sequences in a B05.10 genomic background. Conidia of the strains were incubated in Gamborgs B5+2% glucose on microscope slides in light-dark (LD) conditions for 18 h (12 h darkness+6 h light). Nuclei were visualized using the fluorescent dye Hoechst 33342. Scale bars represent 5 µm.

In this study, we tested sclerotia forming ability among the 68 progenies of this cross. Interestingly, about half of them (29) were able to form sclerotia suggesting that only one locus called *Bcscl* was responsible for the phenotype. Virulence tests on bean leaves furthermore revealed that the progenies that were able to form sclerotia produced symptoms similar to those of the highly virulent parental strain 32, while the progenies that were unable to form sclerotia produced smaller lesions similar to the ones provoked by strain T4. In addition, only the sclerotia-producing progenies were shown to accumulate OA similar to the parental strain 32 (data not shown). These results indicate that the *Bcscl* locus is also relevant for full virulence and OA production. To identify the respective locus, a map-based cloning approach was performed. By identifying new polymorphic microsatellite markers (details in Materials and Methods) that co-segregate with the *Bcscl* marker in the progeny, it was possible to identify a 115-kb genomic region on supercontig 2.1 of the T4 genome sequence (Fig. 1A). In this region, 44 (BofuT4_P003110 to BofuT4_P003540) and 41 genes (BC1G_02969 to BC1G_03009) were automatically predicted in the T4 and B05.10 genomes, respectively (Fig. S1). Among them, we found two already functionally characterized genes encoding the stress-activated MAP kinase BcSAK1 and the Gα subunit BCG3 [48], [49]. A single nucleotide polymorphism (SNP) was identified in a gene that corresponds to BofuT4_P003460/BC1G_02976 encoding a protein with a *VELVET* superfamily domain (details in Materials and Methods). Re-sequencing of the gene locus termed *bcvel1* (*B. cinerea VELVET 1*) in the different isolates (T4, B05.10 and 32) confirmed the G to A mutation of base pair 621 in isolate T4 (Fig. 1B) and furthermore showed that the published B05.10 sequence contains a sequencing error resulting in two incorrectly annotated proteins (BC1G_02976 and BC1G_02977). The SNP in T4 leads to an early stop codon and consequently to a truncated protein with only 184 aa (BcVEL1^T4^) instead of 575 aa (BcVEL1^B05.10^). As shown in Fig. 1C, *bcvel1* is comparably expressed in both isolates *in vitro* in different light regimes and during infection of *Phaseolus vulgaris* indicating that the SNP does not affect *bcvel1* transcript levels. To see whether the mutation results in different subcellular localization patterns, *gfp* fusion constructs for both *bcvel1* copies were constitutively expressed in the B05.10 genomic background. While the BcVEL1^B05.10^-GFP fusion protein was localized in the nuclei of growing hyphae, the truncated BcVEL1^T4^ protein was predominantly found in the cytosol (Fig. 1D).

### BcVEL1 is the homologue of *A. nidulans* VeA and *F. fujikuroi* VEL1

**Figure 2:**
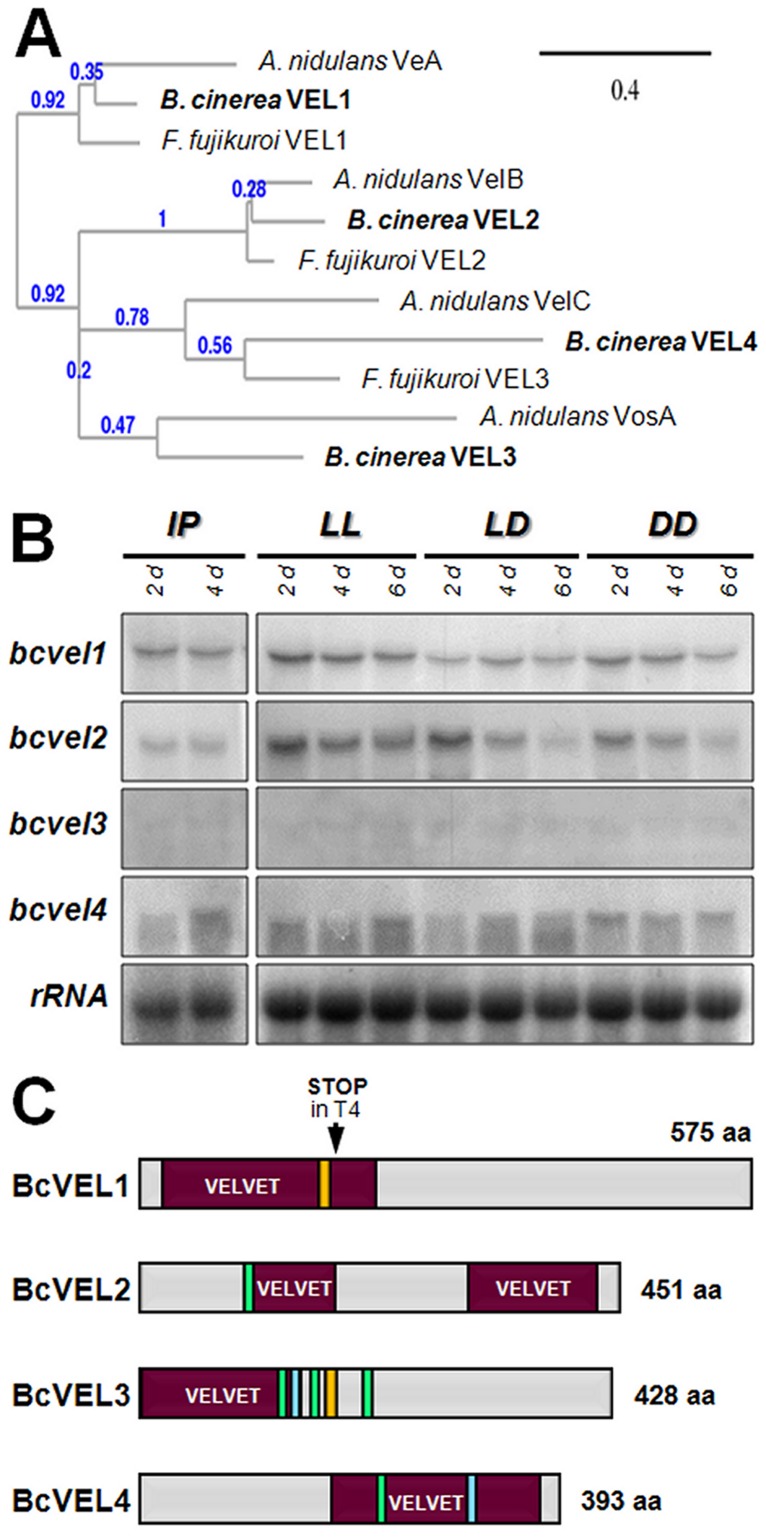
The four VELVET proteins of *B. cinerea*. (A) Phylogenetic tree of *VELVET* proteins of *B. cinerea*, *A. nidulans* and *F. fujikuroi*. Sequence alignment and tree construction were performed using the bioinformatics program at Phylogeny.fr (http://www.phylogeny.fr/). Analysed sequences are: *B. cinerea* BcVEL1^B05.10^ (HE977589), BcVEL2^B05.10^ (HE977591), BcVEL3^B05.10^ (HE977592), BcVEL4^B05.10^ (HE977594), *A. nidulans* VeA (AAD42946), VelB (ABQ17967), VelC (ABQ17968), VosA (ABI51618), *F. fujikuroi* VEL1 (CBE54373), VEL2 (CBK25977), and VEL3 (L. Studt and B. Tudzynski, unpublished data). The sequence alignment of BcVEL1 with other homologous protein sequences is shown in Fig. S2A. (B) Expression of the four *VELVET*-like genes during different stages of the life cycle of *B. cinerea*. Wild-type strain B05.10 was incubated on complete medium covered with cellophane overlays for the indicated time periods in continuous light (LL) and light-dark (LD) for induction of conidiation and in continuous darkness (DD) for induction of sclerotia formation (non-pigmented sclerotia initials were present after 6 d of incubation). Infected plant material (primary leaves of *P. vulgaris*, harvested 2 dpi and 4 dpi) was used to detect the transcripts *in planta* (IP). rRNA is shown as loading control. (C) Domain architecture of the four identified VELVET-like proteins of *B. cinerea*. Putative nuclear localization signals (NLS) are shown as green bars, leucine-rich nuclear export signals (NES) as blue bars, and potential PEST domains (proline (P), glutamate (E), serine (S) and threonine (T)-rich) are indicated as orange bars.

As fungal genomes are known to comprise several VELVET proteins, we identified the other VELVET-like proteins in *B. cinerea* by BlastP analyses (hereinafter referred to BcVEL2 to BcVEL4). In the phylogenetic analysis of the known VELVET-like proteins from *B. cinerea*, *A. nidulans* and *F. fujikuroi*, BcVEL1 groups together with VeA (54%) [22] and FfVEL1 (57%) [32], BcVEL2 with VelB (36%) [50] and FfVEL2 (53%) [32], BcVEL3 with VosA (44%) [50] and BcVEL4 with VelC (28%) [50], and FfVEL3 (38%) [L. Studt and B. Tudzynski, unpublished data] (Fig. 2A). Interestingly, *F. fujikuroi* lacks the fourth VELVET-like protein that is found in both *A. nidulans* and *B. cinerea*. SNPs were also found in the other *VELVET* genes of B05.10 and T4; however, all these mutations are silent. Only one indel was found. In *bcvel3^T4^*, one codon (base pairs 695 to 698 of the B05.10 sequence) is missing, and consequently the protein lacks one amino acid (for details see Materials and Methods).

Northern blot analyses showed that all four *VELVET* genes are constitutively expressed throughout the life cycle; during light-induced conidiation, sclerotial development in darkness and during infection of *P. vulgaris* (Fig. 2B). In addition to the highly conserved VELVET domains, putative nuclear localization signals (NLS), nuclear export signals (NES) and PEST domains that act as signals for protein degradation were predicted and indicated in Fig. 2C. Although BcVEL1 is found in the nucleus (Fig. 1D) neither NLS nor NES could be identified in its sequence using bioinformatics tools. The sequence alignment of BcVEL1 with other VeA-homologous proteins from *S. sclerotiorum* (Broad Institute Database), *N. crassa* [31], *A. parasiticus* [25] and *H. capsulatum* [40] demonstrates the high conservation of the N-terminal *VELVET* domain (aa 20 to aa 220) in contrast to the low conservation of the C terminus (Fig. S2). The truncated protein from *B. cinerea* T4 still contains the largest part of the *VELVET* domain while the variable region which is likely required for the accumulation in the nuclei is missing (Fig. 2C, Fig. S2).

To test whether BcVEL1 is indeed a functional homologue of VeA-like proteins, we pursued a cross-genus complementation strategy using the *F. fujikuroi* Δ*Ffvel1* mutant [32] as a recipient for the introduction of the *B. cinerea* gene. *F. fujikuroi* predominately occurs on rice where it causes the bakanae disease, resulting in a variety of symptoms such as abnormal elongation of plants and yellowish leaves that are due to fungal gibberellin (GA) production. The deletion of FfVEL1 was shown to affect differentiation processes (loss of microconidia formation, elevated perithecia formation), secondary metabolism (loss of GA production, increased bikaverin production) as well as virulence on rice seedlings [32]. To determine if these phenotypes could be rescued by BcVEL1, we expressed *bcvel1^B05.10^* under control of its own promoter in the *Ffvel1* deletion mutant. Transformants were obtained using nourseothricin as the selection agent, and integration of the *bcvel1* construct was confirmed by PCR yielding strains Δ*Ffvel1*+*bcvel1*-3, -4, and -6. As shown in Fig. S3, the expression of BcVEL1 in the Δ*Ffvel1* background almost fully restored the ability to produce aerial hyphae and microconidia, and to reduce the formation of the red pigment bikaverin to wild-type levels (Fig. S3A–C). However, *bcvel1* expression did not restore the production of GAs and the elongation phenotype on rice seedlings (Fig. S3D–E).

### BcVEL1 regulates differentiation processes and the formation of OA and melanin

To investigate the role of VEL1 in *B. cinerea* in more detail and to determine the relevance of the identified mutation in the corresponding gene for the phenotype of isolate T4, *bcvel1* was deleted in the more virulent and sclerotia-forming isolate B05.10. In addition, both *bcvel1* copies were expressed in several genomic backgrounds.

Transformation of B05.10 with the *bcvel1* replacement construct (Fig. S4A) produced three knock-out mutants (Δ*bcvel1*-T5, -T8, and -T22). Diagnostic PCR and Southern blot analyses of three transformants demonstrated the replacement of *bcvel1*, the absence of further ectopic integrations of the construct and finally the absence of any wild-type alleles (Fig. S4B–C). As all mutants exhibited the same phenotype, the results for one arbitrarily chosen deletion mutant (Δ*bcvel1*-T5) are shown. A complementation construct was generated comprising the open reading frame of *bcvel1^B05.10^* and 5′- and 3′-non-coding regions of *bcvel1* for targeted integration at the native gene locus and a nourseothricin resistance cassette (Fig. S4A). The construct was transformed into the deletion mutant (Δ*bcvel1*-T5) and into strain T4 resulting in the replacement of the hygromycin resistance cassette in Δ*bcvel1* and the mutated *bcvel1* copy in the T4 background. Furthermore, the *bcvel1^B05.10^-gfp* and *bcvel1^T4^-gfp* fusion constructs were expressed in the Δ*bcvel1*-T5 background to investigate their capacity to restore the wild-type phenotype. All generated mutants and their genotypes are listed in Table 1. Expression of *bcvel1* in the different mutants was verified by northern blot analyses (Fig. S4E).

**Table 1:** Strains used in this study.

Fungus/strain	Description/genotype	Reference
***Botrytis cinerea***		
T4	Isolate from tomato (France); *MAT1-2*	[47]
32	Isolate from *Vitis* (France); *MAT1-1*	[47]
SAS405	Isolate from *Vitis* (Italy); *MAT1-2*	[16]
B05.10	Isolate from *Vitis* (Germany); *MAT1-1*	[78]
Δ*bcvel1*	B05.10, Δ*bcvel1::hph,* homokaryon	This study
Δ*bcvel1*+*bcvel1*	B05.10, Δ*bcvel1::hph*, *bcvel1::nat1*, heterokaryon	This study
T4+*bcvel1*	T4, *bcvel1*::*nat1*, heterokaryon	This study
B05.10+*bcvel1-gfp*	B05.10, OE*bcvel1^B05.10^-gfp::nat1*, heterokaryon	This study
Δ*bcvel1*+*bcvel1-gfp*	B05.10, Δ*bcvel1::hph*, OE*bcvel1^B05.10^-gfp::nat1*, heterokaryon	This study
B05.10+*bcvel1^T4^-gfp*	B05.10, Δ*bcvel1::hph*, OE*bcvel1^T4^-gfp::nat1*, heterokaryon	This study
Δ*bcvel1*+*bcvel1^T4^-gfp*	B05.10, Δ*bcvel1::hph*, OE*bcvel1^T4^-gfp::nat1*, heterokaryon	This study
***Fusarium fujikuroi***		
IMI58289	Wild-type strain (Commonwealth Mycological Institute, UK)	
Δ*Ffvel1*	IMI58289, Δ*ffvel1::hph*	[32]
Δ*Ffvel1*+*bcvel1*	IMI58289, Δ*ffvel1::hph*, *bcvel1::nat1*	This study

**Figure 3:**
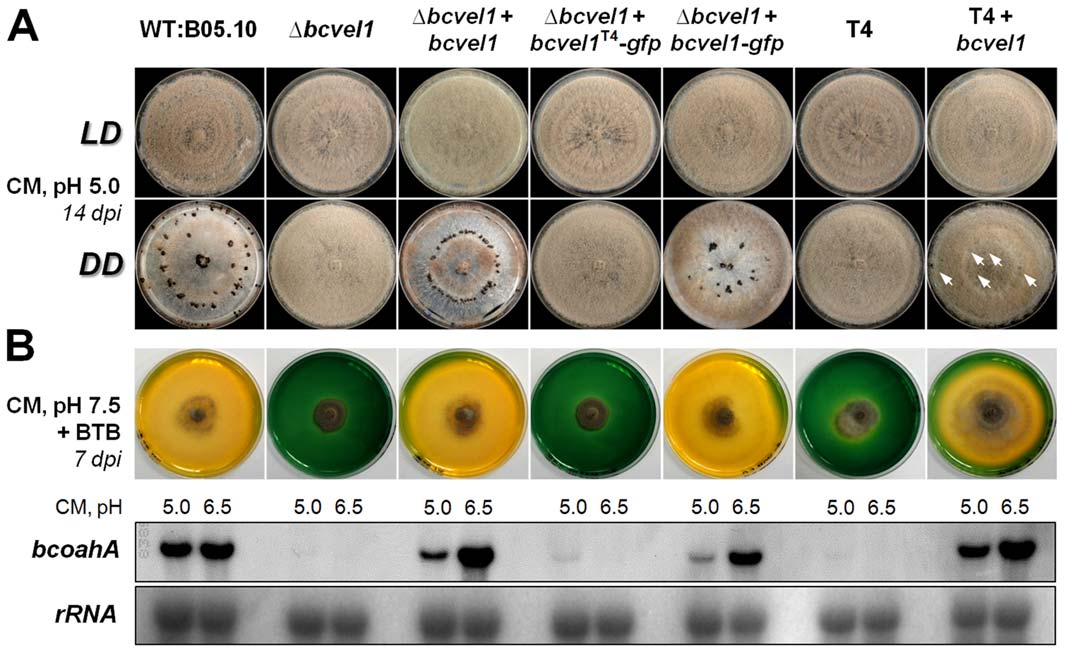
Effects of the different *bcvel1* mutations on light-dependent differentiation and oxalic acid (OA) formation. (A) Conidiation and sclerotia formation. The strains were incubated on solid complete medium (CM) at 20°C in light-dark (LD) conditions for conidiation and in continuous darkness (DD) for sclerotia formation. The few sclerotia formed by the mutant T4+*bcvel1* are indicated by white arrows. (B) OA secretion. Acidification of the culture medium was monitored on solid CM pH 7.5 supplemented with 0.1% bromothymolblue (BTB, pH indicator). The color change from green to yellow indicates acidification (pH<6.0). The expression of *bcoahA* encoding the OA-forming enzyme oxaloacetate hydrolase was detected by northern blot analysis. For that, strains were grown for three days on solid CM with pH 5.0 or pH 6.5 covered with cellophane overlays. rRNA is shown as loading control.

The generated strains were screened for the phenotypes previously found to be linked with the *bcvel1* locus. Like strain T4, the B05.10:*bcvel1* deletion mutants and the deletion mutant expressing the truncated gene copy (Δ*bcvel1*+*bcvel1^T4^-gfp*) formed conidia instead of sclerotia in continuous darkness and failed to acidify the culture medium in a B05.10-like manner (Fig. 3). In contrast, strains expressing the *bcvel1^B05.10^* copy in different genomic backgrounds (Δ*bcvel1*+*bcvel1^B05.10^*; Δ*bcvel1*+*bcvel1^B05.10^-gfp*; T4+*bcvel1^B05.10^*) acidified the culture medium and formed sclerotia, though only a few sclerotia were observed for the T4+*bcvel1^B05.10^* mutant. Analysis of expression levels of *bcoahA* encoding the oxalic-acid-forming enzyme oxaloacetate hydrolase [51] supports the hypothesis that the non-acidifying phenotype of *bcvel1*-loss-of-function mutants is due to decreased OA production (Fig. 3B).

**Figure 4:**
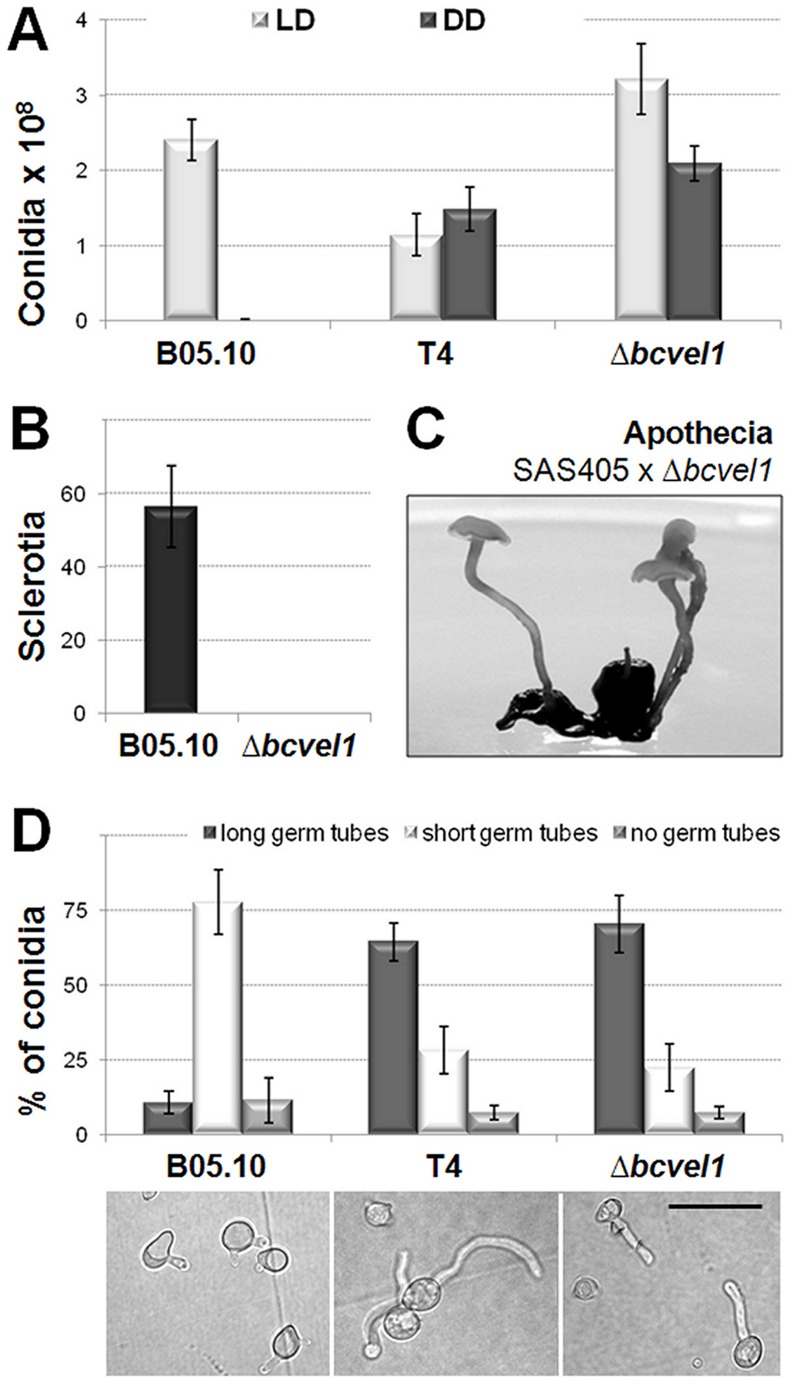
Impact of BcVEL1 on asexual and sexual reproduction and conidial germination. (A) Numbers of conidia (per Petri dish) produced by the strains in light-dark (LD) conditions and in continuous darkness (DD), respectively. Data represent mean values of the results from three Petri dishes per strain and condition. (B) Numbers of sclerotia (per Petri dish) produced by the strains in continuous darkness. Data represent mean values of the results from three Petri dishes per strain and condition. (C) Apothecia derived from crossing of sclerotia of strain SAS405 (*MAT1-2*) with microconidia of mutant B05.10:Δ*bcvel1* (*MAT1-1*). Picture was taken after 14 weeks of joining sclerotia and microconidia. (D) Germination on hydrophobic surfaces. Conidia were suspended in water and incubated for 24 h on polypropylene. The experiments were done in triplicates, each time hundred conidia were counted. Short germ tubes do not exceed the length of the conidia while long germ tubes do. Scale bar represents 20 µm.

Beside the light-independent conidiation of *bcvel1* loss-of-function mutants, we also observed differences with regard to the conidia quantity and the onset of conidiation. Thus, the formation of conidia started earlier and the numbers of conidia formed are significantly higher even under light conditions (Fig. 4A, S5A). In addition, different conidiation patterns were observed in response to 12 h light/12 h dark rhythm: while the wild type B05.10 formed regular growth rings on solid CM medium (a gray ring due to conidiation in the light is followed by a white ring due to the absence of conidiation in the dark), the Δ*bcvel1* mutant showed a radial conidiation pattern (Fig. S6). Due to the loss of sclerotia formation (Fig. 3A, 4B), Δ*bcvel1* mutants cannot act as female parents in crossing experiments. However, Δ*bcvel1* mutants formed microconidia (*MAT1-1*) and were able to undergo sexual recombination with sclerotia of strain SAS405 (*MAT1-2*) yielding normally shaped apothecia (Fig. 4C). In addition, ascospores were able to germinate and the progeny showed the expected segregation (50%) of the mutant phenotype (data not shown).

To examine whether BcVEL1 affects conidial germination, conidia were incubated on hydrophilic surfaces in the presence of nutrients (Gamborg's B5+10 mM glucose) and on polypropylene foil without nutrients, respectively. In general, the quantification of the germination rates of B05.10, T4 and Δ*bcvel1* conidia led to similar results for both conditions. However, while all conidia showed similar germ tube morphologies in the presence of nutrients characterized by the development of thick and fast growing germ tubes (data not shown), differences between the strains were observed when conidia were incubated on hydrophobic surfaces. In contrast to conidia of the wild type B05.10, conidia of T4 and Δ*bcvel1* primarily formed elongated germ tubes (Fig. 4D), indicating defects in surface sensing.

Both the wild type B05.10 and *bcvel1* deletion mutants showed comparable growth rates on synthetic solid media independent of the applied light conditions, and also their responses to osmotic stress induced by adding 1.4 M sorbitol or 0.7 M sodium chloride, and oxidative stress induced by adding 500 µM menadione, respectively, were comparable. However, growth rates of the Δ*bcvel1* mutant were significantly affected when the strains were grown on medium supplemented with 7.5 M hydrogen peroxide in continuous light (Fig. S5B). DAB (3,3′-diaminobenzidine) staining did not reveal differences between strains with regard to the accumulation of reactive oxygen species (ROS) during growth in different light regimes (Fig. S5C).

Furthermore, we observed that the *bcvel1* deletion mutants secreted a dark pigment. To get an indication whether the dark pigment was dihydroxynaphthalene (DHN)-melanin, the media were supplemented with tricyclazole representing a specific reductase inhibitor that results in the accumulation of the orange pigment flaviolin, a shunt product of the first detectable intermediate in the DHN-melanin biosynthetic pathway [52]. As shown in Fig. S7A, culture broths of Δ*bcvel1* mutants showed a dark coloration without the inhibitor and an orange coloration when the broth was supplemented with tricyclazole indicating that the dark pigmentation is due to the accumulation of DHN-melanin. In both strains, pigment formation was induced by light, but the effect was much more pronounced in Δ*bcvel1* mutants. In accordance with the dark pigmentation of the culture broth, the analyzed melanin biosynthetic genes (*bcpks13*, *bcbrn1*, and *bcscd1* encoding the polyketide synthase, the 1,3,8-trihydroxynaphthalene (THN) reductase, and the scytalone dehydratase, respectively [53]) were significantly over-expressed in Δ*bcvel1* mutants (Fig. S7B).

In summary, BcVEL1 is a regulator of light-associated differentiation processes such as the formation of macroconidia and sclerotia, and the production of the dark pigment melanin. Furthermore, BcVEL1 acts a positive regulator of OA secretion that is required for growth on media with alkaline pH values. However, under acidic growth conditions the overall fitness of *bcvel1* loss-of-function is not affected, and the formation of microconidia still allows for one-way crossings.

### BcVEL1 is essential for full virulence on different hosts

**Figure 5:**
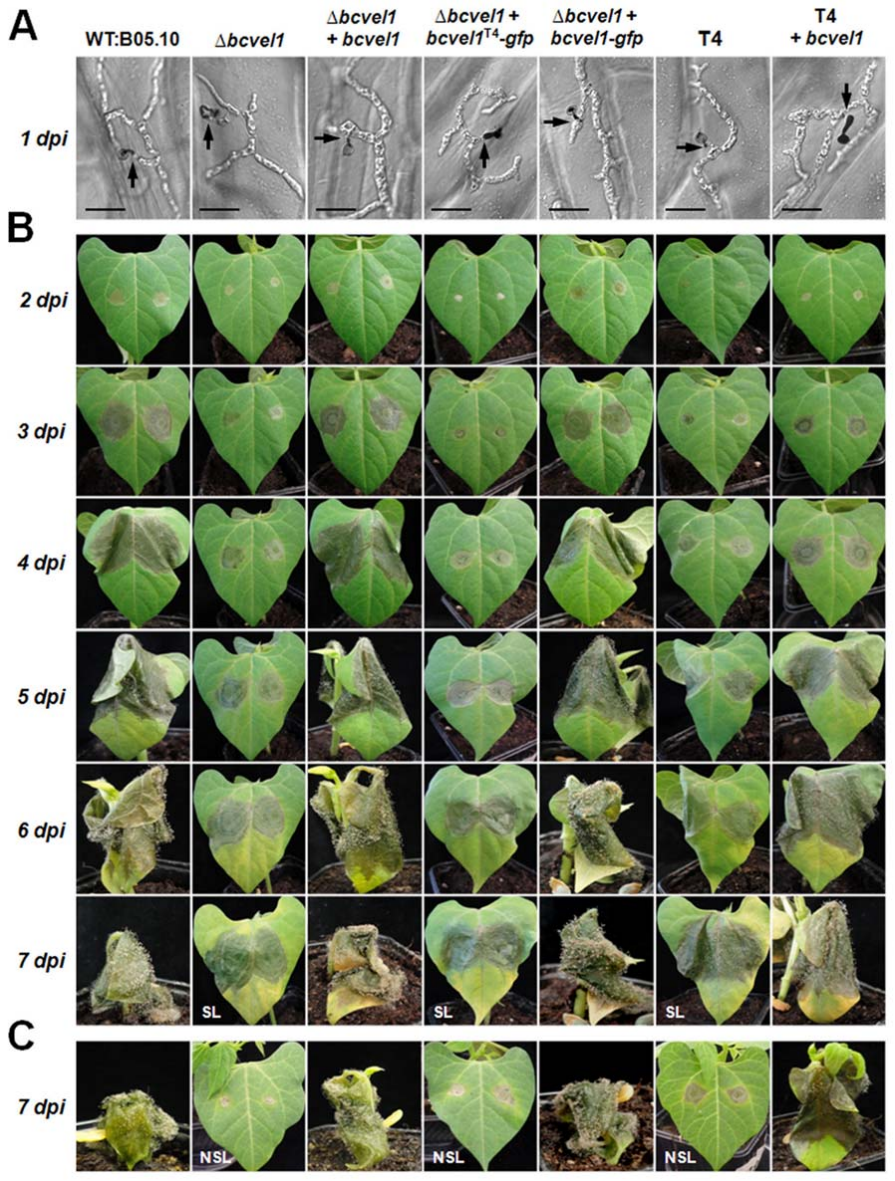
Virulence of strains carrying the different *bcvel1* mutations. (A) Penetration of plant cells by germ tubes on onion epidermal strips (1 dpi). Conidia and hyphae on the surface were stained with lactophenol blue. Sites of penetration are indicated by black arrows. Scale bars represent 20 µm. (B) Complete infection cycle on primary leaves of *P. vulgaris* (2 to 7 dpi). Plants were incubated in humid conditions under natural illumination at room temperature. In this experiment, lesions of *bcvel1* loss-of-function mutants were able to spread (SL, spreading lesions). (C) A further virulence assay on primary leaves of *P. vulgaris* (only 7 dpi). In this experiment, infection by *bcvel1* loss-of-function mutants stopped in the primary stage (NSL, non-spreading lesions), while infection by the wild type B05.10 and complemented mutants was not affected.

**Figure 6:**
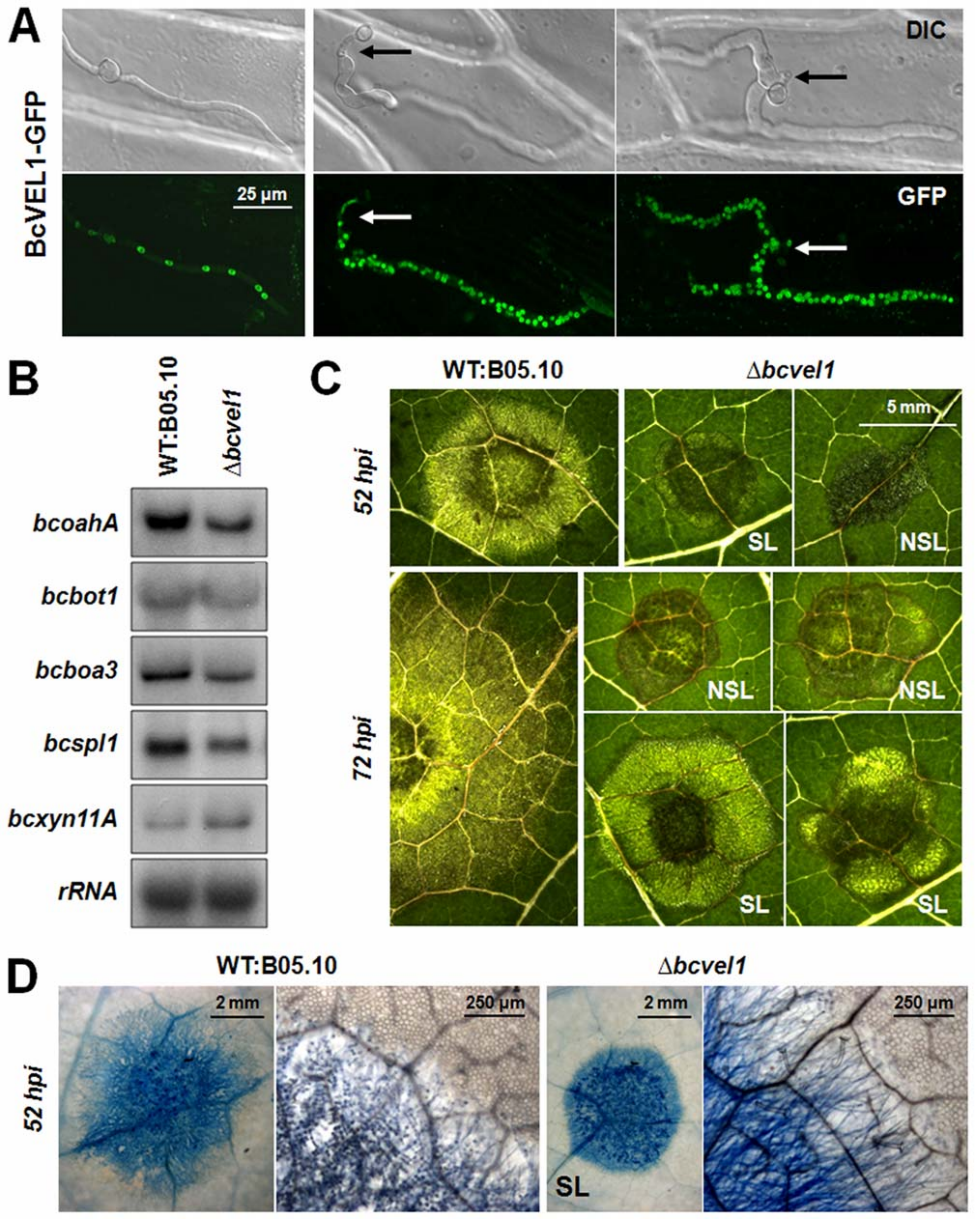
Impact of BcVEL1 on invasive growth. (A) Subcellular localization of BcVEL1-GFP (in the B05.10 genomic background) during infection of onion epidermal layers (24 hpi). The localization is shown in hyphae that are growing on the top of the cells (left panel) or growing invasively (middle and right panel); arrows indicate sites of penetration. (B) Expression of known virulence factors in B05.10 and Δ*bcvel1 in planta*. Primary leaves of *P. vulgaris* were inoculated with conidial suspensions and harvested 48 hpi (primary lesions, prior to lesion spreading). Northern blots were hybridized with probes of *bcoahA* (oxalic acid biosynthesis), *bcbot1* (botrydial biosynthesis), *bcboa3* (botcinic acid biosynthesis), *bcspl1* (cerato-platanin family protein) and *bcxyn11A* (endo-beta-1,4-xylanase) coding sequences. rRNA is shown as loading control. (C) Close-up views of lesions caused by B05.10 and Δ*bcvel1* on *P. vulgaris*. Outcome of the Δ*bcvel1*-plant interaction is variable: infection stops in primary stage (NSL, non-spreading lesion) or proceeds slowly (SL, spreading lesion). (D) Trypan blue staining of spreading lesions (52 hpi). Fungal hyphae and dead plants cells are stained blue. The right panels show higher magnification of the edges of the lesions. Only few dead plant cells were observed around Δ*bcvel1* lesions, while massive staining of plant cells in the range of the wild-type lesions indicates the complete maceration of the tissue.

The most interesting characteristic of the T4 phenotype is the reduced virulence on several host plants e.g. French bean and tomato. No differences between the analyzed strains, including the deletion and complementation mutants, were observed for their capabilities to enter onion epidermal cells: all conidia formed short germ tubes that directly penetrated the plant cells (Fig. 5A). The BcVEL1-GFP fusion protein (expressed in the wild-type background) localized to the nuclei in hyphae that grew on the surface of the onion epidermis as well as in hyphae that grew inside the plant cells. Interestingly, both types of hyphae differed in their numbers of nuclei (Fig. 6A). The invasively growing hyphae were filled with nuclei, while the thinner hyphae growing on the surface of the epidermis contained fewer nuclei in fixed intervals as it is observed for hyphae growing on microscope slides (Fig. 1D).

While *bcvel1* is dispensable for the early stages of infection (penetration), it plays a crucial role during the advanced stages i.e. during colonization of primary leaves of living *P. vulgaris* plants. Similar to strain T4, *bcvel1* deletion mutants were significantly impaired in invasive growth and their capacities to form conidia on the infected plant tissue. The fact that the expression of *bcvel1^B05.10^* in the T4 background (strain T4+*bcvel1^B05.10^*) elevated virulence almost to B05.10 levels and that on the other hand the expression of *bcvel1^T4^* in the Δ*bcvel1* background (Δ*bcvel1*+*bcvel1^T4^-gfp*) failed to rescue the virulence phenotype indicate that the reduced virulence of isolate T4 is due to the mutated *bcvel1* copy (Fig. 5). However, the outcome of the infection by the Δ*bcvel1* mutants was found to vary from experiment to experiment, possibly due to minor differences in the green house conditions in which the bean plants were grown. In some experiments *bcvel1* loss-of-function mutants were able to produce spreading lesions (SL) (Fig. 5B) while in other experiments infection stopped in the primary stage (NSL, non-spreading lesions) (Fig. 5C). When agar plugs containing non-sporulating mycelia of the strains were used for inoculation, the Δ*bcvel1* mutants predominantly developed spreading lesions (Fig. S8). Close-up views of the different types of lesions demonstrate that both spreading and non-spreading lesions of Δ*bcvel1* mutants were surrounded by defined brownish rings. Furthermore, the plant tissue within these lesions remained green while the wild type-infected area became already transparent due to the extensive maceration of the plant tissue (Fig. 6C). Trypan blue staining that allows detection of dead plant cells as well as invasively growing hyphae, revealed significant differences between spreading lesions provoked by the wild type and the Δ*bcvel1* mutant (Fig. 6D). In the area of wild-type lesions, plant cells were dead, and individual fungal hyphae were observed invading the surrounding tissue of living (colorless) cells. In contrast, staining of Δ*bcvel1* lesions illustrated the massive accumulation of fungal biomass within the restricted areas and the absence of dead plant cells in the center of the lesions. Only a few dead cells were found around the colonized areas illustrating the inability of the Δ*bcvel1* mutant to efficiently kill and macerate the host cells (Fig. 6D).

**Figure 7:**
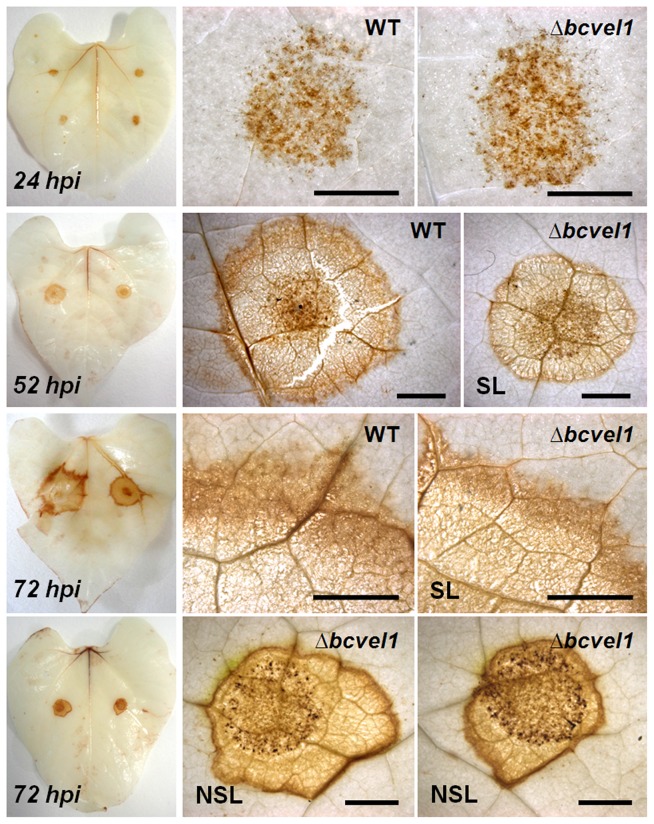
Temporal H2O2 accumulation in spreading (SL) and non-spreading (NSL) lesions. WT:B05.10- and Δ*bcvel1*-infected *P. vulgaris* leaves were stained with DAB (3,3′-diaminobenzidine) that is oxidized by hydrogen peroxide in the presence of peroxidases to give a dark-brown color. Non-spreading lesions of Δ*bcvel1* are surrounded by distinct brown rings (lower panels, pictures correspond to non-stained lesions in Fig. 6C). Scale bars represent 2 mm.

The oxidative burst is characterized by the accumulation of ROS in the extracellular space of plant tissues and is a response to abiotic stress conditions or pathogen attacks. To monitor the accumulation of hydrogen peroxide in wild type- and Δ*bcvel1*-infected plant tissues, infected bean leaves were sampled at several time points (24, 52, and 72 hpi) and incubated for two hours in DAB solution. Staining became macroscopically visible from 24 hpi and further intensified at later time points. At 24 hpi, infections with either strain caused a similar staining pattern (Fig. 7). Spreading lesions at 52 hpi and later time points became brownish and were surrounded by zones of massive hydrogen peroxide accumulation. Remarkably, non-spreading lesions of the Δ*bcvel1* mutant were bounded by more defined zones of hydrogen peroxide accumulation after 72 h of incubation. As fungal hyphae outside of the restricted areas were never observed (data not shown), the accumulation of plant-derived hydrogen peroxide might be involved in preventing lesion spreading of Δ*bcvel1*.

Previously, we have shown that the two groups of phytotoxins, the sesquiterpenoid botryanes (BOT) and the polyketide-derived botcinins (BOA) have impact on virulence [7], [8], [9]. However, analyses of the metabolite compositions in the culture media of the wild type and the Δ*bcvel1* mutant by TLC, HPLC, and ^1^H and ^13^C NMR measurements did not reveal any differences (Table 2). To demonstrate the activity of the toxin gene clusters *in planta*, we analyzed the expression levels of one gene per gene cluster (*bcbot1* for the BOT cluster and *bcboa3* for the BOA cluster) in primary lesions of the wild type and the Δ*bcvel1* mutant. In accordance with the chemical data obtained from axenic cultures, both gene clusters were comparably expressed in both strains *in planta* (Fig. 6B) indicating that BcVEL1 does not regulate the production of these phytotoxic secondary metabolites. In addition, we compared the expression levels of genes formerly shown to contribute to full virulence. Both *bcxyn11A* and *bcspl1* encode proteins exhibiting necrotizing activities [11], [12], [14] and were similarly expressed in both strains. Surprisingly, *bcoahA* encoding the OA-producing enzyme that was shown to be under-expressed in the Δ*bcvel1* mutant in axenic culture (Fig. 3B), was expressed *in planta*.

**Table 2:** Production of phytotoxic metabolites by Δ*bcvel1* mutants and wild type B05.10.

Metabolites	WT:B05.10	Δbcvel1
Crude extract	81.0 mg	80.0 mg
Botrydial	5.0 mg	4.5 mg
Dihydrobotrydial	1.5 mg	1.0 mg
Botryendial	2.0 mg	2.5 mg
10-oxodihydrobotrydial	1.2 mg	0.8 mg
Botryenalol	2.8 mg	2.0 mg
Botcinin A	6.0 mg	7.0 mg

**Figure 8:**
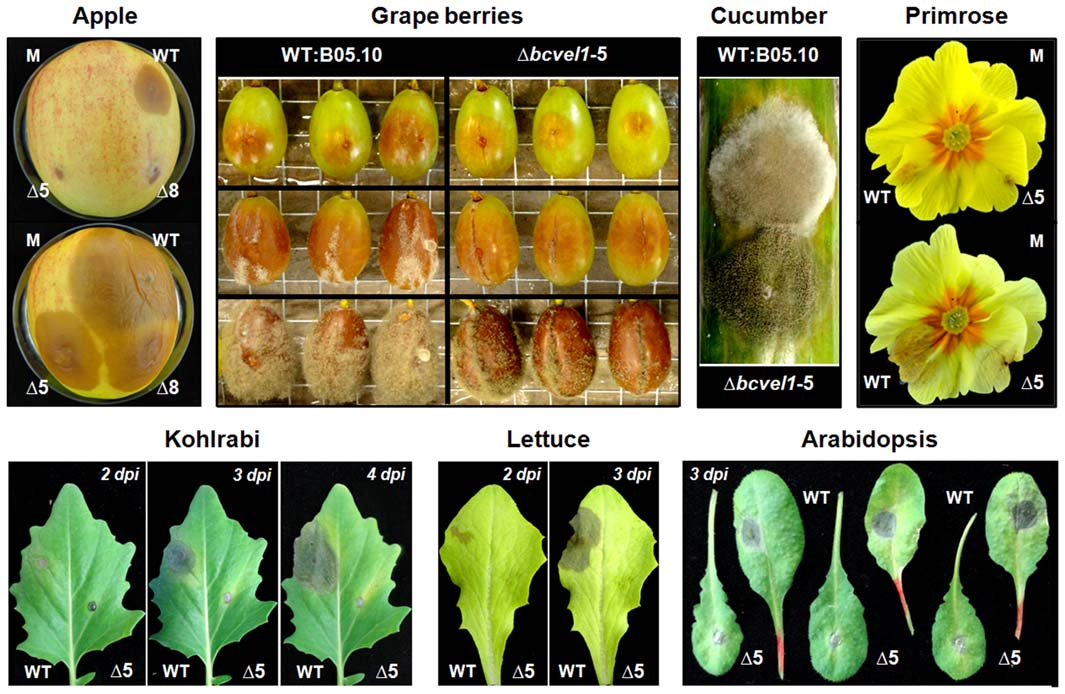
Disease symptoms on other plant tissues. All plant tissues were inoculated with conidial suspensions of strains WT:B05.10 and Δ*bcvel1* (two independent mutants, Δ5 and Δ8). Surfaces of apple, cucumber and grape berries were wounded with a needle prior to inoculation. Pictures of infected apple fruit were taken 5 and 8 dpi, of infected grape berries 3, 6 and 9 dpi, cucumber 7 dpi, and of the infected primrose flower 2 and 4 dpi. Leaves of young kohlrabi and lettuce plants were detached prior to inoculation while leaves of *Arabidopsis thaliana* Col-0 plants were separated 3 dpi. M, mock-treated.

To see whether reduced virulence is a general feature of *bcvel1* mutants, other host tissues were inoculated with conidial suspensions. On unwounded leaves of kohlrabi, lettuce and *A. thaliana*, results similar to the infection of bean leaves were obtained: the mutants were able to cause small necrotic spots but failed to spread further into the tissue and to produce conidia (Fig. 8). Retarded infection of *bcvel1* mutants was also observed on wounded apple fruits and grape berries. Notably, *bcvel1* mutants produced conidia on infected grape berries after 9 days of incubation but not on apple. Infection of cucumber by *bcvel1* mutants was characterized by the massive production of conidia while the wild type predominantly formed aerial mycelium. Milder disease symptoms for *Δbcvel1* mutants were also observed on primrose flowers (Fig. 8).

Taken together, BcVEL1 is localized in the nuclei during the infection process, is dispensable for penetration of host cells but is essential for invasive growth and reproduction on host tissues. Even though the deletion mutant retains the full set of known virulence factors (phytotoxic secondary metabolites and proteins exhibiting necrotizing activities), it is unable to efficiently kill host cells and to macerate plant tissue. Furthermore, the virulence defect is not restricted to a single host plant, although certain variations were found with regard to the ability of the mutant to form conidia on the different plant tissues.

### Microarray analysis reveals BcVEL1-dependent expression profiles during infection

**Figure 9:**
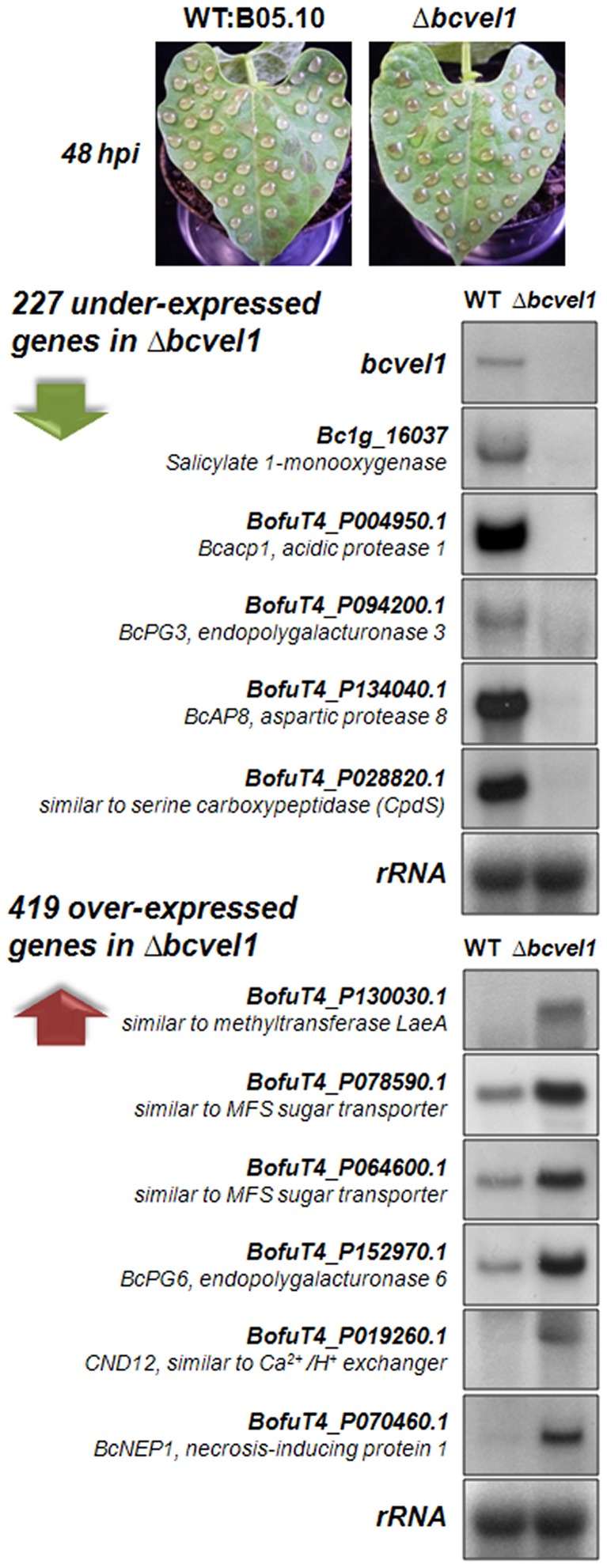
Analysis of the BcVEL1-dependent expression profile during infection. Primary leaves of living *P. vulgaris* plants were inoculated with many droplets of conidial suspensions of the wild-type strain B05.10 and the Δ*bcvel1* deletion mutant, respectively. Infected plant material (from four biological replicates) was sampled at 48 hpi before infections started to spread. RNA was extracted, labeled and hybridized to *B. cinerea* microarrays. The comparative analysis of the array data revealed 227 genes that were under- and 419 genes that were over-expressed in the Δ*bcvel1* mutant (for details see Tables S1, S2; Fig. S9). Expression profile of some arbitrarily chosen genes was confirmed by northern blot analyses.

To gain broader insight into the function of BcVEL1 during infection, we performed a microarray analysis using infected plant material. For that, primary leaves of *P. vulgaris* were inoculated with droplets of conidial suspensions of the wild type B05.10 and the *bcvel1* deletion mutant (Fig. 9) and harvested 48 hpi. At this time, both strains were at the same stage of infection (before the onset of lesion spread), allowing a direct comparison of expression profiles of both interactions that were supposed to reveal relevant information on cellular processes involved in subsequent lesion spreading. RNA from four biological replicates was extracted, labeled and hybridized to NimbleGen microarrays containing oligonucleotides representing all predicted *B. cinerea* genes [47]. Hybridization quality controls highlighted variations between the four biological replicates. Accordingly, control plants that were infected in parallel with two inoculation droplets per leaf for monitoring the later infection stages, showed different disease symptoms for Δ*bcvel1* infections after 7 dpi, i.e. stop in the primary stage or pass over to secondary spread (data not shown). Nevertheless, an ANOVA test was performed to detect genes differentially expressed between wild type and Δ*bcvel1* strains irrespective of the assumed late symptom. The comparative analysis revealed 227 genes that were under- and 419 genes that were over-expressed in the Δ*bcvel1* mutant (Tables S1, S2). The expression profile of some representative genes belonging to both groups was confirmed by northern blot analyses (Fig. 9).

Functional enrichment analyses for the differentially expressed genes were performed using the GOEAST tool (Fig. S9). These analyses revealed that the group of under-expressed genes is enriched with those involved in proteolytic processes (Fig. S9, Table S1). The large group of over-expressed genes is enriched with those involved in transmembrane transport and carbohydrate modification. Thus, several over-expressed genes encode putative MFS (major facilitator superfamily) sugar transporter, amino acid transporter, MFS multidrug transporter and glycoside hydrolases (Table S2). Notably, all three genes involved in nitrate metabolism (nitrate transporter, nitrate reductase, and nitrite reductase) are over-expressed in the deletion mutant. To examine whether this deregulation of nitrate metabolism happens also under axenic growth conditions, the wild type and several mutant strains were grown on solid medium supplemented with potassium chlorate. Chlorate is converted by the nitrate reductase to toxic chlorite, and therefore it can be used as an indicator for nitrate reductase activity [54]. Indeed, reduced growth rates in presence of chlorate for the *bcvel1* loss-of-function mutants were found indicating the up-regulation of the nitrate-metabolizing enzymes also under axenic conditions (Fig. S10).

Among the differentially expressed genes are many genes whose functions are still unknown and some of them might be unique to *B. cinerea*. For instance, we identified a genomic region comprising eight predicted genes that are over-expressed in *bcvel1* mutants *in planta* (Fig. S11A). One of these genes likely encodes a transcription factor; the other genes encode relatively small proteins of unknown functions containing either putative signal peptides or transmembrane domains (Table S3). Another cluster of three genes predicted to be responsible for the biosynthesis of an unknown polyketide (BcPKS7 cluster) [47] is under-expressed in *bcvel1* mutants (Table S1). Interestingly, these two BcVEL1-dependent gene clusters are absent from the genome of the close relative *S. sclerotiorum* (Fig. S11B) [47].

## Discussion

Natural populations of *B. cinerea* show extensive genetic variation for a wide range of factors that might affect host range, mode of reproduction, and fungicide resistance. In this study, we explored the genetic basis for distinct phenotypes in light-dependent differentiation, OA formation and virulence in isolates B05.10 and T4. Even though the alignment of the genome sequences of B05.10 and T4 revealed a total of 98,744 insertion/deletion positions and 175,009 SNPs [47], we could demonstrate by several approaches that a single point mutation in the VELVET gene *bcvel1* is responsible for the observed phenotypic differences: the deletion of *bcvel1* in B05.10 resulted in the T4 phenotype, and in turn, the replacement of the truncated *bcvel1* copy by the native copy in T4 resulted in the B05.10 phenotype. In addition, the expression of the T4 copy in B05.10Δ*bcvel1* background failed to restore the wild type phenotype.

BcVEL1 constitutes the homologue of *A. nidulans* VeA for that the first mutant (*veA1*) was described by Käfer [55]. This mutant contains a point mutation in the start codon of *veA* resulting in an N-terminally truncated protein lacking the first 36 amino acids [22]. Like the C-terminally truncated BcVEL1^T4^ the mutated VeA1 protein is located in the cytosol rather than in nuclei [56], [57] indicating that the motifs essential for nuclear localization are situated in the C- and the N-terminal parts of the two proteins, respectively. In fact, a bipartite N-terminal NLS (aa 28–44) is present in VeA and was shown to be crucial for nuclear localization in *A. nidulans* [56] while the functional NLS of the VeA homologue in *A. chrysogenum* is localized in the C terminus [30] and an obvious NLS is absent from the *B. cinerea* homologue. Noteworthy, subcellular localization of VeA in *A. nidulans* was shown to be light-dependent; in the dark, VeA is translocated to the nucleus while in the light, it is abundantly found in the cytosol [56]. We did not observe such a light dependency for the subcellular localization of the full-length BcVEL1, as it was shown previously for the FfVEL1-GFP in *F. fujikuroi* [32].

In *F. fujikuroi vel1* deletion mutants, the formation of the red pigment bikaverin is up-regulated while the production of GAs, a family of plant hormones, as well as the mycotoxins fusarin C and fumonisins are lost or down-regulated. Fungal GA production is considered to contribute to virulence of *F. fujikuroi* on rice seedlings by inducing abnormal elongation of plant internodes. In accordance, Δ*ffvel1* mutants lacking GA formation failed to induce typical disease symptoms [32]. Interestingly, the expression of *bcvel1* in the *F. fujikuroi* mutant rescued the developmental phenotypes (aerial hyphae and microconidia formation) and the wild type-like pigment formation but not the formation of GAs, and subsequently not virulence. The fact that some but not all phenotypes could be rescued by the *B. cinerea* gene may indicate that different regions of the proteins (the highly conserved *VELVET* domain or the less conserved C terminus) are required for interacting with other components of the VELVET complex and subsequently for regulating differentiation processes and fungus-specific secondary metabolism in ascomycetous fungi.

Differentiation processes in *B. cinerea* are strictly regulated by the light regime: while light induces conidiation, the absence of light results in sclerotial development. Consistent with other fungi that show similar light responses, the deletion of the VeA homologue in *B. cinerea* uncouples the developmental program from this light control. Hence, *bcvel1* deletion mutants exhibited light-independent conidial formation resulting in the loss of both the typical “banding pattern” in light-dark conditions and sclerotia formation in constant darkness. The fact that sclerotia function as survival structures and as the “female parent” during sexual reproduction indicates the importance of BcVEL1 for survival of the pathogen and for achieving genetic recombination in the field. Furthermore, *bcvel1* mutants accumulate increased amounts of melanin that is an important component of the extracellular matrix of conidia, germlings and sclerotia [58], [59]. As melanin is incorporated in survival structures possibly for protecting cells against UV light and oxidizing agents [52], conidiation and melanin formation might be coordinated by light via BcVEL1; hence, the deletion of *bcvel1* results in increased formation of conidia and melanin independent of the light conditions. Excessive pigment formation by *VELVET* mutants was also observed for other ascomycetes, e.g. for *F. fujikuroi* [32], *C. heterostrophus* [39], and *A. nidulans* [31], while the deletion of the VELVET homologue in other species resulted in loss of pigment formation as shown in *M. graminicola* [35], *F. graminearum* [36], [37], and *T. virens* [34].

Like other necrotrophic plant pathogens, *B. cinerea* produces complex secondary metabolites exhibiting phytotoxic activities, e. g., the botryanes and the botcinins. Noteworthy, B05.10 and T4 isolates were previously shown to differ in their potential to form these compounds: while both groups of toxins are produced by B05.10, T4 only produces botrydial-like toxins [7]. VeA homologues are described as global regulators of secondary metabolite gene clusters in diverse fungi, and in fact, the impairment of secondary metabolism by deletion of the VeA homologue affects virulence in some but not all pathogens producing toxic compounds. Hence, fumonisin-deficient *F. verticillioides* Δ*fvve1* mutants cause symptomless endophytic infections when plants were grown from inoculated seeds [28], and loss of thrichothecene and T-toxin production in *F. graminearum* and *C. heterostrophus* mutants, respectively, is accompanied by reduced virulence [36], [37], [39]. In contrast, the deletion of VeA in *D. septosporum* resulted in reduced formation of dothistromin but not in reduced virulence [38]. Therefore, we had hypothesized an impact of the *bcvel1* deletion on the formation of the two known groups of toxins. Surprisingly, the production of both toxins was not affected by the *bcvel1* deletion in strain B05.10, neither *in vitro* nor *in planta*. Currently, only these two metabolite groups have been investigated in detail by functional and chemical analyses. However, the genome of *B. cinerea* comprises approximately 40 genes encoding key enzymes of secondary metabolism, such as polyketides, non-ribosomal peptides and terpenes. Some of these *B. cinerea*-specific genes are highly expressed during infection of sunflower cotyledon, grape berries or bean leaves and one of them, *bcpks7*, appeared to be BcVEL1-dependent [47] (Viaud *et al.*, unpublished data; this study). However, whether the corresponding metabolites are associated with virulence remains to be investigated.

Oxalic acid (OA) is a compound that is produced by numerous filamentous fungi, including the *A. niger*, *A. fumigatus* and the plant pathogens *B. cinerea* and *S. sclerotiorum*. Although OA is derived from primary metabolism, it can be considered as a secondary metabolite as it is not required for the survival of the organisms but it might be associated with the pathogenic lifestyle in some fungi. Thus, the loss of OA formation in *S. sclerotiorum* results in non-pathogenic mutants [60], while an OA-deficient *B. cinerea* strain is still able to infect plant tissues [61]. The different impact of OA on virulence of both fungi might be associated with different pH dynamics during plant tissue colonization. Billon-Grand and co-workers recently showed that *S. sclerotiorum* decreases the ambient pH value and remained in an acidic environment while *B. cinerea*-colonized tissue established a final neutral environment after temporary lowering the pH at 48 hpi. The increase of the pH value in *B. cinerea*-infected tissue can be assigned to a much lower OA formation accompanied by an enhanced formation of ammonia while in *S. sclerotinia*-infected tissues both OA and ammonia formation increase simultaneously maintaining acidic conditions [62]. OA is a versatile compound that may modulate fungus-host interactions in different ways. The acidification of surrounding tissue may stimulate the production and the activity of secreted enzymes, and OA may sequester calcium from plant cell walls thereby leading to their destabilization. Furthermore, OA was shown to reduce the oxidative burst and other defense responses in plant tissues and to directly trigger programmed plant cell death [63], [64]. For growth in vitro, OA secretion by *B. cinerea* is essential for allowing growth on medium with neutral/alkaline pH values. Accordingly, the *bcvel1* mutants that do not express the OA-generating enzyme [51] are unable to grow in a wild-type-like manner on plates with alkaline pH. However, even if the gene (*bcoahA*) is drastically under-expressed in the *bcvel1* mutant compared to the wild type *in vitro*, the gene is highly expressed in both strains *in planta*. Therefore, neither the loss of toxin formation nor the loss of OA production is responsible for reduced virulence of the *bcvel1* mutant. The expression of *bcpks13* encoding the key enzyme of the melanin-biosynthetic pathway is up-regulated *in vitro* and non-detectable *in planta*, indicating different modes for regulation of OA and melanin formation during growth in axenic culture and infection of host tissues.

Despite the wild type-like *in planta* expression of toxin biosynthetic genes, the *bcvel1* mutant exhibits a reduced capacity to colonize plant tissues. Notably, outcomes of the infection were highly variable: either infections stopped in the primary stage or continued as spreading lesions that differ from wild-type lesions by significantly reduced numbers of dead plant cells. We postulate that minor changes in growth conditions of the bean plants are responsible for the unstable infections by the *bcvel1* mutant while wild-type infections are not affected due to the unchanged full capacity to express all needed virulence factors. Studies in Arabidopsis showed that nutritional and light conditions can control the outcome of an infection by acting on the host's defense responses e.g. on callose deposition [65], [66], and a fungal pathogen with severe defects in development and/or metabolism as the *bcvel1* mutant might be much more sensitive to such changes.. In accordance with the altered infection pattern of *bcvel1* mutants we found many differentially expressed genes in wild-type and *bcvel1*-infected bean leaves. Besides a large number of genes with unknown functions the analysis revealed that the BcVEL1-dependent genes are significantly enriched in putatively virulence-associated genes. Remarkably, the group of *Δbcvel1* under-expressed genes is enriched for secreted enzymes with proteolytic activities (serine and aspartic proteases). Several of these proteases (e.g. *bcacp1/SSH26* – acidic protease, *SSH22* – metalloprotease, *bcap8/SSH22* – aspartic protease) have been identified before as differentially expressed in wild type- and Δ*bcg1*-infected plant tissues. Infection by Δ*bcg1* mutants that lack one of the three α subunits of heterotrimeric G proteins always stops before onset of spreading, and was shown to be associated with reduced expression levels of xylanase- and protease-encoding genes as well as genes coding BOA- and BOT-biosynthetic enzymes [67], [68], [7], [9]. Furthermore, a non-pathogenic mutant (A336) generated by random mutagenesis, was shown to be impaired in *in vitro* OA formation and *bcacp1* expression [69], suggesting the importance of proteases for invasion of the plant tissue. Proteolytic enzymes may have different functions, e.g. they may play a crucial role in nutrition by external digestion of macromolecular nutrients, or they may be involved in compromising the host defense system by degradation of plant defense proteins such as pathogenesis-related (PR) proteins. Expression of extracellular proteases in fungi is regulated in response to environmental signals such as carbon and nitrogen availability or extracellular pH [70]. All these factors are likely subjects to variation throughout the infection process of *B. cinerea* and the transcriptional regulation of protease-encoding genes (*bcacp1*, *bcap8*) by ambient pH has been previously shown [68], [71], suggesting that expression of these proteases may depend on pH dynamics during infection. Hence, acidic proteases might be expressed during the first steps of colonization that is accompanied by an ambient pH decrease, while the pH increases during the later stages may facilitate the expression of serine proteases whose activities are observed only at alkaline pH [70]. The loss of a single protease likely will not affect virulence as shown for the deletion of *bacp8* [72]. The down-regulation of a whole set of proteases, however, may very well explain the reduced ability of *bcvel1* mutants to colonize plant tissues.

The group of over-expressed genes in *bcvel1* mutants mainly comprises those predicted to be involved in nutrient acquisition. Thus, several genes encoding MFS sugar transporters, ammonium transporters and nitrate and amino acid transporters showed increased expression levels suggesting that the Δ*bcvel1* mutants sense nutrient starvation conditions possibly due to its inability to kill plant cells and to deconstruct plant tissue in a wild-type-like manner. Furthermore, the over-expression of several MFS multidrug transporter-encoding genes in the *bcvel1* deletion mutant could reflect an elevated response to plant defense responses, as active efflux by ABC and MFS transporters may provide resistance to toxic compounds such as antibiotics, plant defense compounds and fungicides [73], [74]. However, BcAtrB known to export the phytoalexins camalexin and resveratrol and therefore being essential for full virulence on Arabidopsis and grape vine [75], [76], does not belong to the set of differentially expressed transporters in this approach.

To determine whether mutations in *bcvel1* also occurred in other isolates, we screened several natural isolates of different origins for their capacity to produce OA and to form sclerotia during incubation in constant darkness. We found that eight out of 70 isolates tested were also affected in these traits. Sequencing of *bcvel1* in these eight isolates revealed the presence of several SNPs, one of them resulting in an early stop codon and consequently in a truncated protein shorter than that of isolate T4 (data not shown). These data support our suggestion that mutations of *bcvel1* are quite common thereby contributing to genetic variation in field populations. Even though the other seven OA- and sclerotia-deficient isolates did not contain mutations in BcVEL1, their phenotype might be associated with mutations of BcVEL2 (VelB homologue) as similar functions have been described for VeA/VelB homologues in several fungi [57], [32].

In conclusion, our results provide evidence that a single point mutation in the natural *B. cinerea* isolate T4 is responsible for the deregulation of light-dependent development, loss of OA formation and reduced virulence compared to strain B05.10. The resultant truncated BcVEL1 led to reduced fitness of strain T4 and at least of one additional natural isolate. The persistence of these deleterious mutations is not yet understood and remains to be elucidated. One hypothesis is that the formation of conidia even in the absence of light may, in some particular ecological niches e.g. on some hosts, confer a fitness advantage in terms of higher survival rates and spatial distribution of the fungus over the ability to form sclerotia as survival structures and full virulence on all host plants.

## Materials and Methods

### *B. cinerea* strains and growth conditions

Strain B05.10 of *B. cinerea* Persoon: Fries [*Botryotinia fuckeliana* (de Bary) Whetzel] is an isolate from *Vitis* [77], [78] and is used as the recipient strain for gene replacement analyses. Strains T4 (low aggressiveness) and 32 (aggressive) were isolated in France in a tomato glasshouse and from *Vitis*, respectively [47]. Genome sequences of B05.10 and T4 were recently published (http://urgi.versailles.inra.fr/Species/Botrytis) [47]. All isolates and mutants used in this study are listed in Table 1. Strains were cultivated on plates containing synthetic solid complete medium (CM) [79] or solid malt medium (2% malt extract, 0.2% yeast extract, 1.5% agar). The strains were incubated at 20°C under full-spectrum light (12 h light/12 h darkness (LD) or continuous light (LL)) for conidiation, and in continuous darkness for sclerotia formation. For DNA and RNA isolation, mycelia were grown for 3 days on CM agar with a cellophane overlay. For monitoring the production of melanin, conidia were incubated at 20°C and 150 rpm in 100 ml of liquid medium [0.2% yeast extract, 1.0% glucose, 0.2% KH_2_PO_4_, 0.15% K_2_HPO_4_, 0.1% (NH_4_)_2_SO_4_, 0.05% MgSO_4_·7 H_2_O]. Later steps in the melanin biosynthetic pathway were inhibited by adding 10 µg/ml tricyclazole (Sigma-Aldrich, Germany). Crosses between strains were conducted according to Faretra and Antonacci [18]. Briefly, strains were incubated for 4 weeks in darkness at 18–20°C and then for 4 weeks at 4°C, allowing the isolation of sclerotia and microconidia from the same cultures. Sclerotia were picked and cleaned from mycelia, microconidia were washed and filtered. Then, sclerotia and microconidia derived from strains carrying opposite mating types (see Table 1) were mixed and incubated at 10°C under full-spectrum light (12 h light/12 h darkness) until apothecia emerged.

### Standard molecular methods

Fungal genomic DNA was prepared according to Cenis [80]. For Southern blot analysis, fungal genomic DNA was digested with restriction enzymes (Fermentas, Germany), separated on 1% (w/v) agarose gels and transferred to Amersham Hybond-N+ filters (GE Healthcare Limited, UK). Total RNA was isolated making use of the TRIzol reagent (Invitrogen, The Netherlands). cDNA from total RNA was synthesized using the oligo(dT)12-18 primer and the SuperScript II reverse transcriptase (Invitrogen, The Netherlands) according to the manufacturer's instructions. Samples (25 µg) of total RNA were transferred to Hybond-N+ membranes after electrophoresis on a 1% (w/v) agarose gel containing formaldehyde, according to the method of Sambrook et al. [81]. Blot hybridizations with random-primed α-^32^P-dCTP-labelled probes were performed as described previously [82].

PCR reactions were performed using the high-fidelity DNA polymerase Phusion (Finnzymes, Finland) for cloning purposes and the BioTherm™ Taq DNA Polymerase (GeneCraft, Germany) for diagnostic applications. Replacement fragments and expression vectors were assembled in *Saccharomyces cerevisiae* by exploiting its homologous recombination machinery as described previously [83], [84]. Sequencing of DNA fragments was performed with the Big Dye Terminator v3.1 sequencing kit (Applied Biosystems, USA) in an ABI Prism capillary sequencer (model 3730; Applied Biosystems). For sequence analysis, the program package DNAStar (Madison, USA) was used. Protocols for protoplast formation and transformation of *B. cinerea* were adapted from an established procedure [67]. After 24 h, the regenerated protoplasts were overlaid with SH agar containing 70 µg/ml hygromycin B (Invitrogen, The Netherlands) and 140 µg/ml nourseothricin (Werner-Bioagents, Germany), respectively. Resistant colonies were transferred to agar plates containing Gamborg's B5 medium (Duchefa Biochemie BV, The Netherlands) supplemented with 2% glucose, 0.1% yeast extract and the respective selection agent in a concentration of 70 µg/ml.

### Gene mapping

The 68 ascospore isolates obtained from a cross between isolates T4 (*MAT1-2*) and 32 (*MAT1-1*) are presented in Amselem et al. [47]. The ability of progenies to form sclerotia (marker *Bcscl*) was tested in three independent cultures. The virulence of the progenies was compared to those of the parental strains using French bean (*P. vulgaris* cv. Caruso). Leaves were harvested from 2-week-old plants and placed in a transparent plastic box lined with moistened tissue. For each isolate, three leaves were inoculated with plugs (diameters of 1.8 mm) of 3-day-old mycelium and were incubated at 21°C under full-spectrum light conditions (16 h light/8 h darkness). Disease development on leaves was recorded 4 dpi. Progenies that gave lesion sizes similar to that of the parental strain 32 (diameter of 31±5 mm) were noted as fully virulent; while progenies that provoked lesion sizes similar to that of the parental strain T4 (diameter of 12±8 mm) were considered as impaired in virulence. The ability of the progenies to secrete OA and therefore to acidify the culture medium was monitored on solid CM pH 7.5 supplemented with 0.1% bromothymolblue as a pH indicator. Polymorphic microsatellite sequences were searched in the genome of strains T4 (http://urgi.versailles.inra.fr/index.php/urgi/Species/Botrytis) and B05.10 (http://www.broadinstitute.org/annotation/genome/botrytis_cinerea/Home.html) using the GRAMENE software [85]. The designed flanking primers are indicated in Table S4. PCR products generated for the parental strains (T4 and 32) and progenies were run on a 3% agarose gel. The segregation of the phenotypic and molecular markers among the progeny was analysed using the MAPMAKER software [86] set at min LOD3 and max Distance at 37.

### Identification and annotation of the *B. cinerea VELVET* genes

*Bcvel1* corresponds to annotated genes BofuT4_P003460.1 and BC1G_02976.1/BC1G_02977.1 in the T4 and B05.10 databases, respectively. As the sequences and consequently the gene models presented in the two databases were differing, the gene locus was re-sequenced in both isolates. While the sequencing of DNA and cDNA clones derived from B05.10 showed that a base pair is missing in the published sequence of B05.10 resulting in a frame shift and the interruption of the open reading frame (ORF) due to a stop codon, the sequence of T4 could be confirmed. However, gene model BofuT4_P003460.1 comprises an incorrectly annotated intron. Taken together, the ORF of *bcvel1* in B05.10 (*bcvel1^B05.10^*) contains 1,794 bp and is interrupted by one intron of 66 bp, resulting in a protein of 575 aa (HE977589). *Bcvel1* in T4 bears a SNP that leads to a stop codon and therefore in the termination of translation. Hence, *bcvel1^T4^* comprises 621 bp including the 66-bp intron and encodes a protein of 184 aa (HE977590). Other *VELVET* proteins in *B. cinerea* were searched in the protein databases of T4 and B05.10 by performing BlastP analyses (http://blast.ncbi.nlm.nih.gov/Blast.cgi) using the sequence of BcVEL1^B05.10^ as the query. Three other proteins containing the VELVET protein domain were identified and named BcVEL2 to BcVEL4. *Bcvel2* corresponds to predicted genes BofuT4_P161180.1/BC1G_11858.1. According to the revised annotation, its ORF comprises 1,768 bp and is interrupted by six introns (107 bp, 75 bp, 49 bp, 65 bp, 58 bp, 58 bp). The deduced protein contains 451 aa (HE977591). *Bcvel3* corresponds to predicted proteins BofuT4_P017230.1/BC1G_06127.1. Exon-intron structures of the presented gene models were revised. Hence, the ORF contains 1,488 bp and three introns (51 bp, 48 bp, and 102 bp) resulting in a protein of 428 aa in B05.10 (HE977592) and 427 aa in T4 (HE977593). *Bcvel4* corresponds to predicted proteins BofuT4_P157800.1/BC1G_11619.1. The final annotation predicts an ORF of 1,235 bp that is interrupted by one intron of 53 bp; the protein comprises 393 aa (HE977594). Conserved domains in the proteins sequences of BcVEL1 to BcVEL4 were identified using InterProScan (http://www.ebi.ac.uk/Tools/pfa/iprscan/), putative nuclear localization signals (NLS) were determined using WoLF PSORT (http://wolfpsort.org/), putative nuclear export sequences (NES) were identified using the NetNES 1.1 server (http://www.cbs.dtu.dk/services/NetNES/), and putative PEST domains were predicted using the EMBOSS program Epestfind (http://emboss.bioinformatics.nl/cgi-bin/emboss/epestfind).

### Construction of *bcvel1* mutants

The replacement construct for *bcvel1* was generated using the homologous recombination system in yeast according to Colot et al. [83]. Therefore, the 5′- and 3′-non-coding regions of *bcvel1* were amplified from genomic DNA using the primer pairs *bcvel1*-5F/*bcvel1*-5R and *bcvel1*-3F/*bcvel1*-3R, respectively (Fig. S4A, Table S5). The hygromycin resistance cassette was amplified with primers *hph*F and *hph*R using pCSN44 as a template. Fragments were co-transformed with the linearized vector pRS426 into uracil-auxotrophic *S. cerevisiae* strain FY834 for assembly. The replacement construct (3,419 bp) was amplified using primers *bcvel1*-5F and *bcvel1*-3R and used for transformation of *B. cinerea* B05.10. Homologous integration events in hygromycin-resistant transformants were detected by diagnostic PCR using the primers pCSN44-trpC-T and pCSN44-trpC-P, binding in the hygromycin resistance cassette and the primers *bcvel1*-hi5F and *bcvel1*-hi3R, binding upstream and downstream of the *bcvel1* flanking regions. Single spore isolates were screened for the absence of *bcvel1* alleles using primers *bcvel1*-WT-F and *bcvel1*-WT-R. Taken together, three independent deletion mutants of *bcvel1* (T5, T8, T22) in the B05.10 background were generated (Fig. S4B). To show the absence of further ectopic integrations in these mutants, the genomic DNA was digested with *Eco*RI, blotted, and hybridized with the 5′ flank of *bcvel1*. As expected single hybridising fragments of 2.0 kb were obtained for the *bcvel1* deletion mutants while a hybridising fragment of 12.0 kb was obtained for the recipient strain B05.10 (Fig. S4C). As identical phenotypes for the different deletion mutants were found, the results for one mutant (Δ*bcvel1*-T5) are presented. For the expression of BcVEL1-GFP fusion proteins, the two different gene copies (1,794 bp for B05.10 and 621 bp for the T4) were amplified using primer pairs *bcvel1*-GFP-F/B05.10-GFP-R and *bcvel1*-GFP-F/T4-GFP-R (Fig. S4A), respectively, and assembled with the *Nco*I-digested plasmid pNAN-OGG comprising gene flanks for targeted integration (replacement of *bcniiA* encoding the nitrite reductase), a nourseothricin resistance cassette and the expression cassette with *gfp* under control of the constitutive *oliC* promoter [84]. Resulting *bcvel1-gfp* constructs were transformed into the wild-type strain B05.10 and the *bcvel1* deletion mutant (Δ*bcvel1*-T5). Integration of the constructs at the *bcniiA* locus was verified by diagnostic PCR as described previously [84] (Fig. S4D). Three independent transformants per construct were inspected for GFP fluorescence and phenotypes. The results for one transformant per construct are shown. Two different strategies for complementation of *bcvel1* loss-of-function mutants were followed up. The first one included the targeted integration of *bcvel1* (ORF+800 bp and 240 bp of the 5′- and 3′-non-coding regions) at the *bcniaD* locus resulting in the replacement of the gene encoding the nitrate reductase. For this, the PCR fragment generated using primers *bcvel1*-COM-F and *bcvel1*-COM-R was digested with the endonucleases *Apa*I and *EcoR*V and cloned into the *Apa*I/*Sma*I-digested plasmid pΔ*bcniaD*-natR yielding vector p*bcvel1*-COM*^bcniaD^*. The second strategy arranged the targeted integration of *bcvel1^B05.10^* at the native *bcvel1* locus resulting in the replacement of the hygromycin resistance cassette in Δ*bcvel1* mutants or the mutated *bcvel1^T4^* copy in the T4 background. For that, the ORF of *bcvel1* including the 5′-flank and a part of the 3′-flank was amplified with primers *bcvel1*-5F and *bcvel1*-COM-5R, the second part of the former 3′-flank with primers *bcvel1*-COM-F and *bcvel1*-3R and the nourseothricin resistance with primers *hphR-trpC*-T2 and *hph*F using plasmid pZPnat1 (AY631958.1) as template. Amplicons were assembled with the linearized plasmid pRS426 in yeast yielding vector p*bcvel1*-COM-iL (Fig. S4A). Both constructs were transformed into strains Δ*bcvel1*-T5 and T4, and targeted integration was verified by diagnostic PCR and sequencing. Data shown in Fig. 3, 5, S4E, S11 correspond to mutants carrying the *bcvel1* complementation construct at the native gene locus. By integration of the complementation construct at the *bcniaD* locus, virulence and OA production but not sclerotia formation could be restored possibly due to the ectopic integration of the construct or the use of only 800 bp of the *bcvel1* promoter region (data not shown). Expression of *bcvel1* in the different mutants (one mutant per construct) was detected by northern blot analyses (Fig. S4E).

### Toxin production

For analysis of metabolite production, strains were grown on malt agar medium, (2% glucose, 1% malt extract, 2% agar, 0.1% peptone, pH 6.5–7) at 25°C; and used to inoculate Petri dishes, ∅ 150 mm, with 100 ml of solid malt medium. Six Petri dishes were inoculated with three mycelial plugs (9 mm ∅) each and incubated for 15 days at 25°C. Then the solid malt medium was cleaned from mycelia and conidia and was extracted with ethyl acetate (3×0.5 vol) using an ultrasonic bath (30 min). The organic extracts were dried over Na_2_SO_4_ and concentrated to dryness to yield dark oil extract, 80–81 mg approximately. ^1^H and ^13^C NMR measurements on metabolites isolated from culture extracts were obtained on Varian Unity 400 and Varian Innova 600 NMR spectrometers with SiMe_4_ as the internal reference. Mass spectra were recorded on a GC-MS Thermoquest Voyager spectrometer, and a VG Autospec-Q spectrometer. HPLC was performed with a Hitachi/Merck L-6270 apparatus equipped with a UV-VIS detector (L 6200) and a differential refractometer detector (RI-71). TLC was performed on Merck Kiesegel 60 F_254_, 0.2 mm thick. Silica gel (Merck KGaA, Germany) was used for column chromatography. HPLC purification was accomplished with a silica gel column (Hibar 60, 7 m, 1 cm wide, 25 cm long). Chemicals were products of Fluka or Aldrich. All solvents were freshly distilled. For metabolite isolation and characterization, the dark oil extracts obtained from cultures of *B. cinerea* strains B05.10 and Δ*bcvel1*, respectively, were separated by column chromatography on silica gel, with a mixture of ethyl acetate/hexane (10, 20, 40, 60, 80 and 100% ethyl acetate), and 20% methanol in ethyl acetate as solvent. Extensive spectroscopic analysis by ^1^H-NMR and ^13^C-NMR were used to detect the presence of the various toxins in each fraction. Candidate fractions were further purified by HPLC with an increasing gradient of ethyl acetate to petroleum ether. The toxin structures were analyzed by spectroscopic methods and direct comparison with authentic samples, previously isolated from strains of *B. cinerea* [87], [88].

### Germination assays

Germination rates of conidia from *B. cinerea* strains were monitored according to Doehlemann et al. [49]. For testing nutrient-dependent germination and hydrophobicity-induced germination, cleaned conidia were incubated in Gamborg's B5+10 mM glucose on glass surfaces and in double distilled water on polypropylene foil, respectively.

### Virulence assays

Penetration assays on epidermal layers were performed as described previously [84]. For infection assays, the conidia were re-suspended in Gamborg's B5 medium supplemented with 2% glucose and 10 mM KH_2_PO_4_/K_2_HPO_4_, pH 6.4. Droplets (7.5 µl) of conidial suspensions (2×10^5^ conidia/ml) were used to inoculate various plant tissues including primary leaves of French bean (*Phaseolus vulgaris* cv. 90598), leaves of *Arabidopsis thaliana* Col-0 (provided by A. von Schaewen, WWU Münster, Germany), and grape berries (*Vitis vinifera*, cv. Thompson Seedless). Surfaces of the fruits were wounded with a needle prior to inoculation. Infected plant tissues were incubated in humid conditions at 20°C under natural illumination. For detection of dead plant cells and pathogen structures in infected plant tissues, infected leaves of *P. vulgaris* were incubated for 1 min in boiling lactophenol trypan blue staining solution (2 vol 96% ethanol and 1 vol trypan blue stock solution (10 ml of lactic acid, 10 ml of glycerol, 10 g of phenol, 40 mg trypan blue (Sigma-Aldrich, Germany) dissolved in 10 ml of distilled water). The stained leaves were decolorized by incubating them for several days in chloral hydrate solution (250% of chloral hydrate in distilled water). Then, the leaves were washed with water and transferred into 50% glycerol solution for microscopy. For visualization of the accumulation of ROS in infected plant tissues, leaves were incubated for 2 h in DAB (3,3′-diaminobenzidine) solution (0.5% DAB in 100 mM citric acid, pH 3.7). Then, chlorophyll was extracted by incubating the leaves for 5 min in boiling ethanol. Bright field images of lesions were taken using the Zeiss SteREO Discovery V.20 stereomicroscope or the AxioScope.A1 microscope equipped with an AxioCam MRc camera and the Axiovision Rel 4.8 software package (Zeiss, Germany).

### Fluorescence microscopy

For microscopy, conidia of the *B. cinerea* strains were suspended in Gamborg's B5 solution supplemented with 2% glucose and 0.02% ammonium phosphate, and incubated under humid conditions on microscope slides or on onion epidermal strips. For detection of nuclei, nucleic acids were stained using the fluorescent dye Hoechst 33342 (Sigma-Aldrich, Germany) by adding 10 µl of freshly prepared Hoechst solution [89] to the germinated conidia prior to microscopy. Fluorescence and light microscopy was performed with a Zeiss AxioImager.M1 microscope equipped with the ApoTome.2 technology for optical sectioning with structured illumination. Differential interference microscopy (DIC) was used for bright field images. Hoechst staining was examined using the filter set 49 DAPI shift free (excitation G 365, beam splitter FT 395, emission BP 445/50), and GFP fluorescence using filter set 38 (excitation BP 470/40, beam splitter FT 495, emission BP 525/50). Images (optical sections and Z-stacks) were captured with an AxioCam MRm camera and analyzed using the Axiovision Rel 4.8 software package (Zeiss, Germany).

### Microarrays analyses

To study BcVEL1-dependent gene expression *in planta*, primary leaves of *P. vulgaris* were inoculated with conidial suspensions (2×10^5^ conidia/ml Gamborg's B5+2% glucose) of the wild-type strain B05.10 and the Δ*bcvel1* mutant. Primary lesions (from three plants per experiment; four independent experiments for four biological replicates) were harvested after 48 h of incubation and were subsequently used for RNA isolation (Trizol procedure). Total RNA was treated with the DNA-free kit to remove any trace of DNA (Ambion - Applied Biosystems, France). Synthesis of double-stranded cDNA, Cy3-labeling and hybridization on microarrays were done by PartnerChip (http://www.partnerchip.fr/) using the procedures established by Nimblegen (Roche) and the reagents from Invitrogen (Life technologies, France). To study the complete transcriptome of *B. cinerea*, NimbleGen 4-plex arrays with 62,478 60-mer specific probes covering all the 20,885 predicted gene models and non-mapping ESTs of *B. cinerea* [47] were used. Data processing, quality controls and differential expression analysis were performed using ANAIS methods [90]. Probe hybridization signals were first RMA-background corrected, quantile normalized, and gene-summarized [91], [92]. The differentially expressed genes were then identified using an ANOVA test. Transcripts with a p-value<0.05, a normalized variance >0.4 and more than 2-fold change in transcript level were considered as significantly differentially expressed. Gene Ontology (GO) enrichment analyses were further performed on the selected lists of genes with the GOEAST toolkit [93], to highlight significantly enriched GO terms compared to the complete list of functionally annotated *B. cinerea* genes [47]. Details on the experiments, raw values and lists of differentially expressed genes are available at http://urgi.versailles.inra.fr/Data/Transcriptome.

### Complementation of *Fusarium fujikuroi* Δ*vel1* with *bcvel1*

The hygromycin-resistant mutant strain *F. fujikuroi* Δ*vel1* [32] was transformed with plasmid p*bcvel1*-COM*^bcniaD^* (see above) comprising *bcvel1* and 800 bp and 240 bp of its 5′- and 3′-non-coding regions as well as a nourseothricin resistance cassette. Preparation and transformation of protoplasts from *F. fujikuroi* was carried out as previously described [94]. Random integration of the *bcvel1* construct in nourseothricin-resistant transformants was verified by PCR using primers pCSN44-*trpC*-P and *bcvel1*-COM-R (Table S5). Media for plate assays of *F. fujikuroi* strains (Table 1) were V8 (30 mM CaCO_3_, 20% V8 juice; Campbell Foods, Belgium) and PDA (Potato dextrose agar; Sigma-Aldrich, Germany). For submerged cultures, *F. fujikuroi* was pre-incubated at 28°C and 190 rpm for 48 h in 100 ml of Darken medium [95]. For detection of gibberellin and bikaverin production, 0.5 ml of the pre-culture was used to inoculate 100 ml of ICI media [96] containing 6 mM glutamine (10% ICI medium), and incubated at 28°C and 190 rpm. Extraction and detection of GA_3_ and GA_4/7_ was done according to Wiemann et al. [32] For virulence assays, seeds of *Oryza sativa* spp. *japonica* c.v. Nipponbare were surface sterilized and incubated on solid water agar for 3 d at 4°C in the dark followed by 3 d at 28°C in a 12 h light/12 h dark cycle. Mycelial plugs of 3-d-old *F. fujikuroi* cultures and the germinated seeds were transferred to test tubes filled with vermiculite (Deutsche Vermiculite Dämmstoff GmbH, Germany), and watered with 10 ml of GB5 medium. As a negative control, a plug of the water agar was used instead of a fungal strain, and 100 ppm GA3 were used as a positive control. The test tubes were incubated at 28°C in a 12 h light/12 h dark cycle for 10 days. Rice plants were cleaned from vermiculite and measured from the base of the stem to the second nodule.

## Supporting Information

Figure S1 **Genes located in the identified 115-kb genomic region linked with sclerotia formation.** Annotated proteins from *B. cinerea* T4, B05.10 and *S. sclerotiorum* 1980 are listed. Possible gene functions were assigned due to BlastX results. Deletion mutants of genes encoding the Gα subunit BCG3 and the stress-activated MAP kinase BcSAK1 were previously described. No SNPs were found in these genes.
(TIF)

Figure S2 **Multiple sequence alignment of BcVEL1 with other VeA homologues.** The alignment was generated using ClustalW (http://genius.embnet.dkfz-heidelberg.de/menu/w2h/w2hdkfz/). Sequences aligned are: *B. cinerea* BcVEL1 (HE977589; end of the T4 protein is indicated by a red arrow), *S. sclerotinia* VEL1 (SS1G_07626), *N. crassa* VE-1 (CAB92641), *F. fujikuroi* VEL1 (CBE54373), *A. nidulans* VeA (AAD42946), *A. parasiticus* VeA (AAS07022), and *Histoplasma capsulatum* VEA1 (ACB59235). Amino acids that are identical in all protein sequences are shaded black, amino acids that are identical in six out of the seven protein sequences are shaded gray, and amino acids that are identical in BcVEL1, SsVEL1 and others are shaded green.
(TIF)

Figure S3 **Heterologous complementation in** ***Fusarium fujikuroi*** **.** Phenotypes of the *F. fujikuroi* wild-type strain IMI58289, the *Ffvel1* deletion mutant and three independent transformants expressing *bcvel1* in the Δ*Ffvel1* background are shown. (A) Colony morphologies of strains grown on different media for 10 days in continuous darkness. CM, complete medium; V8, vegetable juice medium; PDA, potato dextrose medium. (B) Numbers of microconidia produced by the different strains grown for 10 days on V8 solid medium in continuous light. (C) Red pigmentation of culture broths due to the accumulation of bikaverin. Strains were grown for in 10% liquid ICI medium at 28°C and 180 rpm. Pictures were taken after 1 and 3 days. (D) Thin layer chromatogram for detection of gibberellic acid (GA) production. Strains were grown for 5 days in 10% ICI medium. GA_4/7_ and GA_3_ were used as standards (for details see Materials and Methods). (E) Virulence assay on rice (*Oryza sativa* L.). Seedlings were infected with agar plugs of the different *F. fujikuroi* strains. Experiments were carried out in triplicates; lengths of seedlings were determined 10 dpi.
(TIF)

Figure S4 **Construction of** ***bcvel1*** **mutants.** (A) Replacement of *bcvel1* by a deletion construct containing a hygromycin resistance cassette (P*trpC*::*hph*) or a complementation construct comprising the *bcvel1^B05.10^* open reading frame and a nourseothricin resistance cassette (P*trpC*::*nat1*). The latter one was transformed into B05.10:Δ*bcvel1* and isolate T4. (B) Diagnostic PCR of the different homokaryotic *bcvel1* deletion mutants. Homologous recombination was detected by PCR using the primer pairs: *bcvel1*-hi5F/*trpC*-T and *trpC*-P/*bcvel1*-hi3R. Wild-type alleles were detected using primers *bcvel1*-WT-F and WT-R (see Fig. S4A). (C) Southern blot analyses of the homokaryotic *bcvel1* deletion mutants. Genomic DNA of the mutants and the recipient strain B05.10 was digested with *Eco*RI and transferred to a nylon membrane. The blot was hybridized with the 3′ flank of *bcvel1*. The hygromycin resistance cassette contains an additional *Eco*RI site resulting in smaller hybridizing fragments in the replacement mutants. (D) Targeted integration of *bcvel1-gfp* constructs by replacement of *bcniiA* encoding the nitrite reductase. *Bcvel1* amplicons were generated using primers *bcvel1*-GFP-F and T4-GFP-R or B05.10-GFP-R (see Fig. S4A) and integrated into expression vector pNAN-OGG. Homologous integration at *bcniiA*-5′ was detected by PCR using primers *bcniiA*-hi5F and T*gluc*-hiF, and at *bcniiA*-3′ using primers *bcniiA*-hi3R and *nat1*-hiF. (E) Detection of *bcvel1* expression levels in the different mutants. Strains were grown for 3 days on complete medium with cellophane overlays. rRNA is shown as loading control. Weak signals were indicated by asterisks.
(TIF)

Figure S5 **Response of Δ** ***bcvel1*** **mutants to oxidative and osmotic stress.** (A) Colony appearance of Δ*bcvel1* and wild type B05.10 on complete medium (CM) without stressors (control) and CM supplemented with 7.5 mM H_2_O_2_ or 500 µM menadione for induction of oxidative stress, and with 1.4 M sorbitol or 0.7 M NaCl for induction of osmotic stress. Strains were incubated for 4 days in continuous light (LL), light-dark (LD) or continuous darkness (DD). (B) Quantification of growth rates of strains in response to oxidative stress (7.5 mM H_2_O_2_ or 500 µM menadione). Mean values of colony diameters were determined from five colonies per strain and condition. (C) Detection of hydrogen peroxide generation by DAB staining. 3-d-old colonies were overlaid with DAB (3,3′-diaminobenzidine) staining solution and incubated for 1 h. Then, DAB solution was discarded; pictures were taken after 14 h.
(TIF)

Figure S6 **Growth and conidiation pattern of** ***bcvel1*** **mutants.** Strains were grown for 7 days on complete medium supplemented with 0.02% SDS that results in comparably reduced daily growth rates of both WT:B05.10 and the *bcvel1* deletion mutant, illustrating the different conidiation pattern in response to the 12 h light/12 h dark rhythm. Daily growth rates are: WT:B05.10 on CM – 13.0 mm/d, on CM+0.02% SDS – 7.1 mm/d; Δ*bcvel1* on CM – 12.7 mm/d, on CM+0.02% SDS – 7.9 mm/d.
(TIF)

Figure S7 **BcVEL1 affects the formation of the dark pigment melanin.** (A) Melanin formation in liquid cultures. Strains were grown for 48 h in liquid medium at 20°C, 150 rpm, in light-dark (LD) or continuous darkness (DD), with or without tricyclazole (10 µg/ml) representing a specific reductase inhibitor. Due the inhibition of the enzymes involved in later stages of the melanin biosynthetic pathway, the polyketide 1,3,6,8-THN formed by the key enzyme BcPKS13 is converted to the orange pigment flaviolin. (B) Expression of genes involved in the melanin biosynthetic pathway in strains that were grown for 48 h in liquid medium in light-dark (LD) or continuous darkness (DD). BcPKS13, 1,3,6,8-tetrahydroxynaphthalene synthase; BcSCD1, scytalone dehydratase; BcBRN1, 1,3,8,-trihydroxynaphthalene (THN) reductase. rRNA is shown as loading control.
(TIF)

Figure S8 **Virulence assays on** ***P. vulgaris*** **using non-sporulating mycelia for inoculation.** Strains were grown for 2 d on solid CM medium and then equal plugs of the non-sporulating mycelia were put on the leaves. Diameters of eight lesions per strain were determined after 2 days of incubation.
(TIF)

Figure S9 **Gene Ontology enrichment analyses of the differentially expressed genes in WT:B05.10 and Δbcvel1 using the GOEAST tool.** (A) Enrichment analyses of the 227 under-expressed genes in Δ*bcvel1*; GOEAST enrichment based on 109 genes with GO. (B) Enrichment analyses of the 419 over-expressed genes in Δbcvel1; GOEAST enrichment based on 216 genes with GO. The web-based toolkit identifies statistically overrepresented GO terms within given gene sets. Black boxes represent GO terms. Significantly enriched GO terms are marked yellow. The degree of color saturation of each node is positively correlated with the enrichment significance of the corresponding GO term. Non-significant GO terms within the hierarchical tree are shown as white boxes. Branches of the GO hierarchical tree without significantly enriched GO terms are not shown. Arrows represent connections between different GO terms. Red arrows represent relationships between two enriched GO terms, black solid arrows represent relationships between enriched and un-enriched terms and black dashed arrows represent relationships between two un-enriched GO terms. Array gene-IDs for the enriched GO terms are indicated in red boxes.
(TIF)

Figure S10 **Sensitivity to chlorate as indication for nitrate reductase activity.** Strains were grown on solid complete medium (CM) without and with 0.4 M KClO_3_, respectively. Diameters of six colonies per strain and condition were measured after 3 days of incubation in light-dark conditions. Increased sensitivity to chlorate indicates increased nitrate reductase activity, as the toxic effect of chlorate is based on its conversion to chlorite by the nitrate reductase. Consequently, mutants of *bcniaD* encoding the nitrate reductase are insensitive to chlorate, while deletion mutants of *bcniiA* encoding the nitrite reductase are exhibiting wild type-like sensitivity to chlorate.
(TIF)

Figure S11 **Identification of a BcVEL1-dependent gene cluster.** (A) Expression data derived from the microarray experiment for predicted genes on Bt4_SuperContig_144_1 (BofuT4_T091900 to BofuT4_T092110). Shown are the mean values and standard deviations of the normalized intensities of the four biological replicates. (B) Comparison of genomic regions from *B. cinerea* and *S. sclerotiorum*. Genes indicated as red arrows are over-expressed in the Δ*bcvel1* mutant and are missing in the genome of *S. sclerotiorum*. For more details on putative functions of the gene see Table S3.
(TIF)

Table S1 **List of the 227 under-expressed genes in Δ** ***bcvel1*** **mutant.**
(DOCX)

Table S2 **List of the 419 over-expressed genes in Δ** ***bcvel1*** **mutant.**
(DOCX)

Table S3 **Identification of a BcVEL1-dependent gene cluster.**
(DOCX)

Table S4 **Primers used to amplify the microsatellites markers on strains T4, 32 and their progeny.**
(DOCX)

Table S5 **Primers used for sequencing and mutant construction.**
(DOCX)
