# Supplementary figures and images for: Natural Variation in the VELVET Gene bcvel1 Affects Virulence and Light-Dependent Differentiation in Botrytis cinerea
Source: PLoS One. 2012 Oct 31;7(10):e47840. doi: 10.1371/journal.pone.0047840 (PMC3485325; doi:10.1371/journal.pone.0047840)

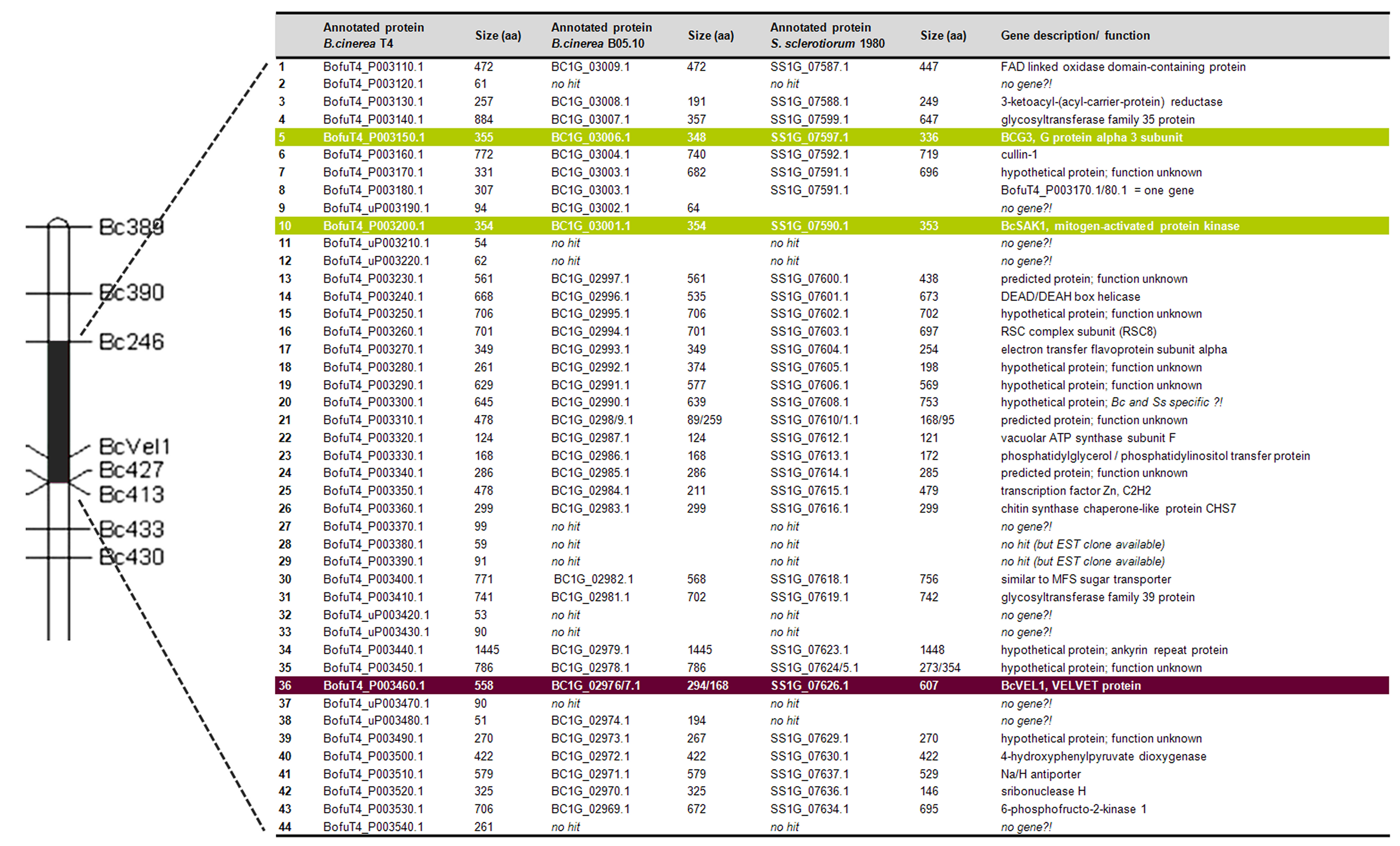

Supplement: Figure S1 — Genes located in the identified 115-kb genomic region linked with sclerotia formation. Annotated proteins from B. cinerea T4, B05.10 and S. sclerotiorum 1980 are listed. Possible gene functions were assigned due to BlastX results. Deletion mutants of genes encoding the Gα subunit BCG3 and the stress-activated MAP kinase BcSAK1 were previously described. No SNPs were found in these genes. (TIF) [file pone.0047840.s001.tif]

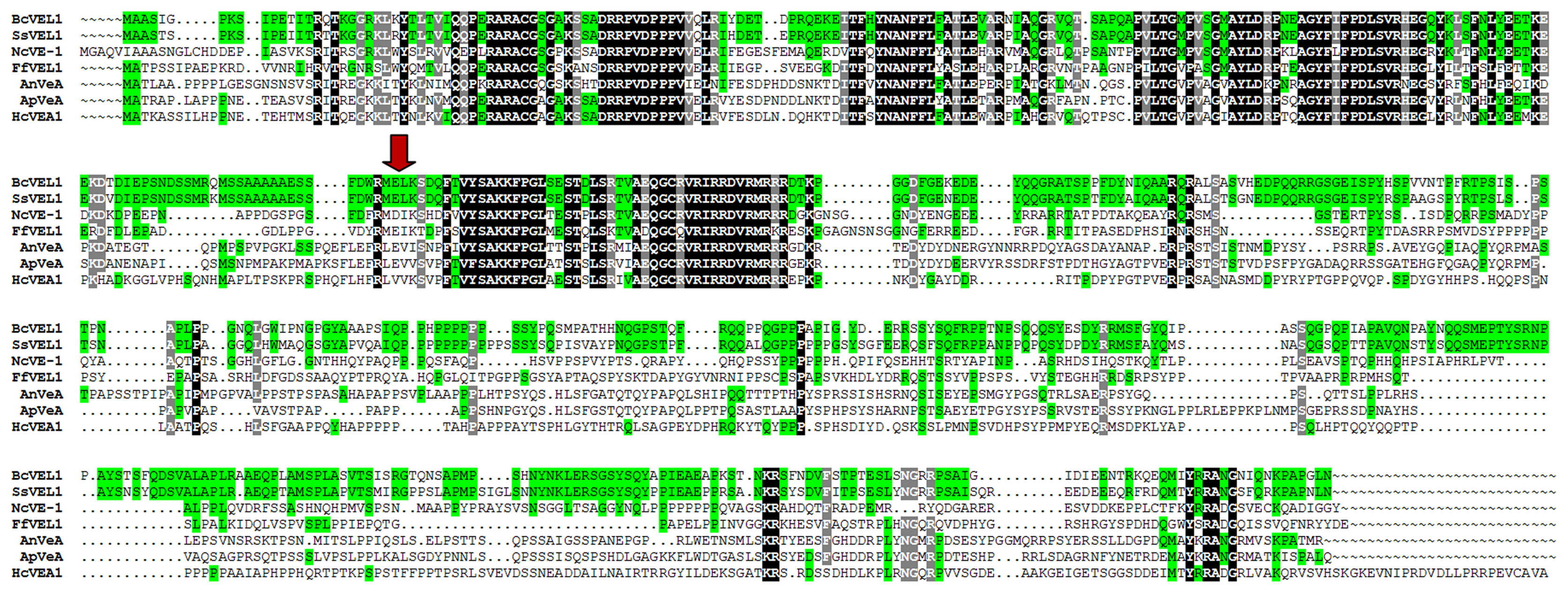

Supplement: Figure S2 — Multiple sequence alignment of BcVEL1 with other VeA homologues. The alignment was generated using ClustalW (http://genius.embnet.dkfz-heidelberg.de/menu/w2h/w2hdkfz/). Sequences aligned are: B. cinerea BcVEL1 (HE977589; end of the T4 protein is indicated by a red arrow), S. sclerotinia VEL1 (SS1G_07626), N. crassa VE-1 (CAB92641), F. fujikuroi VEL1 (CBE54373), A. nidulans VeA (AAD42946), A. parasiticus VeA (AAS07022), and Histoplasma capsulatum VEA1 (ACB59235). Amino acids that are identical in all protein sequences are shaded black, amino acids that are identical in six out of the seven protein sequences are shaded gray, and amino acids that are identical in BcVEL1, SsVEL1 and others are shaded green. (TIF) [file pone.0047840.s002.tif]

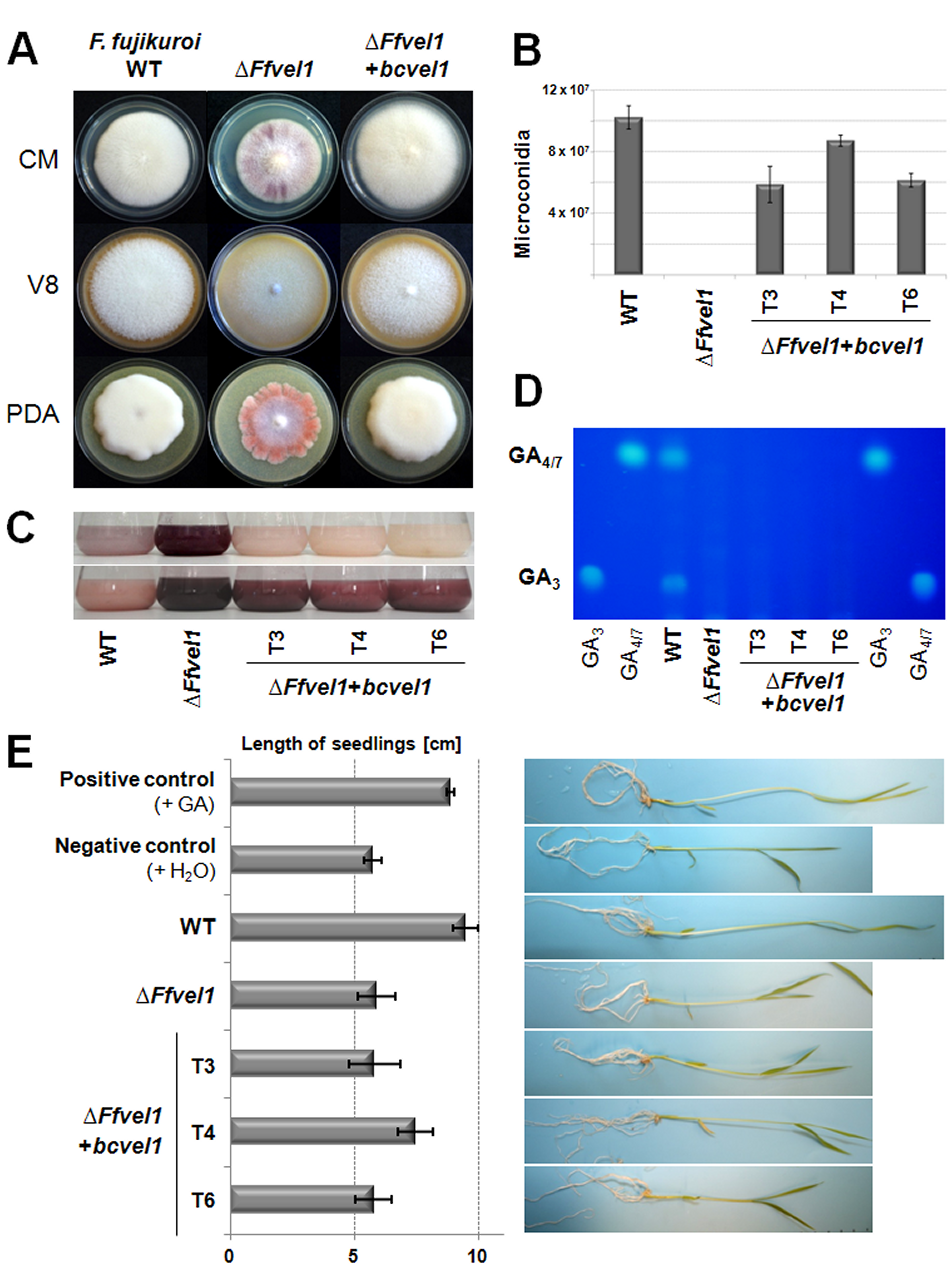

Supplement: Figure S3 — Heterologous complementation in Fusarium fujikuroi . Phenotypes of the F. fujikuroi wild-type strain IMI58289, the Ffvel1 deletion mutant and three independent transformants expressing bcvel1 in the ΔFfvel1 background are shown. (A) Colony morphologies of strains grown on different media for 10 days in continuous darkness. CM, complete medium; V8, vegetable juice medium; PDA, potato dextrose medium. (B) Numbers of microconidia produced by the different strains grown for 10 days on V8 solid medium in continuous light. (C) Red pigmentation of culture broths due to the accumulation of bikaverin. Strains were grown for in 10% liquid ICI medium at 28°C and 180 rpm. Pictures were taken after 1 and 3 days. (D) Thin layer chromatogram for detection of gibberellic acid (GA) production. Strains were grown for 5 days in 10% ICI medium. GA4/7 and GA3 were used as standards (for details see Materials and Methods). (E) Virulence assay on rice (Oryza sativa L.). Seedlings were infected with agar plugs of the different F. fujikuroi strains. Experiments were carried out in triplicates; lengths of seedlings were determined 10 dpi. (TIF) [file pone.0047840.s003.tif]

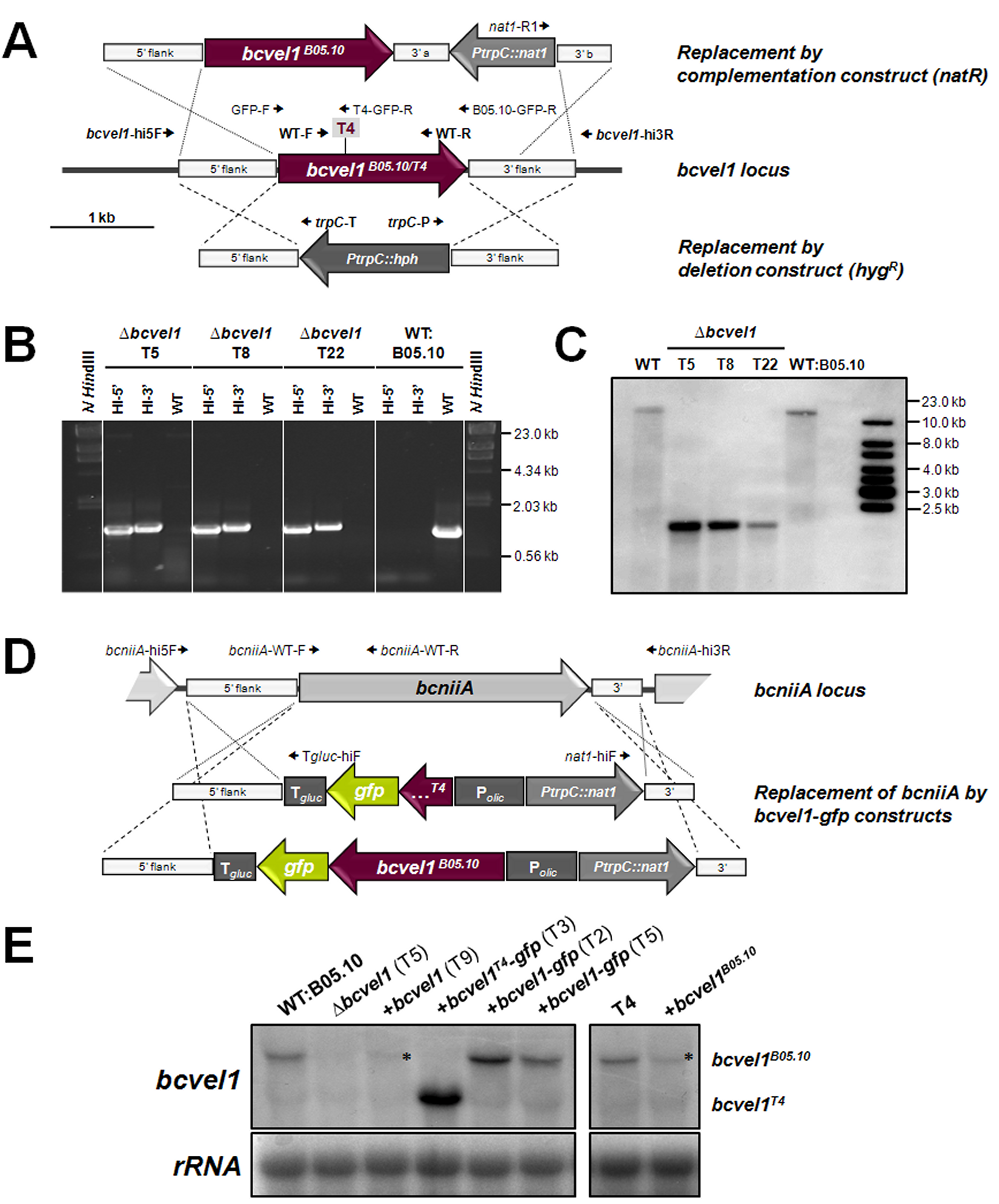

Supplement: Figure S4 — Construction of bcvel1 mutants. (A) Replacement of bcvel1 by a deletion construct containing a hygromycin resistance cassette (PtrpC::hph) or a complementation construct comprising the bcvel1B05.10 open reading frame and a nourseothricin resistance cassette (PtrpC::nat1). The latter one was transformed into B05.10:Δbcvel1 and isolate T4. (B) Diagnostic PCR of the different homokaryotic bcvel1 deletion mutants. Homologous recombination was detected by PCR using the primer pairs: bcvel1-hi5F/trpC-T and trpC-P/bcvel1-hi3R. Wild-type alleles were detected using primers bcvel1-WT-F and WT-R (see Fig. S4A). (C) Southern blot analyses of the homokaryotic bcvel1 deletion mutants. Genomic DNA of the mutants and the recipient strain B05.10 was digested with EcoRI and transferred to a nylon membrane. The blot was hybridized with the 3′ flank of bcvel1. The hygromycin resistance cassette contains an additional EcoRI site resulting in smaller hybridizing fragments in the replacement mutants. (D) Targeted integration of bcvel1-gfp constructs by replacement of bcniiA encoding the nitrite reductase. Bcvel1 amplicons were generated using primers bcvel1-GFP-F and T4-GFP-R or B05.10-GFP-R (see Fig. S4A) and integrated into expression vector pNAN-OGG. Homologous integration at bcniiA-5′ was detected by PCR using primers bcniiA-hi5F and Tgluc-hiF, and at bcniiA-3′ using primers bcniiA-hi3R and nat1-hiF. (E) Detection of bcvel1 expression levels in the different mutants. Strains were grown for 3 days on complete medium with cellophane overlays. rRNA is shown as loading control. Weak signals were indicated by asterisks. (TIF) [file pone.0047840.s004.tif]

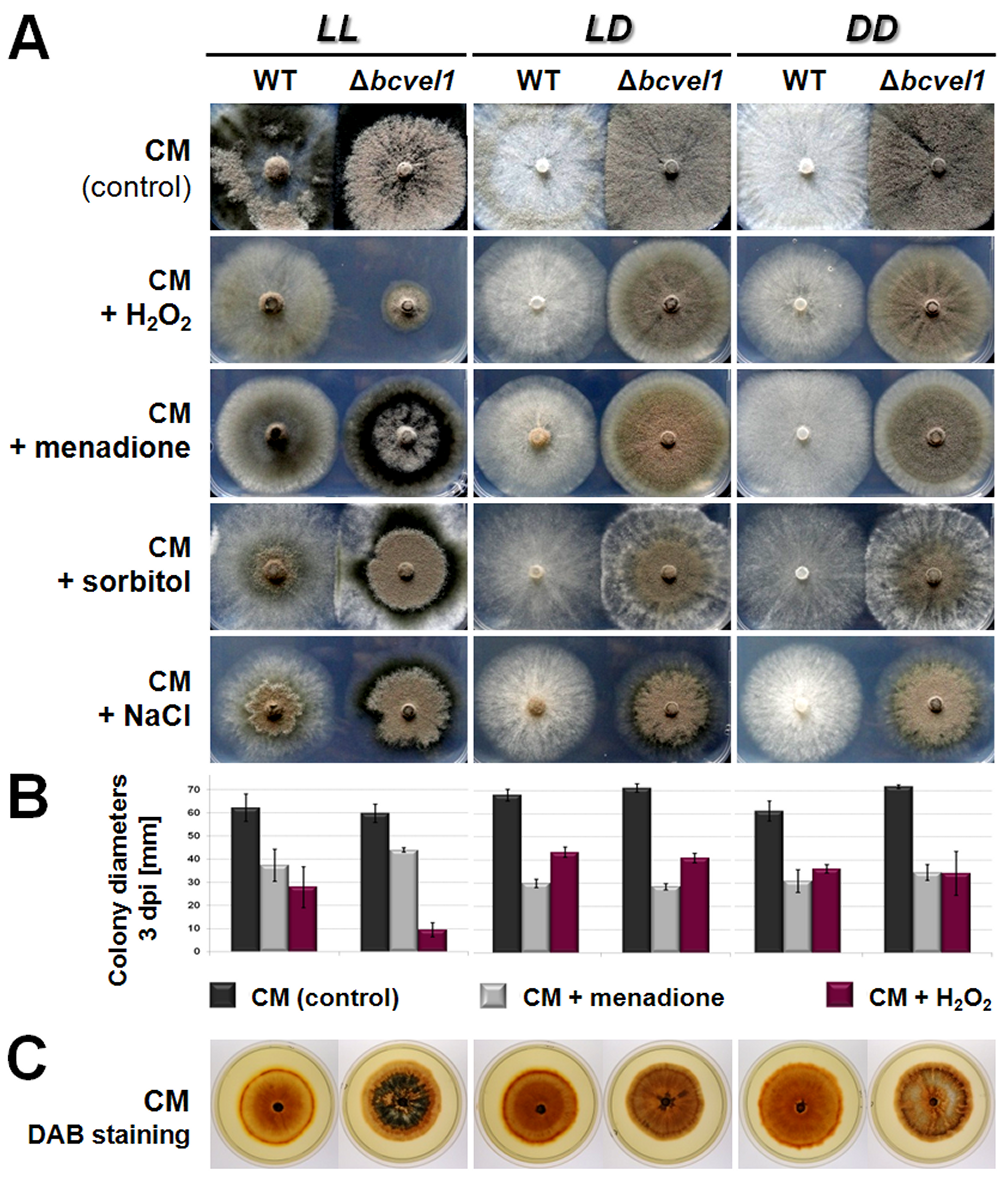

Supplement: Figure S5 — Response of Δ bcvel1 mutants to oxidative and osmotic stress. (A) Colony appearance of Δbcvel1 and wild type B05.10 on complete medium (CM) without stressors (control) and CM supplemented with 7.5 mM H2O2 or 500 µM menadione for induction of oxidative stress, and with 1.4 M sorbitol or 0.7 M NaCl for induction of osmotic stress. Strains were incubated for 4 days in continuous light (LL), light-dark (LD) or continuous darkness (DD). (B) Quantification of growth rates of strains in response to oxidative stress (7.5 mM H2O2 or 500 µM menadione). Mean values of colony diameters were determined from five colonies per strain and condition. (C) Detection of hydrogen peroxide generation by DAB staining. 3-d-old colonies were overlaid with DAB (3,3′-diaminobenzidine) staining solution and incubated for 1 h. Then, DAB solution was discarded; pictures were taken after 14 h. (TIF) [file pone.0047840.s005.tif]

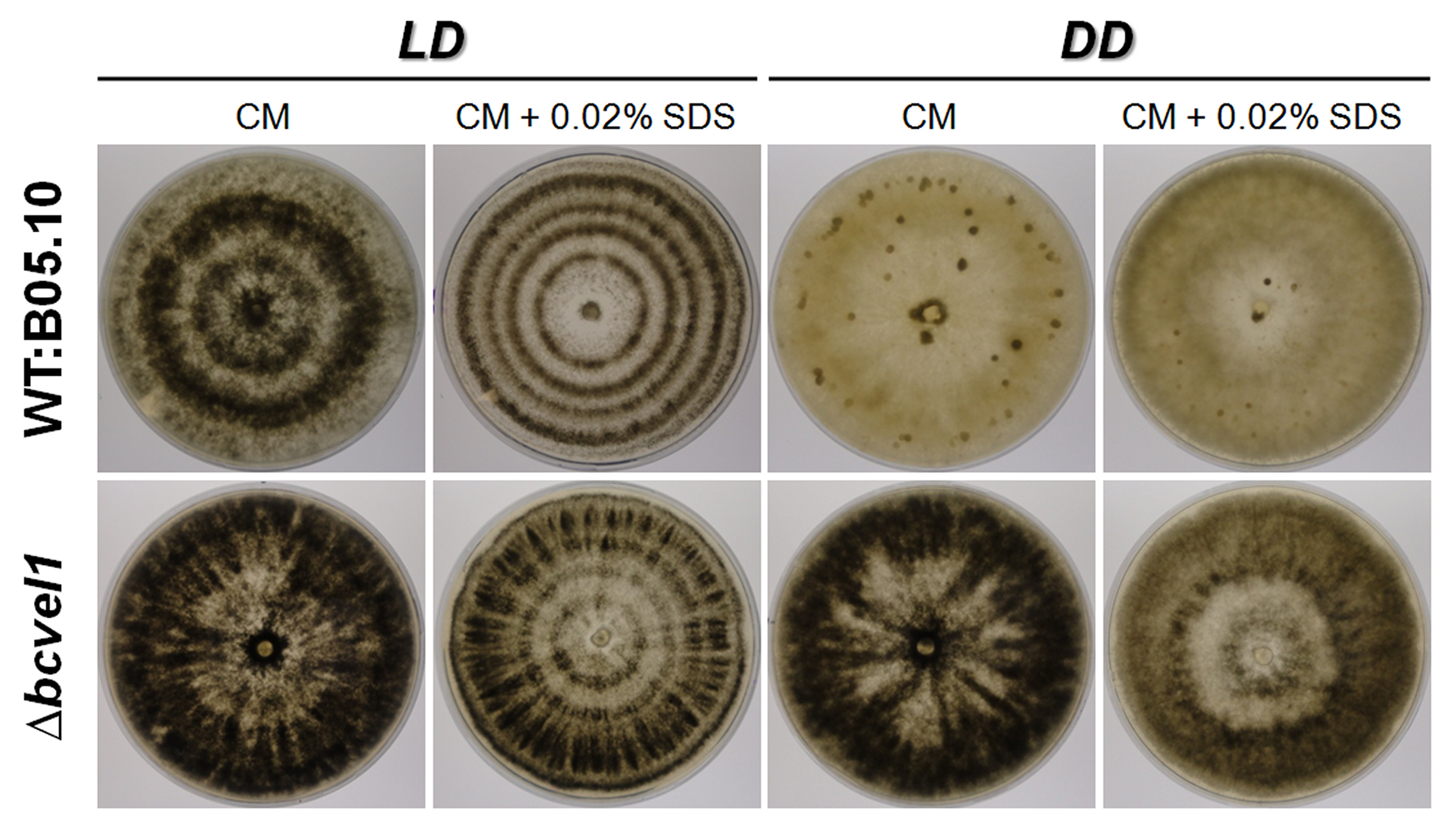

Supplement: Figure S6 — Growth and conidiation pattern of bcvel1 mutants. Strains were grown for 7 days on complete medium supplemented with 0.02% SDS that results in comparably reduced daily growth rates of both WT:B05.10 and the bcvel1 deletion mutant, illustrating the different conidiation pattern in response to the 12 h light/12 h dark rhythm. Daily growth rates are: WT:B05.10 on CM – 13.0 mm/d, on CM+0.02% SDS – 7.1 mm/d; Δbcvel1 on CM – 12.7 mm/d, on CM+0.02% SDS – 7.9 mm/d. (TIF) [file pone.0047840.s006.tif]

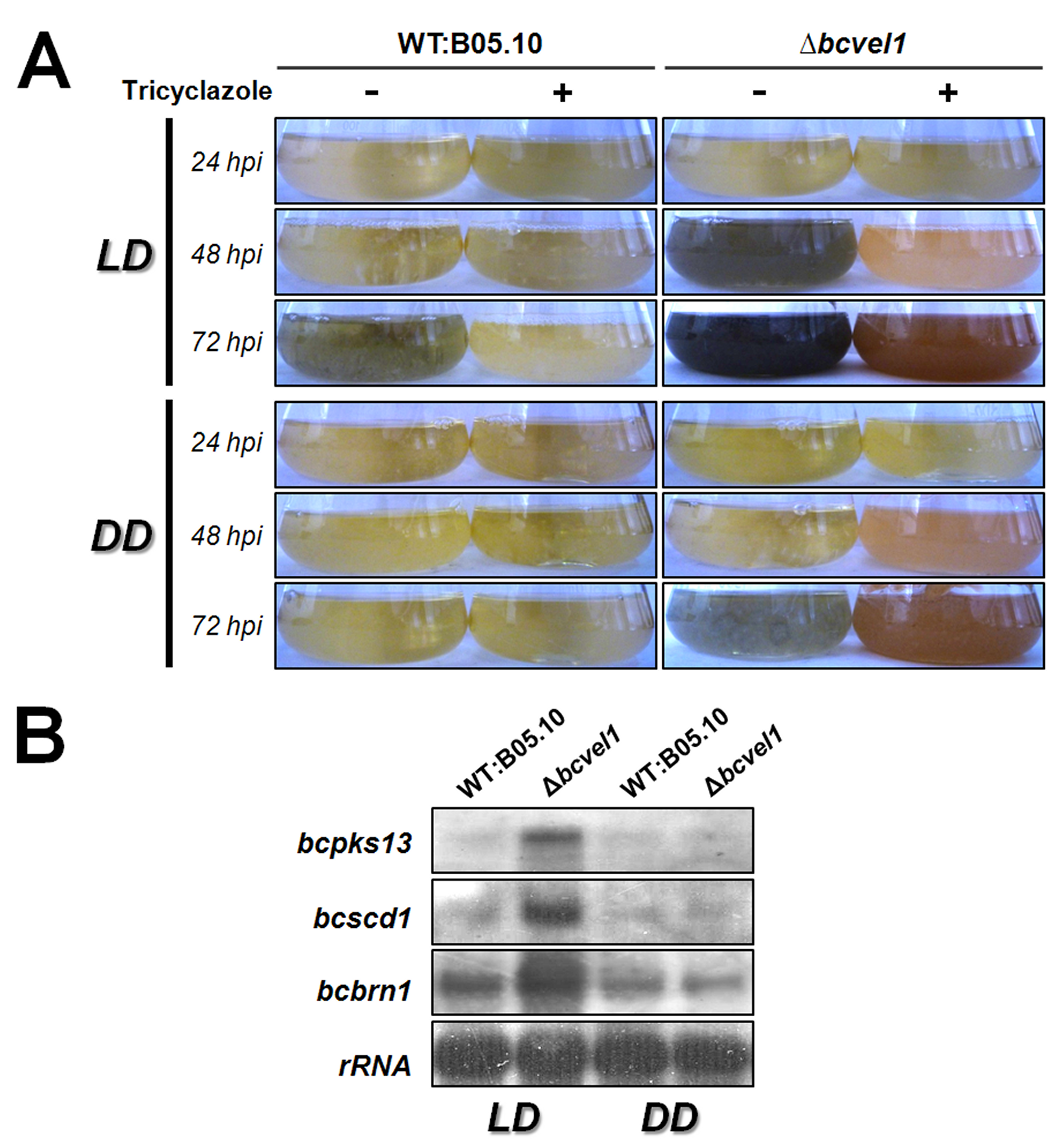

Supplement: Figure S7 — BcVEL1 affects the formation of the dark pigment melanin. (A) Melanin formation in liquid cultures. Strains were grown for 48 h in liquid medium at 20°C, 150 rpm, in light-dark (LD) or continuous darkness (DD), with or without tricyclazole (10 µg/ml) representing a specific reductase inhibitor. Due the inhibition of the enzymes involved in later stages of the melanin biosynthetic pathway, the polyketide 1,3,6,8-THN formed by the key enzyme BcPKS13 is converted to the orange pigment flaviolin. (B) Expression of genes involved in the melanin biosynthetic pathway in strains that were grown for 48 h in liquid medium in light-dark (LD) or continuous darkness (DD). BcPKS13, 1,3,6,8-tetrahydroxynaphthalene synthase; BcSCD1, scytalone dehydratase; BcBRN1, 1,3,8,-trihydroxynaphthalene (THN) reductase. rRNA is shown as loading control. (TIF) [file pone.0047840.s007.tif]

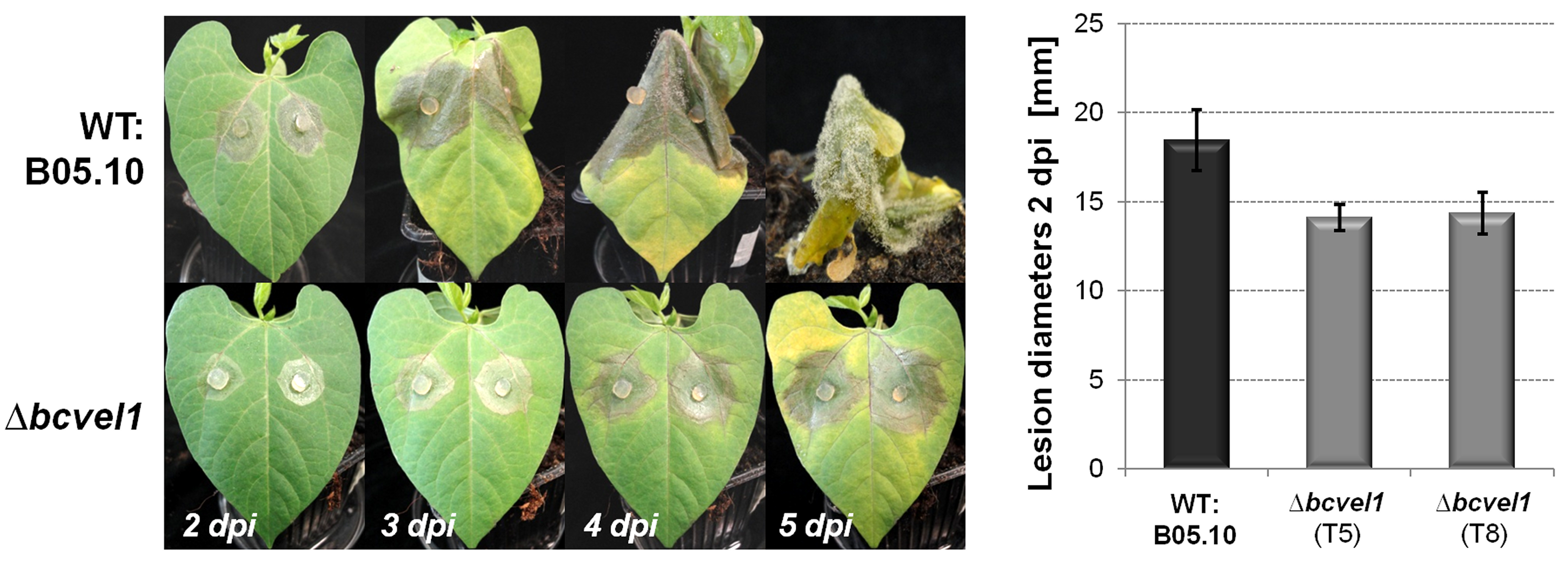

Supplement: Figure S8 — Virulence assays on P. vulgaris using non-sporulating mycelia for inoculation. Strains were grown for 2 d on solid CM medium and then equal plugs of the non-sporulating mycelia were put on the leaves. Diameters of eight lesions per strain were determined after 2 days of incubation. (TIF) [file pone.0047840.s008.tif]

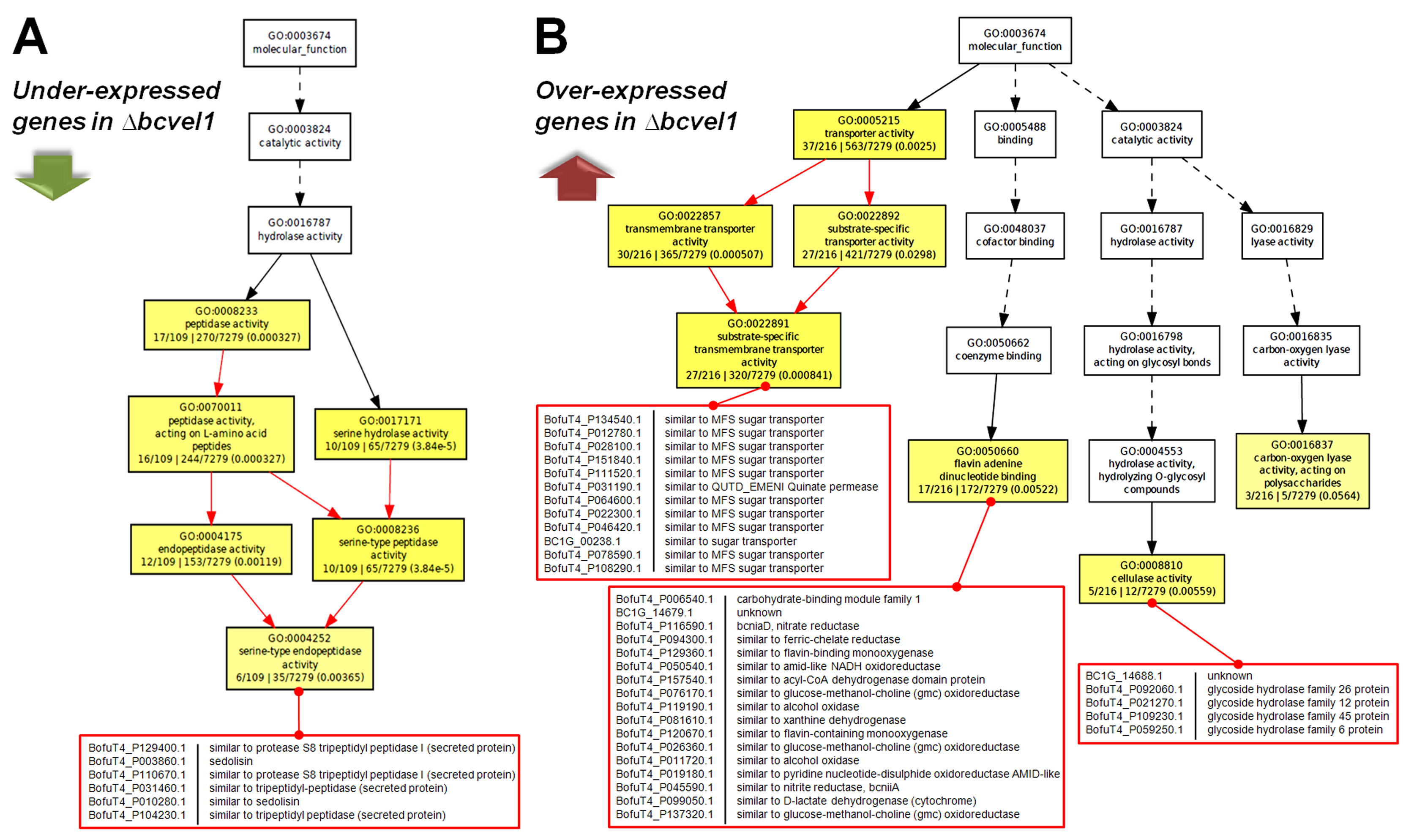

Supplement: Figure S9 — Gene Ontology enrichment analyses of the differentially expressed genes in WT:B05.10 and Δbcvel1 using the GOEAST tool. (A) Enrichment analyses of the 227 under-expressed genes in Δbcvel1; GOEAST enrichment based on 109 genes with GO. (B) Enrichment analyses of the 419 over-expressed genes in Δbcvel1; GOEAST enrichment based on 216 genes with GO. The web-based toolkit identifies statistically overrepresented GO terms within given gene sets. Black boxes represent GO terms. Significantly enriched GO terms are marked yellow. The degree of color saturation of each node is positively correlated with the enrichment significance of the corresponding GO term. Non-significant GO terms within the hierarchical tree are shown as white boxes. Branches of the GO hierarchical tree without significantly enriched GO terms are not shown. Arrows represent connections between different GO terms. Red arrows represent relationships between two enriched GO terms, black solid arrows represent relationships between enriched and un-enriched terms and black dashed arrows represent relationships between two un-enriched GO terms. Array gene-IDs for the enriched GO terms are indicated in red boxes. (TIF) [file pone.0047840.s009.tif]

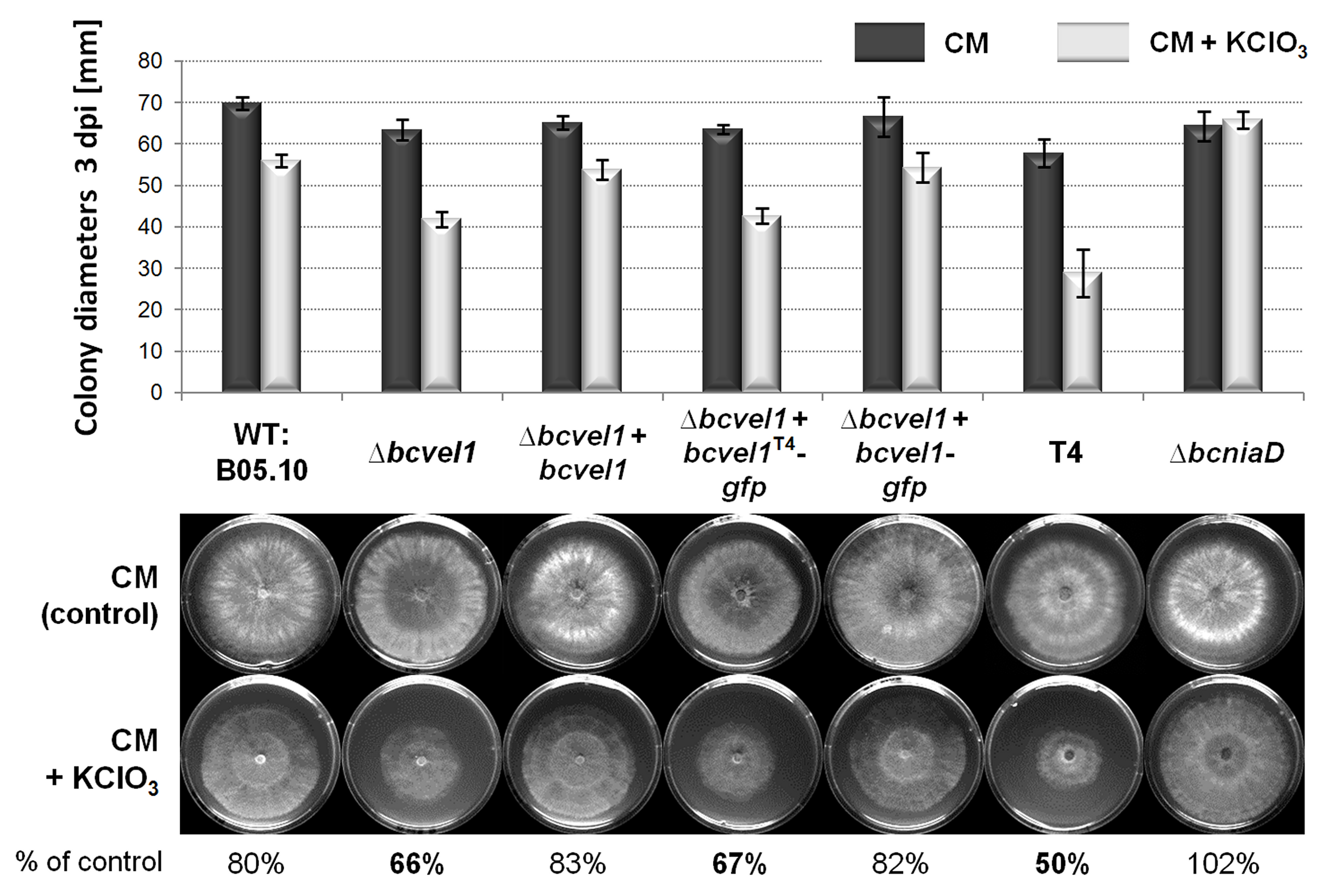

Supplement: Figure S10 — Sensitivity to chlorate as indication for nitrate reductase activity. Strains were grown on solid complete medium (CM) without and with 0.4 M KClO3, respectively. Diameters of six colonies per strain and condition were measured after 3 days of incubation in light-dark conditions. Increased sensitivity to chlorate indicates increased nitrate reductase activity, as the toxic effect of chlorate is based on its conversion to chlorite by the nitrate reductase. Consequently, mutants of bcniaD encoding the nitrate reductase are insensitive to chlorate, while deletion mutants of bcniiA encoding the nitrite reductase are exhibiting wild type-like sensitivity to chlorate. (TIF) [file pone.0047840.s010.tif]

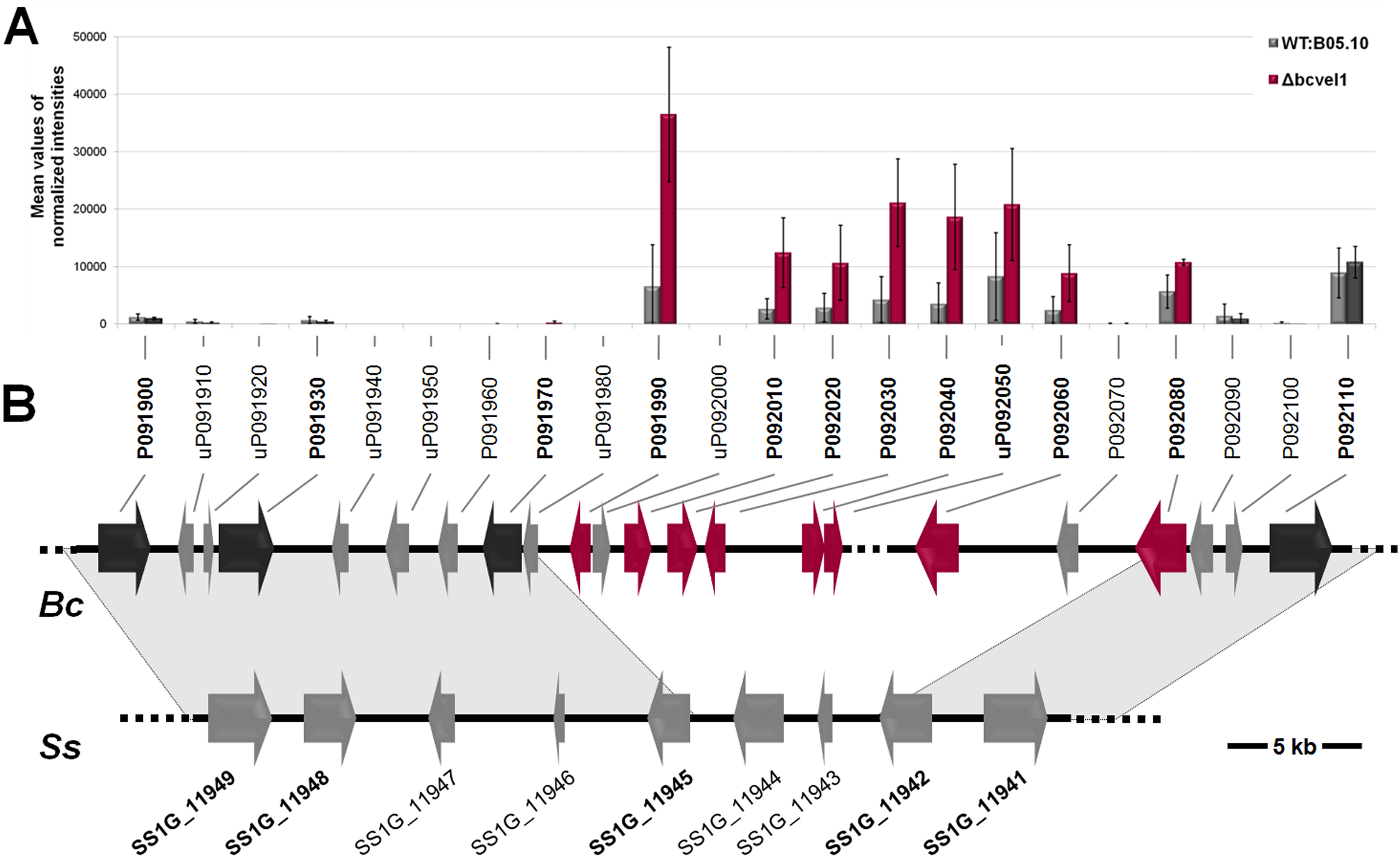

Supplement: Figure S11 — Identification of a BcVEL1-dependent gene cluster. (A) Expression data derived from the microarray experiment for predicted genes on Bt4_SuperContig_144_1 (BofuT4_T091900 to BofuT4_T092110). Shown are the mean values and standard deviations of the normalized intensities of the four biological replicates. (B) Comparison of genomic regions from B. cinerea and S. sclerotiorum. Genes indicated as red arrows are over-expressed in the Δbcvel1 mutant and are missing in the genome of S. sclerotiorum. For more details on putative functions of the gene see Table S3. (TIF) [file pone.0047840.s011.tif]
