# Supplementary material for: Natural Variation in the VELVET Gene bcvel1 Affects Virulence and Light-Dependent Differentiation in Botrytis cinerea
Source: PLoS One. 2012 Oct 31;7(10):e47840. doi: 10.1371/journal.pone.0047840 (PMC3485325; doi:10.1371/journal.pone.0047840)
Supplement: Table S1 — List of the 227 under-expressed genes in Δ bcvel1 mutant. (DOCX) [file pone.0047840.s012.docx]

**Table S1.** List of the 227 under-expressed genes in ∆*bcvel1* mutant.

| **SEQ_ID** | **Broad annotation** | **Function** | **ANOVA**  **p-value** | **ANOVA Normalized Variance** | **FoldChange∆bcvel1_DIV_BY_WT** |
| --- | --- | --- | --- | --- | --- |
| BofuT4_P146330.1 | BC1G_05880 | similar to alpha-ketoglutarate-dependent taurine dioxygenase | 1.60E-03 | 0.96 | -378.57 |
| orphansnoplantaContig114.v4 | | unknown | 4.04E-03 | 0.92 | -282.90 |
| **BofuT4_P004950.1*** | **BC1G_14153** | **BcACP1 (Rolland et al. 2009) (SSH26; Schulze Gronover et al. 2004)** | 1.24E-03 | 0.96 | -209.76 |
| **BofuT4_P003460.1*** | **BC1G_02976** | **BcVEL1** | 1.10E-03 | 0.95 | -135.54 |
| BC1G_15700.1 | BC1G_15700 | similar to efflux pump antibiotic resistance | 5.52E-03 | 0.85 | -121.61 |
| CL_bt4exctg_0529_001.Contig1_v5 | | unknown | 4.08E-02 | 0.51 | -102.47 |
| BC1G_16236.1 | BC1G_16236 | hypothetical protein BC1G_16236 [Botryotinia fuckeliana B05.10] | 1.05E-02 | 0.83 | -85.28 |
| BC1G_16385.1 | BC1G_16385 | unknown | 1.93E-02 | 0.84 | -76.19 |
| BofuT4_P119560.1 | BC1G_14591 | similar to carboxypeptidase S1 (secreted protein) | 1.21E-03 | 0.96 | -63.18 |
| BofuT4_P151510.1 | BC1G_12321 | BcPIC3. gene with maximal expression at complete colonization (Gioti et al. 2006) | 1.14E-03 | 0.91 | -61.66 |
| BofuT4_P153440.1 | BC1G_11386 | similar to MFS multidrug transporter | 3.66E-02 | 0.59 | -50.94 |
| PD0ACA7YA17FM1 |  | unknown | 4.59E-02 | 0.66 | -42.15 |
| B5BC_116_280_E06 |  | unknown | 7.33E-03 | 0.72 | -37.90 |
| BofuT4_P071060.1 | BC1G_15702 | BcPKS7. polyketide synthase. partial sequence | 4.56E-02 | 0.66 | -33.17 |
| BofuT4_P111000.1 | BC1G_05726 | hypothetical protein | 5.11E-03 | 0.59 | -30.51 |
| BC1G_16237.1 | BC1G_16237 | unknown | 3.31E-02 | 0.76 | -26.74 |
| BofuT4_P071120.1 | BC1G_15704 | BcPKS7. polyketide synthase. partial sequence | 8.98E-03 | 0.80 | -23.78 |
| BofuT4_P013080.1 | BC1G_09861 | similar to glutamate decarboxylase | 1.73E-02 | 0.75 | -22.96 |
| BofuT4_P153390.1 | BC1G_11391 | hypothetical protein | 9.56E-03 | 0.72 | -22.15 |
| BofuT4_P162010.1 | BC1G_10524 | similar to MFS multidrug transporter | 1.39E-02 | 0.68 | -21.77 |
| BofuT4_P070940.1 |  | similar to tyrosinase central domain protein | 6.26E-03 | 0.93 | -20.64 |
| BofuT4_P140130.1 | BC1G_14432 | similar to MFS sugar transporter | 2.89E-03 | 0.87 | -20.44 |
| BC1G_09280.1 | BC1G_09280 | unknown | 7.12E-03 | 0.74 | -20.44 |
| BofuT4_P129400.1 | BC1G_14820 | similar to protease S8 tripeptidyl peptidase I (secreted protein) | 2.30E-03 | 0.85 | -18.32 |
| BofuT4_P018370.1 | BC1G_00896 | hypothetical protein | 2.77E-03 | 0.91 | -18.16 |
| BofuT4_P071050.1 | BC1G_15701 | hypothetical protein | 2.53E-04 | 0.94 | -18.05 |
| **BofuT4_P028820.1*** | **BC1G_01286** | **similar to serine carboxypeptidase (CpdS) (secreted protein)** | 4.11E-04 | 0.98 | -17.79 |
| BC1G_16341.1 | BC1G_16341 | unknown | 7.64E-03 | 0.78 | -17.28 |
| BofuT4_P153400.1 | BC1G_11390 | similar to AMP-binding enzyme | 3.50E-02 | 0.68 | -16.27 |
| PD0AGA8YF16CM1 |  | unknown | 2.84E-03 | 0.79 | -16.26 |
| BC1G_16163.1 | BC1G_16163 | similar to aminotransferase | 5.43E-03 | 0.72 | -15.89 |
| BofuT4_P140110.1 | BC1G_14433 | hypothetical protein | 1.31E-02 | 0.81 | -15.62 |
| BofuT4_P060350.1 | BC1G_11081 | similar to cytochrome P450 monooxygenase | 1.71E-03 | 0.47 | -15.52 |
| BofuT4_P003860.1 | BC1G_02944 | sedolisin | 5.14E-03 | 0.79 | -15.37 |
| BofuT4_P111490.1 | BC1G_05677 | similar to OPT oligopeptide transporter | 1.05E-02 | 0.90 | -15.36 |
| BofuT4_P110670.1 | BC1G_05765 | similar to protease S8 tripeptidyl peptidase I (secreted protein) | 9.12E-03 | 0.89 | -14.12 |
| BofuT4_P049400.1 | BC1G_00080 | similar to methyltransferase | 7.31E-03 | 0.79 | -13.90 |
| BofuT4_uP010310.1 | BC1G_08357 | hypothetical protein | 4.94E-02 | 0.68 | -13.74 |
| BofuT4_P002040.1 | BC1G_07459 | similar to isoaspartyl dipeptidase | 4.16E-02 | 0.41 | -13.69 |
| BC1G_14544.1 | BC1G_14544 | similar to cytochrome p450 monooxygenase | 7.49E-03 | 0.86 | -12.84 |
| BofuT4_P111660.1 | BC1G_05658 | hypothetical protein | 1.62E-02 | 0.65 | -12.18 |
| BofuT4_P058130.1 | BC1G_08553 | Bclcc2. laccase | 6.46E-03 | 0.64 | -10.91 |
| BofuT4_P123230.1 | BC1G_08640 | similar to amino acid transporter | 1.34E-02 | 0.81 | -10.72 |
| BofuT4_P151520.1 | BC1G_12320 | hypothetical protein | 3.52E-03 | 0.78 | -10.66 |
| BofuT4_P132640.1 | BC1G_03489 | hypothetical protein | 4.71E-02 | 0.57 | -10.63 |
| BofuT4_P146310.1 | BC1G_05878 | similar to MFS multidrug transporter | 1.83E-03 | 0.79 | -10.57 |
| BC1G_00574.1 | BC1G_00574 | unknown | 3.25E-03 | 0.76 | -10.19 |
| BofuT4_P115140.1 | BC1G_07868 | similar to MFS transporter | 6.15E-03 | 0.55 | -9.90 |
| BofuT4_P153460.1 | BC1G_11384 | hypothetical protein | 4.00E-02 | 0.67 | -9.86 |
| CL_bt4exctg_0530_001.Contig1_v5 | | unknown | 1.44E-02 | 0.63 | -9.46 |
| BofuT4_P069790.1 | BC1G_05216 | similar to delta-12 fatty acid desaturase | 4.79E-03 | 0.86 | -9.32 |
| BofuT4_P153420.1 | BC1G_11387 | similar to delta(12) fatty acid desaturase | 4.74E-02 | 0.68 | -9.31 |
| BC1G_16009.1 | BC1G_16009 | asimilar to minotransferase - | 2.02E-02 | 0.68 | -9.20 |
| BofuT4_P063540.1 | BC1G_13062 | similar to phosphatidylethanolamine-binding protein | 2.24E-02 | 0.55 | -9.04 |
| BofuT4_P103240.1 | BC1G_13136 | hypothetical protein | 3.46E-02 | 0.59 | -8.92 |
| BC1G_02441.1 | BC1G_02441 | similar to iron transport multicopper oxidase fet3 precursor | 2.76E-02 | 0.61 | -8.67 |
| **BofuT4_P094200.1*** | **BC1G_04246** | **BcPG3. endopolygalacturonase 3** | 1.16E-03 | 0.95 | -8.58 |
| BofuT4_P061290.1 | BC1G_02060 | hypothetical protein | 6.33E-03 | 0.61 | -8.57 |
| CL_bt4ctg_0232_005.Contig4_v5 | | unknown | 6.68E-03 | 0.69 | -8.25 |
| BofuT4_P057020.1 | BC1G_10089 | hypothetical protein | 4.73E-02 | 0.40 | -8.15 |
| **BofuT4_P134040.1*** | **BC1G_03070** | **BcAP8. aspartic protease 8 (Ten Have et al. 2010) (SSH22; Schulze Gronover et al. 2004)** | 6.24E-03 | 0.86 | -7.87 |
| BofuT4_P082510.1 |  | hypothetical protein | 3.27E-02 | 0.54 | -7.68 |
| BofuT4_P158240.1 |  | hypothetical protein | 4.06E-02 | 0.69 | -7.57 |
| BofuT4_P020340.1 | BC1G_00689 | hypothetical protein | 4.31E-03 | 0.72 | -7.38 |
| BofuT4_P103200.1 | BC1G_13133 | similar to FAD binding domain-containing protein | 4.50E-03 | 0.78 | -7.14 |
| BofuT4_uP042740.1 |  | predicted protein | 4.11E-02 | 0.64 | -7.12 |
| BC1G_16162.1 | BC1G_16162 | unknown | 4.02E-02 | 0.76 | -7.06 |
| BofuT4_P148710.1 | BC1G_09429 | similar to amino acid transporter | 5.85E-06 | 0.84 | -6.97 |
| BofuT4_uP030250.1 | BC1G_01141 | hypothetical protein | 6.71E-03 | 0.73 | -6.90 |
| BC1G_11935.1 | BC1G_11935 | unknown | 4.96E-02 | 0.56 | -6.83 |
| BofuT4_P003570.1 | BC1G_02965 | hypothetical protein | 1.22E-02 | 0.67 | -6.76 |
| BofuT4_P018540.1 | BC1G_00879 | similar to phosphatidylglycerol specific phospholipase C | 3.77E-03 | 0.64 | -6.56 |
| BofuT4_P132890.1 | BC1G_03470 | similar to YogA (Alcohol dehydrogenase YogA) | 4.25E-02 | 0.60 | -6.56 |
| BofuT4_P069830.1 | BC1G_05213 | similar to cytochrome P450 monooxygenase | 1.64E-02 | 0.64 | -6.56 |
| BofuT4_P031460.1 | BC1G_01026 | similar to tripeptidyl-peptidase (SSH G04; Schulze Gronover et al. 2004) (secreted protein) | 6.24E-03 | 0.89 | -6.43 |
| BC1G_16166.1 | BC1G_16166 | similar to integral membrane protein | 2.33E-02 | 0.55 | -6.27 |
| PD0AEA3YB09CM1 |  | unknown | 2.24E-02 | 0.50 | -6.19 |
| BofuT4_P053020.1 | BC1G_01956 | similar to fungal specific transcription factor | 1.29E-02 | 0.57 | -6.18 |
| BofuT4_P099920.1 | BC1G_14743 | hypothetical protein | 3.18E-02 | 0.52 | -6.14 |
| BofuT4_P138240.1 | BC1G_07264 | similar to oxidoreductase molybdopterin binding domain-containing protein | 2.75E-03 | 0.91 | -6.11 |
| BofuT4_P115130.1 | BC1G_07867 | hypothetical protein | 5.38E-03 | 0.72 | -6.09 |
| BofuT4_P088170.1 | BC1G_06893 | similar to cytochrome P450 alkane hydroxylase | 3.25E-02 | 0.62 | -5.78 |
| BofuT4_P010280.1 | BC1G_08354 | similar to sedolisin | 1.69E-03 | 0.70 | -5.65 |
| BofuT4_P053040.1 | BC1G_01958 | hypothetical secreted protein | 8.09E-03 | 0.51 | -5.45 |
| BofuT4_P141530.1 | BC1G_02320 | hypothetical protein | 4.08E-02 | 0.48 | -5.42 |
| SCL_bt4ctg_1928_bt4ctg_1929.Contig1_v5 | | unknown | 3.09E-03 | 0.87 | -5.34 |
| BofuT4_P031720.1 |  | hypothetical protein | 1.20E-02 | 0.63 | -5.33 |
| BofuT4_P120540.1 | BC1G_03559 | hypothetical protein | 1.31E-02 | 0.59 | -5.32 |
| BofuT4_P135550.1 | BC1G_01794 | BcAP5. aspartic protease 1 (Ten Have et al. 2004; 2010) (secreted protein) | 2.16E-02 | 0.66 | -5.30 |
| BofuT4_P000390.1 | BC1G_08739 | hypothetical protein | 2.04E-02 | 0.63 | -5.24 |
| BofuT4_P064390.1 |  | predicted protein | 3.61E-02 | 0.62 | -5.23 |
| BofuT4_P118030.1 | BC1G_02790 | similar to aspergillopepsin-2 heavy chain (secreted protein) | 1.31E-03 | 0.54 | -5.22 |
| BC1G_15486.1 | BC1G_15486 | unknown | 3.61E-02 | 0.78 | -5.21 |
| BofuT4_P054370.1 | BC1G_07068 | BcAP1. aspartic protease 1 (Ten Have et al. 2004; 2010) | 2.16E-02 | 0.69 | -5.19 |
| BC1G_06786.1 | BC1G_06786 | unknown | 1.82E-02 | 0.65 | -5.17 |
| BofuT4_P089840.1 | BC1G_12867 | hypothetical protein | 3.29E-02 | 0.54 | -5.15 |
| BofuT4_P079890.1 | BC1G_03362 | similar to lipoamide acyltransferase component of branched-chain alpha-keto acid dehydrogenase complex | 1.78E-02 | 0.59 | -4.96 |
| BofuT4_P061030.1 | BC1G_02084 | similar to transcription factor Cys6 | 4.80E-02 | 0.52 | -4.63 |
| BofuT4_P060960.1 | BC1G_02094 | similar to glutamate decarboxylase | 1.55E-02 | 0.65 | -4.60 |
| BofuT4_P110360.1 | BC1G_02442 | similar to gi\|194709166\|pdb\|3CLJ\|A Chain A | 4.90E-02 | 0.52 | -4.55 |
| PD0ABA4YC15NM1r |  | unknown | 2.73E-02 | 0.53 | -4.54 |
| BofuT4_P090130.1 |  | similar to transcription factor Zn. C2H2 | 1.44E-02 | 0.60 | -4.49 |
| CL_bt4ctg_0232_005.Contig2_v5 |  | unknown | 9.66E-03 | 0.70 | -4.47 |
| BofuT4_uP030240.1 |  | predicted protein | 3.39E-02 | 0.77 | -4.47 |
| BofuT4_P055460.1 | BC1G_09971 | similar to amino acid transporter | 4.37E-02 | 0.55 | -4.41 |
| BofuT4_P085340.1 | BC1G_07029 | BcPKS20. polyketide synthase | 4.92E-02 | 0.48 | -4.34 |
| BofuT4_P106750.1 | BC1G_05517 | similar to FAD binding domain-containing protein | 1.06E-02 | 0.78 | -4.31 |
| BofuT4_P161080.1 | BC1G_11865 | similar to extracelular serine carboxypeptidase (secreted protein) | 1.20E-02 | 0.85 | -4.28 |
| BofuT4_P138440.1 |  | hypothetical protein | 5.15E-04 | 0.77 | -4.28 |
| BofuT4_P146250.1 | BC1G_05871 | similar to MFS multidrug transporter | 2.88E-02 | 0.65 | -4.07 |
| BofuT4_uP034390.1 | BC1G_14962 | hypothetical protein | 2.79E-02 | 0.49 | -4.07 |
| BofuT4_P001600.1 | BC1G_16119 | hypothetical protein | 4.56E-02 | 0.44 | -4.02 |
| BofuT4_P120520.1 | BC1G_03557 | similar to secreted protein | 8.98E-03 | 0.71 | -3.99 |
| BofuT4_P107660.1 | BC1G_05589 | similar to ABC transporter | 1.25E-02 | 0.47 | -3.95 |
| BofuT4_uP018360.1 | BC1G_00897 | predicted protein | 1.07E-02 | 0.74 | -3.92 |
| BofuT4_P126790.1 | BC1G_04396 | similar to mitochondrial ribosomal protein subunit L31 | 4.73E-02 | 0.50 | -3.91 |
| BofuT4_P132370.1 | BC1G_06760 | similar to arrestin (or S-antigen) | 3.37E-02 | 0.42 | -3.90 |
| BofuT4_P131730.1 | BC1G_06820 | similar to transcription factor bHLH | 3.68E-02 | 0.71 | -3.80 |
| BofuT4_P101100.1 | BC1G_09154 | similar to aflatoxin biosynthesis ketoreductase nor-1 | 1.53E-02 | 0.58 | -3.79 |
| BofuT4_P032680.1 | BC1G_12022 | similar to MFS sugar transporter | 4.26E-02 | 0.49 | -3.77 |
| BofuT4_P061280.1 | BC1G_02061 | hypothetical protein | 2.78E-02 | 0.44 | -3.74 |
| BofuT4_P016800.1 | BC1G_06169 | hypothetical protein | 1.63E-03 | 0.85 | -3.73 |
| BofuT4_P104330.1 | BC1G_12766 | hypothetical protein | 3.92E-02 | 0.41 | -3.63 |
| BofuT4_P087280.1 | BC1G_09523 | similar to glyceraldehyde-3-phosphate dehydrogenase | 5.57E-03 | 0.41 | -3.62 |
| BofuT4_P075030.1 | BC1G_08949 | hypothetical protein | 3.83E-02 | 0.40 | -3.59 |
| BC1G_16431.1 | BC1G_16431 | unknown | 3.59E-02 | 0.49 | -3.57 |
| BofuT4_P087370.1 | BC1G_09530 | similar to methyltransferase domain-containing protein | 1.90E-02 | 0.66 | -3.55 |
| BofuT4_uP043150.1 |  | hypothetical protein | 2.02E-02 | 0.50 | -3.53 |
| BofuT4_uP071550.1 |  | hypothetical protein | 9.10E-04 | 0.62 | -3.51 |
| BofuT4_P027980.1 | BC1G_06256 | hypothetical protein | 1.66E-03 | 0.78 | -3.32 |
| BofuT4_uP103230.1 |  | hypothetical protein | 4.33E-02 | 0.73 | -3.29 |
| BofuT4_P018960.1 | BC1G_00835 | similar to MFS multidrug transporter | 9.03E-04 | 0.81 | -3.28 |
| BofuT4_P105980.1 | BC1G_13233 | glycosyltransferase family 2 protein | 4.58E-02 | 0.40 | -3.27 |
| BofuT4_P134130.1 | BC1G_03079 | similar to small oligopeptide transporter | 3.77E-02 | 0.56 | -3.26 |
| BofuT4_P061060.1 | BC1G_02082 | hypothetical protein | 2.16E-03 | 0.78 | -3.24 |
| BofuT4_uP031480.1 | BC1G_01024 | hypothetical protein | 2.26E-02 | 0.74 | -3.20 |
| BofuT4_P104230.1 | BC1G_12776 | similar to tripeptidyl peptidase (secreted protein) | 1.77E-02 | 0.77 | -3.19 |
| BofuT4_P012710.1 |  | hypothetical protein | 4.26E-02 | 0.52 | -3.15 |
| AL116325 |  | unknown | 2.61E-03 | 0.83 | -3.12 |
| BofuT4_P091140.1 | BC1G_12060 | similar to MFS sugar transporter | 4.50E-03 | 0.93 | -3.11 |
| BofuT4_P029570.1 | BC1G_01208 | hypothetical protein | 4.96E-02 | 0.49 | -3.10 |
| BofuT4_P018460.1 | BC1G_00887 | hypothetical protein | 4.45E-02 | 0.61 | -3.02 |
| BofuT4_P118830.1 | BC1G_02723 | similar to NRPS-like enzyme | 3.47E-02 | 0.78 | -3.01 |
| BofuT4_P041440.1 | BC1G_01458 | similar to Scytalidoglutamic peptidase | 1.00E-03 | 0.90 | -2.99 |
| BofuT4_P082280.1 | BC1G_03179 | glycoside hydrolase family 3 protein | 3.68E-02 | 0.61 | -2.98 |
| BofuT4_P015850.1 | BC1G_11698 | similar to ribonuclease T2 | 1.34E-02 | 0.77 | -2.94 |
| BofuT4_P042730.1 | BC1G_01588 | hypothetical protein | 4.00E-02 | 0.40 | -2.90 |
| BofuT4_P135560.1 | BC1G_01795 | hypothetical protein | 3.39E-02 | 0.77 | -2.90 |
| BC1G_11711.1 | BC1G_11711 | similar to alpha beta hydrolase fold protein | 2.93E-02 | 0.56 | -2.87 |
| BofuT4_P013380.1 | BC1G_09828 | hypothetical protein | 1.18E-02 | 0.52 | -2.84 |
| BofuT4_P113320.1 | BC1G_09254 | hypothetical protein | 2.59E-02 | 0.59 | -2.82 |
| BofuT4_uP051730.1 | BC1G_06503 | hypothetical protein | 1.45E-02 | 0.78 | -2.79 |
| BC1G_11710.1 | BC1G_11710 | similar to alpha beta hydrolase fold protein | 1.88E-02 | 0.51 | -2.78 |
| BofuT4_P085870.1 | BC1G_15815 | similar to FAD linked oxidase domain protein | 4.73E-02 | 0.75 | -2.77 |
| BofuT4_uP120560.1 | BC1G_03561 | predicted protein | 1.93E-02 | 0.57 | -2.75 |
| BofuT4_P132040.1 |  | hypothetical protein | 5.00E-03 | 0.69 | -2.75 |
| BofuT4_P135500.1 | BC1G_01789 | similar to GMC oxidoreductase | 2.40E-02 | 0.45 | -2.73 |
| BofuT4_P054310.1 | BC1G_07073 | hypothetical protein | 3.59E-03 | 0.60 | -2.72 |
| BC1G_11150.1 | BC1G_11150 | unknown | 4.34E-02 | 0.42 | -2.71 |
| BofuT4_P051860.1 | BC1G_06522 | similar to alanyl-tRNA synthetase | 3.09E-02 | 0.58 | -2.71 |
| BofuT4_uP105950.1 | BC1G_12122 | hypothetical protein | 4.29E-02 | 0.65 | -2.70 |
| BofuT4_uP081460.1 | BC1G_03103 | hypothetical protein | 3.40E-02 | 0.57 | -2.70 |
| BofuT4_P103110.1 | BC1G_13122 | similar to OPT oligopeptide transporter | 4.24E-03 | 0.50 | -2.69 |
| BofuT4_P158270.1 |  | hypothetical protein | 5.56E-03 | 0.63 | -2.69 |
| BofuT4_P105910.1 | BC1G_13241 | hypothetical protein | 4.35E-02 | 0.46 | -2.68 |
| BofuT4_P053720.1 | BC1G_07128 | hypothetical protein | 1.65E-02 | 0.54 | -2.66 |
| BofuT4_uP106860.1 |  | hypothetical protein | 1.80E-02 | 0.61 | -2.66 |
| BC1G_14706.1 | BC1G_14706 | unknown | 1.64E-02 | 0.85 | -2.64 |
| BofuT4_uP047510.1 |  | predicted protein | 2.02E-02 | 0.44 | -2.63 |
| BofuT4_P031570.1 | BC1G_01014 | similar to short-chain dehydrogenase/reductase family oxidoreductase | 5.67E-03 | 0.48 | -2.62 |
| BC1G_03541.1 |  | unknown | 4.64E-02 | 0.59 | -2.62 |
| BofuT4_P001840.1 | BC1G_07475 | similar to MFS transporter | 1.21E-02 | 0.44 | -2.61 |
| BofuT4_P067220.1 |  | hypothetical protein | 1.63E-04 | 0.98 | -2.61 |
| BC1G_00259.1 | BC1G_00259 | unknown | 2.88E-02 | 0.58 | -2.60 |
| BofuT4_P053070.1 | BC1G_01961 | hypothetical protein | 2.37E-02 | 0.75 | -2.59 |
| BofuT4_uP006640.1 | BC1G_07784 | predicted protein | 4.28E-02 | 0.44 | -2.59 |
| BofuT4_P053030.1 | BC1G_01957 | similar to transcription factor Cys6 | 2.30E-02 | 0.83 | -2.56 |
| BC1G_05261.1 | BC1G_05261 | unknown | 2.01E-03 | 0.81 | -2.54 |
| BofuT4_uP038370.1 |  | predicted protein | 3.84E-02 | 0.54 | -2.48 |
| BofuT4_P074980.1 |  | hypothetical protein | 2.27E-02 | 0.54 | -2.46 |
| BC1G_10131.1 | BC1G_10131 | unknown | 3.83E-02 | 0.65 | -2.43 |
| BofuT4_P095270.1 | BC1G_04151 | glycoside hydrolase family 15 protein | 3.77E-03 | 0.57 | -2.42 |
| BofuT4_P048010.1 | BC1G_00217 | hypothetical protein | 1.42E-02 | 0.48 | -2.41 |
| BofuT4_uP075590.1 | BC1G_08898 | hypothetical protein | 2.70E-02 | 0.68 | -2.37 |
| BofuT4_P021780.1 | BC1G_00543 | similar to L-lactate 2-monooxygenase | 9.00E-03 | 0.53 | -2.37 |
| BofuT4_P120040.1 | BC1G_03512 | similar to C2HC5 finger protein | 2.26E-02 | 0.66 | -2.37 |
| BofuT4_uP005090.1 | BC1G_11988 | predicted protein | 1.92E-02 | 0.76 | -2.36 |
| BofuT4_P117240.1 | BC1G_15665 | hypothetical protein | 8.03E-03 | 0.77 | -2.35 |
| BC1G_02685.1 | BC1G_02685 | unknown | 9.63E-03 | 0.44 | -2.34 |
| BofuT4_P086010.1 | BC1G_15481 | similar to oxidoreductase | 4.80E-02 | 0.42 | -2.31 |
| BofuT4_P077820.1 | BC1G_06689 | hypothetical protein | 1.35E-02 | 0.76 | -2.31 |
| BofuT4_P034180.1 | BC1G_08227 | BcPKS14. polyketide synthase. partial sequence | 4.46E-02 | 0.41 | -2.30 |
| BofuT4_P090160.1 | BC1G_12897 | similar to purine-cytosine permease | 3.57E-02 | 0.78 | -2.26 |
| BC1G_09707.1 | BC1G_09707 | unknown | 3.41E-02 | 0.62 | -2.25 |
| BC1G_15930.1 | BC1G_15930 | similar to homeobox transcription | 4.12E-02 | 0.70 | -2.24 |
| BofuT4_uP054340.1 | BC1G_07071 | predicted protein | 4.98E-02 | 0.65 | -2.24 |
| BofuT4_P158250.1 | BC1G_09282 | hypothetical protein | 4.91E-02 | 0.53 | -2.24 |
| BC1G_06782.1 | BC1G_06782 | unknown | 3.24E-03 | 0.82 | -2.23 |
| BofuT4_uP018480.1 |  | hypothetical protein | 1.13E-02 | 0.66 | -2.23 |
| BofuT4_P086900.1 |  | predicted protein | 1.49E-02 | 0.82 | -2.22 |
| BofuT4_P088950.1 | BC1G_13362 | hypothetical protein | 2.06E-02 | 0.42 | -2.22 |
| BC1G_14369.1 | BC1G_14369 | unknown | 4.23E-03 | 0.71 | -2.21 |
| BofuT4_uP122640.1 |  | hypothetical protein | 4.40E-02 | 0.63 | -2.21 |
| PD0AGA17YH23CM1 |  | unknown | 3.29E-02 | 0.63 | -2.21 |
| BofuT4_P112580.1 | BC1G_09180 | similar to metalloproteinase (SSH8; Schulze Gronover et al. 2004) (secreted protein) | 4.22E-02 | 0.45 | -2.20 |
| BofuT4_P053510.1 | BC1G_07149 | similar to serine carboxypeptidase (CpdS) (secreted protein) | 2.43E-02 | 0.76 | -2.18 |
| BofuT4_uP146170.1 | BC1G_05865 | predicted protein | 2.99E-02 | 0.68 | -2.18 |
| BofuT4_P131880.1 |  | hypothetical protein | 4.86E-02 | 0.70 | -2.18 |
| BofuT4_uP037820.1 |  | predicted protein | 2.11E-02 | 0.62 | -2.17 |
| BofuT4_P085800.1 |  | hypothetical protein | 6.37E-04 | 0.63 | -2.13 |
| BofuT4_uP026910.1 |  | predicted protein | 4.73E-02 | 0.42 | -2.12 |
| BC1G_01894.1 | BC1G_01894 | unknown | 4.10E-02 | 0.48 | -2.11 |
| BofuT4_P156900.1 | BC1G_13762 | carbohydrate esterase family 5 protein | 3.55E-02 | 0.69 | -2.10 |
| BofuT4_P007520.1 | BC1G_03788 | hypothetical protein | 1.93E-02 | 0.76 | -2.10 |
| BC1G_06009.1 | BC1G_06009 | unknown | 3.14E-02 | 0.55 | -2.09 |
| BofuT4_uP000130.1 |  | hypothetical protein | 2.42E-02 | 0.50 | -2.09 |
| BofuT4_P003680.1 | BC1G_02956 | hypothetical protein | 1.76E-02 | 0.61 | -2.08 |
| BofuT4_uP105960.1 |  | hypothetical protein | 2.92E-02 | 0.48 | -2.07 |
| BC1G_16112.1 | BC1G_16112 | unknown | 3.06E-02 | 0.55 | -2.07 |
| BofuT4_P108510.1 | BC1G_02621 | similar to stress response RCI peptide | 4.50E-02 | 0.71 | -2.07 |
| AL111392 |  | unknown | 4.36E-02 | 0.58 | -2.04 |
| BofuT4_P032260.1 | BC1G_00951 | hypothetical protein | 4.52E-03 | 0.87 | -2.03 |
| BofuT4_P158760.1 | BC1G_09332 | hypothetical protein | 2.80E-02 | 0.64 | -2.02 |
| BC1G_08082.1 | BC1G_08082 | unknown | 3.77E-02 | 0.60 | -2.02 |
| BofuT4_P055520.1 | BC1G_09965 | hypothetical protein | 4.87E-02 | 0.42 | -2.02 |
| BofuT4_uP090330.1 |  | predicted protein | 2.02E-02 | 0.63 | -2.02 |
| BofuT4_uP048810.1 |  | hypothetical protein | 3.17E-02 | 0.44 | -2.01 |

* Genes whose expression profiles were confirmed by northern blot analyses (Fig. 9).
