# Supplementary material for: Natural Variation in the VELVET Gene bcvel1 Affects Virulence and Light-Dependent Differentiation in Botrytis cinerea
Source: PLoS One. 2012 Oct 31;7(10):e47840. doi: 10.1371/journal.pone.0047840 (PMC3485325; doi:10.1371/journal.pone.0047840)
Supplement: Table S2 — List of the 419 over-expressed genes in Δ bcvel1 mutant. (DOCX) [file pone.0047840.s013.docx]

**Table S2.** List of the 419 over-expressed genes in ∆*bcvel1* mutant.

| **SEQ_ID** | **Broad annotation** | **Function** | **ANOVA**  **p-value** | **ANOVA Normalized Variance** | **FoldChange_∆bcvel1_DIV_BY_WT** |
| --- | --- | --- | --- | --- | --- |
| BofuT4_uP018180.1 |  | hypothetical protein | 6.89E-03 | 0.93 | 78.25 |
| BofuT4_P092520.1 | BC1G_14274 | similar to amino acid transporter | 1.59E-03 | 0.92 | 67.40 |
| BofuT4_P031660.1 | BC1G_01007 | glycoside hydrolase family 61 protein | 3.54E-02 | 0.55 | 61.13 |
| BofuT4_uP003470.1 |  | predicted protein | 5.01E-04 | 0.95 | 52.63 |
| BC1G_00914.1 | BC1G_00914 | unknown | 1.03E-02 | 0.90 | 51.54 |
| **BofuT4_P130030.1*** | **BC1G_05080** | **similar to methyltransferase LaeA** | 4.17E-03 | 0.93 | 49.91 |
| BofuT4_P134540.1 | BC1G_01704 | similar to MFS sugar transporter | 1.01E-03 | 0.72 | 45.90 |
| BofuT4_P130040.1 |  | predicted protein | 2.08E-06 | 1.00 | 45.20 |
| BofuT4_P021060.1 | BC1G_00614 | hypothetical protein | 2.44E-02 | 0.76 | 44.80 |
| BofuT4_P114330.1 | BC1G_15052 | hypothetical protein | 4.06E-02 | 0.62 | 39.70 |
| BofuT4_P066310.1 | BC1G_11540 | similar to P-type ATPase. partial sequence | 2.05E-03 | 0.96 | 38.36 |
| BofuT4_P022980.1 | BC1G_03639 | similar to L-fucose permease Glucose/galactose transporter | 7.56E-03 | 0.66 | 36.87 |
| BC1G_00913.1 | BC1G_00913 | unknown | 1.52E-03 | 0.97 | 35.10 |
| BofuT4_P091380.1 | BC1G_15017 | glycoside hydrolase family 61 protein | 2.51E-02 | 0.80 | 33.61 |
| BofuT4_P008430.1 | BC1G_03881 | glycoside hydrolase family 76 protein | 3.24E-03 | 0.92 | 32.05 |
| BofuT4_P048870.1 | BC1G_00129 | similar to TPA: TPA_inf: GPR1/FUN34/YaaH-class plasma membrane protein | 2.33E-02 | 0.83 | 27.27 |
| BofuT4_P048780.1 | BC1G_00141 | hypothetical protein | 2.57E-02 | 0.70 | 27.09 |
| BofuT4_P006540.1 | BC1G_07794 | carbohydrate-binding module family 1 | 3.00E-02 | 0.61 | 25.40 |
| BofuT4_P157520.1 | BC1G_11643 | hypothetical protein | 2.53E-03 | 0.85 | 25.13 |
| BC1G_02975.1 | BC1G_02975 | unknown | 6.82E-04 | 0.90 | 24.37 |
| BofuT4_P040640.1 | BC1G_01378 | hypothetical protein | 6.14E-03 | 0.89 | 23.34 |
| BofuT4_P080920.1 | BC1G_04611 | similar to ammonium transporter | 1.47E-02 | 0.64 | 22.90 |
| BC1G_14688.1 | BC1G_14688 | unknown | 3.53E-02 | 0.60 | 22.85 |
| BofuT4_P023170.1 | BC1G_03623 | hypothetical protein | 2.53E-02 | 0.82 | 21.72 |
| BofuT4_P038520.1 | BC1G_12831 | hypothetical protein | 5.48E-03 | 0.93 | 21.30 |
| BofuT4_P012780.1 | BC1G_09891 | similar to MFS sugar transporter | 9.83E-04 | 0.96 | 19.66 |
| BofuT4_P056670.1 | BC1G_12139 | similar to carotenoid ester lipase precursor (secreted protein) | 1.51E-02 | 0.78 | 19.62 |
| BofuT4_P028710.1 | BC1G_01295 | similar to gi\|310689659\|pdb\|2XM7\|A Chain A | 1.33E-03 | 0.97 | 18.59 |
| BofuT4_P128280.1 | BC1G_04685 | hypothetical protein | 5.32E-03 | 0.86 | 18.54 |
| BofuT4_P109960.1 | BC1G_02483 | hypothetical protein | 4.43E-03 | 0.94 | 18.51 |
| BofuT4_P030350.1 | BC1G_01124 | hypothetical protein | 4.37E-03 | 0.84 | 17.86 |
| BofuT4_P114320.1 |  | hypothetical protein | 4.61E-02 | 0.53 | 17.49 |
| BofuT4_P064370.1 | BC1G_10791 | similar to major allergen Asp f 2 | 8.92E-04 | 0.91 | 17.46 |
| BofuT4_P018200.1 |  | hypothetical protein | 2.66E-02 | 0.76 | 17.34 |
| BofuT4_P096230.1 | BC1G_14308 | BcSTC4. similar to sesquiterpene cyclase | 2.50E-02 | 0.77 | 16.41 |
| BofuT4_P046350.1 | BC1G_00369 | similar to nitrate transporter | 2.08E-03 | 0.66 | 15.97 |
| BofuT4_P104690.1 | BC1G_13212 | hypothetical protein | 2.03E-02 | 0.85 | 15.59 |
| BofuT4_P139340.1 | BC1G_12563 | glycoside hydrolase family 16 protein | 2.76E-02 | 0.55 | 15.39 |
| BC1G_14679.1 | BC1G_14679 | unknown | 2.08E-02 | 0.84 | 15.34 |
| BofuT4_P105000.1 |  | hypothetical protein | 1.55E-02 | 0.74 | 15.12 |
| BofuT4_P010840.1 | BC1G_08406 | hypothetical protein | 4.44E-02 | 0.61 | 14.73 |
| BofuT4_P025780.1 | BC1G_07622 | glycoside hydrolase family 3 protein | 4.60E-02 | 0.71 | 14.25 |
| BofuT4_P081400.1 | BC1G_03097 | similar to lipase (secreted protein) | 2.70E-03 | 0.71 | 14.12 |
| BofuT4_P028100.1 | BC1G_06268 | similar to MFS sugar transporter | 4.23E-02 | 0.66 | 13.55 |
| BofuT4_P033260.1 | BC1G_10474 | hypothetical protein | 1.69E-03 | 0.82 | 13.40 |
| BofuT4_P025320.1 | BC1G_07666 | hypothetical protein | 2.50E-02 | 0.78 | 13.10 |
| BofuT4_P035890.1 | BC1G_03983 | glycoside hydrolase family 55 protein | 2.95E-02 | 0.82 | 12.73 |
| BofuT4_P116590.1 | BC1G_09772 | NiaD. nitrate reductase | 5.91E-04 | 0.48 | 12.67 |
| BofuT4_P025330.1 | BC1G_07665 | similar to cytochrome p450 | 6.04E-03 | 0.89 | 12.61 |
| BofuT4_P151840.1 | BC1G_10717 | similar to MFS sugar transporter | 3.04E-02 | 0.65 | 12.22 |
| BC1G_13690.1 | BC1G_13690 | unknown | 3.38E-02 | 0.71 | 12.15 |
| BC1G_05187.1 | BC1G_05187 | unknown | 2.80E-03 | 0.75 | 12.14 |
| PD0ABA5YK05FM1 |  | unknown | 1.23E-02 | 0.69 | 12.12 |
| BofuT4_P076100.1 | BC1G_08853 | similar to amino acid transporter | 9.61E-04 | 0.95 | 12.12 |
| BofuT4_P027680.1 |  | hypothetical protein | 2.08E-02 | 0.83 | 11.89 |
| BofuT4_P094300.1 | BC1G_04238 | similar to ferric-chelate reductase | 2.63E-02 | 0.44 | 11.79 |
| BofuT4_P077790.1 | BC1G_06692 | hypothetical protein | 3.63E-02 | 0.72 | 11.72 |
| BofuT4_P127950.1 | BC1G_04653 | hypothetical protein | 1.70E-02 | 0.83 | 11.69 |
| BofuT4_P100860.1 | BC1G_09127 | similar to ammonium transporter | 4.96E-03 | 0.70 | 11.41 |
| BofuT4_P129350.1 | BC1G_14813 | hypothetical protein | 2.36E-02 | 0.70 | 11.17 |
| BofuT4_P006320.1 | BC1G_07814 | similar to MFS transporter | 1.83E-02 | 0.62 | 11.13 |
| BofuT4_P159760.1 | BC1G_11205 | similar to MFS multidrug transporter | 1.93E-03 | 0.78 | 11.13 |
| BC1G_14710.1 | BC1G_14710 | unknown | 2.15E-02 | 0.53 | 11.01 |
| BofuT4_P064000.1 | BC1G_12455 | hypothetical protein | 5.22E-03 | 0.90 | 10.91 |
| BofuT4_P033270.1 | BC1G_10473 | hypothetical protein | 2.03E-02 | 0.78 | 10.80 |
| BofuT4_P129360.1 | BC1G_14814 | similar to flavin-binding monooxygenase | 4.14E-02 | 0.65 | 10.79 |
| BofuT4_P085130.1 | BC1G_07052 | Polysaccharide Lyase family 3 protein | 1.88E-02 | 0.78 | 10.65 |
| BofuT4_P059480.1 | BC1G_09013 | similar to lipase (secreted protein) | 4.96E-03 | 0.64 | 10.63 |
| BofuT4_P106510.1 | BC1G_05488 | similar to GPI anchored protein | 2.67E-02 | 0.56 | 10.54 |
| BofuT4_P111840.1 |  | hypothetical protein | 4.48E-02 | 0.73 | 10.47 |
| BofuT4_P157700.1 | BC1G_11627 | hypothetical protein | 1.77E-02 | 0.81 | 10.46 |
| BofuT4_P152720.1 |  | hypothetical protein | 4.50E-04 | 0.98 | 10.33 |
| BofuT4_P117020.1 | BC1G_09728 | hypothetical protein | 9.77E-03 | 0.88 | 10.10 |
| BofuT4_P035490.1 | BC1G_03944 | hypothetical protein | 9.98E-03 | 0.77 | 10.06 |
| BofuT4_P154870.1 |  | similar to MFS multidrug transporter | 4.20E-02 | 0.74 | 9.75 |
| BofuT4_P144220.1 | BC1G_04494 | similar to MFS multidrug transporter | 4.77E-02 | 0.62 | 9.73 |
| BofuT4_P035820.1 | BC1G_03977 | hypothetical protein | 3.28E-02 | 0.52 | 9.69 |
| PD0AEA12YD11CM1 |  | unknown | 2.64E-02 | 0.83 | 9.69 |
| AL116289 |  | unknown | 2.31E-02 | 0.65 | 9.60 |
| BC1G_14540.1 | BC1G_14540 | unknown | 5.20E-03 | 0.93 | 9.36 |
| BofuT4_P112360.1 | BC1G_13111 | hypothetical protein | 3.13E-03 | 0.81 | 9.20 |
| BofuT4_P149250.1 | BC1G_04830 | similar to P-type ATPase | 1.67E-03 | 0.87 | 9.03 |
| BofuT4_P108910.1 | BC1G_02580 | hypothetical protein | 4.55E-02 | 0.58 | 9.01 |
| BofuT4_P119890.1 | BC1G_03500 | similar to xanthine/uracil permease family protein | 3.08E-02 | 0.58 | 8.88 |
| BofuT4_P161780.1 | BC1G_10506 | hypothetical protein | 2.51E-02 | 0.75 | 8.72 |
| BofuT4_P119740.1 | BC1G_14568 | similar to salicylate hydroxylase | 1.57E-02 | 0.74 | 8.66 |
| BofuT4_P095740.1 | BC1G_04111 | hypothetical protein | 4.71E-02 | 0.68 | 8.19 |
| BofuT4_P050540.1 | BC1G_14986 | similar to amid-like NADH oxidoreductase | 1.75E-02 | 0.72 | 8.04 |
| BofuT4_P111520.1 | BC1G_05673 | similar to MFS sugar transporter | 1.48E-02 | 0.78 | 7.94 |
| BofuT4_P064350.1 | BC1G_02167 | hypothetical protein | 2.74E-03 | 0.55 | 7.79 |
| BofuT4_P098800.1 | BC1G_15342 | similar to P-type ATPase | 1.66E-03 | 0.93 | 7.66 |
| BofuT4_P031190.1 | BC1G_01048 | similar to QUTD_EMENI Quinate permease (Quinate transporter) | 7.23E-03 | 0.93 | 7.57 |
| BofuT4_P119800.1 | BC1G_14562 | hypothetical protein | 2.91E-02 | 0.77 | 7.42 |
| BC1G_05337.1 | BC1G_05337 | unknown | 3.74E-02 | 0.66 | 7.39 |
| BC1G_16396.1 | BC1G_16396 | unknown | 4.93E-03 | 0.79 | 7.37 |
| BofuT4_P091970.1 | BC1G_13497 | similar to bacterial integral membrane protein-like protein | 1.93E-02 | 0.69 | 7.35 |
| BofuT4_P047310.1 | BC1G_00282 | hypothetical protein | 2.69E-02 | 0.78 | 7.31 |
| BofuT4_P099600.1 | BC1G_15584 | hypothetical protein | 2.55E-02 | 0.73 | 7.31 |
| BofuT4_P019630.1 | BC1G_00764 | hypothetical protein | 6.42E-04 | 0.68 | 7.26 |
| BofuT4_P148550.1 | BC1G_09413 | hypothetical protein | 2.90E-02 | 0.56 | 7.19 |
| B5BC_116_286_D01 |  | unknown | 2.41E-02 | 0.78 | 7.03 |
| BofuT4_P138800.1 | BC1G_14487 | hypothetical protein | 3.60E-02 | 0.63 | 6.96 |
| **BofuT4_P064600.1*** | **BC1G_10771** | **similar to MFS sugar transporter** | 2.14E-03 | 0.71 | 6.95 |
| BofuT4_P155180.1 | BC1G_11019 | similar to FAD dependent oxidoreductase domain containing protein | 3.28E-02 | 0.67 | 6.93 |
| BofuT4_P157540.1 | BC1G_11641 | similar to acyl-CoA dehydrogenase domain protein | 1.16E-02 | 0.85 | 6.93 |
| BofuT4_P030940.1 |  | similar to spherulin 4-like cell surface protein | 4.10E-02 | 0.75 | 6.90 |
| BC1G_13699.1 | BC1G_13699 | unknown | 9.03E-03 | 0.82 | 6.87 |
| BofuT4_P093510.1 | BC1G_10437 | hypothetical protein | 1.39E-02 | 0.60 | 6.74 |
| BofuT4_P063040.1 | BC1G_06419 | hypothetical protein | 1.92E-03 | 0.80 | 6.60 |
| BofuT4_P062430.1 | BC1G_06364 | similar to histidine acid phosphatase | 1.92E-02 | 0.63 | 6.60 |
| BofuT4_P025340.1 | BC1G_07663 | similar to MFS multidrug transporter | 2.13E-03 | 0.90 | 6.59 |
| BofuT4_P089110.1 | BC1G_11120 | similar to short chain dehydrogenase/reductase family oxidoreductase | 1.10E-02 | 0.85 | 6.58 |
| BofuT4_P155710.1 | BC1G_10974 | similar to transcription factor Cys6 | 1.13E-02 | 0.79 | 6.54 |
| BofuT4_P103370.1 | BC1G_13148 | hypothetical protein | 2.46E-03 | 0.67 | 6.54 |
| BofuT4_P004370.1 | BC1G_02893 | hypothetical protein | 4.54E-02 | 0.54 | 6.53 |
| BofuT4_P104670.1 | BC1G_13214 | similar to cytochrome P450 monooxygenase | 2.63E-02 | 0.75 | 6.35 |
| BofuT4_uP083260.1 | BC1G_10392 | hypothetical protein | 2.87E-02 | 0.61 | 6.29 |
| BofuT4_P096200.1 | BC1G_14311 | hypothetical protein | 1.35E-02 | 0.63 | 6.26 |
| BofuT4_P023790.1 | BC1G_10796 | glycosyltransferase family 1 protein | 9.57E-03 | 0.83 | 6.26 |
| CL_bt4ctg_2232_003.Contig1_v5 | | unknown | 7.11E-03 | 0.88 | 6.24 |
| BofuT4_P032200.1 | BC1G_00955 | similar to 4-coumarate-CoA ligase | 3.32E-02 | 0.56 | 6.21 |
| BofuT4_P092570.1 | BC1G_14268 | hypothetical protein | 4.59E-02 | 0.55 | 6.21 |
| BofuT4_P159470.1 | BC1G_13586 | similar to transcription factor Cys6 | 2.83E-02 | 0.69 | 5.99 |
| BofuT4_P056660.1 | BC1G_12140 | similar to copper radical oxidase | 1.15E-03 | 0.94 | 5.97 |
| BofuT4_P085780.1 |  | hypothetical protein | 1.18E-02 | 0.67 | 5.96 |
| CL_bt4exctg_0590_001.Contig1_v5 | | unknown | 2.03E-02 | 0.62 | 5.88 |
| BofuT4_P152950.1 | BC1G_08037 | similar to gamma-tocopherol methyltransferase | 1.54E-02 | 0.62 | 5.80 |
| BofuT4_P142530.1 | BC1G_02215 | hypothetical protein | 4.91E-02 | 0.54 | 5.68 |
| BofuT4_P091990.1 |  | hypothetical protein | 3.28E-02 | 0.74 | 5.55 |
| BofuT4_P056520.1 | BC1G_12157 | similar to agmatinase | 2.88E-02 | 0.64 | 5.53 |
| BofuT4_P036010.1 | BC1G_03993 | hypothetical protein | 4.14E-03 | 0.74 | 5.49 |
| BofuT4_P008690.1 | BC1G_03906 | hypothetical protein | 3.77E-02 | 0.67 | 5.47 |
| BofuT4_P012180.1 | BC1G_12533 | hypothetical protein | 1.33E-02 | 0.85 | 5.45 |
| BofuT4_P076170.1 | BC1G_08846 | similar to glucose-methanol-choline (gmc) oxidoreductase | 4.55E-02 | 0.65 | 5.42 |
| BofuT4_P111160.1 | BC1G_05709 | hypothetical protein | 3.52E-05 | 0.72 | 5.42 |
| BofuT4_P037200.1 | BC1G_11181 | hypothetical protein | 4.59E-02 | 0.73 | 5.38 |
| BofuT4_P095080.1 | BC1G_04168 | similar to amino acid transporter | 2.75E-03 | 0.94 | 5.38 |
| BofuT4_P061260.1 | BC1G_02064 | hypothetical protein | 1.66E-02 | 0.60 | 5.34 |
| BofuT4_P092040.1 | BC1G_10342 | hypothetical protein | 2.47E-02 | 0.63 | 5.32 |
| BofuT4_P078630.1 | BC1G_14370 | similar to transcription factor LPS-induced tumor necrosis factor alpha | 9.09E-03 | 0.79 | 5.25 |
| BofuT4_P084070.1 | BC1G_07530 | BcABA2. cytochrome P450 monooxygenase | 4.08E-02 | 0.73 | 5.22 |
| BofuT4_P156540.1 | BC1G_11255 | glycoside hydrolase family 1 protein | 3.76E-02 | 0.48 | 5.21 |
| BofuT4_P103360.1 |  | hypothetical protein | 2.59E-02 | 0.72 | 5.14 |
| BofuT4_P119190.1 | BC1G_02687 | similar to alcohol oxidase | 1.94E-02 | 0.81 | 5.09 |
| BofuT4_P155720.1 | BC1G_10973 | hypothetical protein | 4.12E-02 | 0.71 | 5.08 |
| CL_bt4ctg_0708_009.Contig1_v5 |  | unknown | 8.00E-03 | 0.70 | 5.08 |
| BofuT4_P038510.1 | BC1G_12830 | similar to 2-hydroxychromene-2-carboxylate isomerase | 3.90E-02 | 0.75 | 5.08 |
| PD0ABA13YJ17NM1r |  | unknown | 2.04E-02 | 0.76 | 5.03 |
| BofuT4_P100850.1 | BC1G_09125 | similar to flavin-containing superfamily Amine oxidase | 1.41E-02 | 0.59 | 5.02 |
| BofuT4_P156110.1 | BC1G_11294 | similar to amino acid transporter | 6.60E-03 | 0.90 | 4.99 |
| BofuT4_P092030.1 | BC1G_10343 | hypothetical protein | 2.01E-02 | 0.68 | 4.97 |
| BC1G_07667.1 | BC1G_07667 | unknown | 1.54E-02 | 0.82 | 4.96 |
| BofuT4_uP162510.1 |  | hypothetical protein | 2.60E-02 | 0.45 | 4.95 |
| BC1G_08092.1 | BC1G_08092 | unknown | 2.05E-02 | 0.76 | 4.88 |
| BofuT4_P000830.1 | BC1G_08700 | hypothetical protein | 1.83E-02 | 0.83 | 4.87 |
| BofuT4_P073830.1 | BC1G_05363 | hypothetical protein | 1.19E-03 | 0.68 | 4.81 |
| CL_bt4ctg_2078_001.Contig1_v5 | | unknown | 2.21E-03 | 0.84 | 4.80 |
| BofuT4_P114780.1 | BC1G_07840 | similar to 2-haloalkanoic acid dehalogenase | 2.96E-02 | 0.76 | 4.78 |
| BofuT4_P042570.1 | BC1G_01571 | hypothetical protein | 1.92E-02 | 0.75 | 4.76 |
| BofuT4_P053090.1 | BC1G_07186 | glycoside hydrolase family 76 protein | 6.10E-03 | 0.94 | 4.76 |
| BofuT4_P081610.1 | BC1G_03118 | similar to xanthine dehydrogenase | 2.65E-02 | 0.52 | 4.73 |
| BofuT4_P150530.1 | BC1G_10883 | hypothetical protein | 5.11E-03 | 0.74 | 4.73 |
| BofuT4_P086630.1 | BC1G_10229 | Polysaccharide Lyase family 3 protein | 5.42E-03 | 0.76 | 4.71 |
| BofuT4_uP113970.1 |  | predicted protein | 9.67E-03 | 0.73 | 4.70 |
| BofuT4_P107080.1 | BC1G_05542 | hypothetical protein | 1.59E-02 | 0.51 | 4.67 |
| BofuT4_P076150.1 | BC1G_08848 | similar to transcription factor Zn. C2H2 / Cys6 | 3.39E-02 | 0.77 | 4.67 |
| BofuT4_P061240.1 | BC1G_02065 | similar to AAA family ATPase | 4.95E-02 | 0.63 | 4.66 |
| BofuT4_P092010.1 | BC1G_10345 | similar to oxidoreductase | 1.45E-02 | 0.63 | 4.66 |
| BofuT4_P161410.1 | BC1G_11834 | similar to dienelactone hydrolase family protein | 4.44E-02 | 0.74 | 4.66 |
| BofuT4_P150680.1 | BC1G_13490 | similar to NADP-specific glutamate dehydrogenase | 1.46E-02 | 0.46 | 4.65 |
| BofuT4_P044540.1 | BC1G_13282 | hypothetical protein | 3.64E-02 | 0.74 | 4.61 |
| BofuT4_P025640.1 | BC1G_07637 | similar to lipase | 3.62E-02 | 0.72 | 4.60 |
| BofuT4_P068710.1 |  | hypothetical protein | 2.65E-02 | 0.66 | 4.59 |
| BofuT4_P061000.1 | BC1G_02088 | similar to MFS sugar transporter | 2.26E-02 | 0.56 | 4.58 |
| BC1G_08133.1 | BC1G_08133 | unknown | 3.35E-02 | 0.57 | 4.56 |
| BofuT4_P038620.1 | BC1G_12839 | hypothetical protein | 1.98E-02 | 0.74 | 4.55 |
| BC1G_14793.1 | BC1G_14793 | unknown | 4.56E-02 | 0.52 | 4.52 |
| BofuT4_P015210.1 | BC1G_16098 | hypothetical protein | 3.44E-02 | 0.72 | 4.51 |
| BofuT4_P035320.1 | BC1G_06441 | hypothetical protein | 1.44E-03 | 0.78 | 4.49 |
| BofuT4_P053820.1 |  | hypothetical protein | 1.82E-02 | 0.55 | 4.46 |
| BofuT4_P156520.1 | BC1G_11257 | similar to MFS sugar transporter | 1.11E-03 | 0.45 | 4.42 |
| BC1G_15988.1 | BC1G_15988 | unknown | 4.70E-02 | 0.61 | 4.41 |
| BofuT4_uP127940.1 |  | hypothetical protein | 2.00E-02 | 0.51 | 4.40 |
| B3BC_116_232_G12 |  | unknown | 9.32E-03 | 0.59 | 4.40 |
| BofuT4_P059020.1 | BC1G_08460 | hypothetical protein | 1.91E-03 | 0.72 | 4.39 |
| BofuT4_P120670.1 | BC1G_03571 | similar to flavin-containing monooxygenase | 2.13E-02 | 0.49 | 4.39 |
| BofuT4_P129320.1 | BC1G_14810 | similar to O-methyltransferase | 3.27E-02 | 0.81 | 4.38 |
| BofuT4_uP040650.1 |  | hypothetical protein | 2.88E-02 | 0.52 | 4.36 |
| BofuT4_P033220.1 | BC1G_10477 | hypothetical protein | 3.75E-02 | 0.76 | 4.34 |
| BofuT4_P026360.1 | BC1G_09670 | similar to glucose-methanol-choline (gmc) oxidoreductase | 4.22E-02 | 0.52 | 4.34 |
| BofuT4_P011720.1 | BC1G_02144 | similar to alcohol oxidase | 4.57E-03 | 0.71 | 4.33 |
| BofuT4_P022300.1 | BC1G_03707 | similar to MFS sugar transporter | 5.17E-03 | 0.46 | 4.28 |
| BC1G_08667.1 | BC1G_08667 | unknown | 1.31E-02 | 0.67 | 4.23 |
| BofuT4_P086510.1 |  | hypothetical protein | 1.57E-03 | 0.73 | 4.21 |
| BofuT4_P106600.1 | BC1G_05499 | hypothetical protein | 1.30E-02 | 0.63 | 4.20 |
| BofuT4_P090400.1 | BC1G_14866 | similar to NAD binding Rossmann fold oxidoreductase | 3.57E-02 | 0.69 | 4.19 |
| BC1G_11262.1 | BC1G_11262 | unknown | 7.03E-03 | 0.46 | 4.19 |
| BofuT4_P032210.1 | BC1G_00954 | similar to oligopeptide transporter | 1.26E-02 | 0.42 | 4.18 |
| BofuT4_P148930.1 | BC1G_15169 | hypothetical protein | 1.71E-02 | 0.47 | 4.17 |
| BofuT4_P060120.1 | BC1G_11059 | similar to type 11 methyltransferase | 2.53E-02 | 0.68 | 4.16 |
| BofuT4_P090430.1 | BC1G_14863 | hypothetical protein | 2.42E-02 | 0.67 | 4.15 |
| BofuT4_P002650.1 | BC1G_07402 | similar to MFS multidrug transporter | 3.39E-02 | 0.62 | 4.14 |
| BofuT4_P134870.1 | BC1G_01735 | hypothetical protein | 4.85E-03 | 0.73 | 4.14 |
| BofuT4_P030260.1 | BC1G_01143 | hypothetical protein | 8.29E-03 | 0.84 | 4.12 |
| BofuT4_P046040.1 | BC1G_00404 | similar to ammonium transporter | 3.43E-02 | 0.43 | 4.12 |
| BofuT4_P155530.1 |  | hypothetical protein | 3.69E-02 | 0.46 | 4.10 |
| BC1G_14229.1 | BC1G_14229 | unknown | 1.42E-02 | 0.58 | 4.08 |
| BofuT4_P045670.1 | BC1G_00433 | hypothetical protein | 2.34E-02 | 0.72 | 4.06 |
| BofuT4_P076160.1 | BC1G_08847 | similar to isoflavone reductase family protein CipA | 6.41E-03 | 0.82 | 4.05 |
| BofuT4_P042560.1 | BC1G_01570 | similar to amino acid transporter | 3.84E-02 | 0.59 | 4.03 |
| BofuT4_P016180.1 | BC1G_11727 | hypothetical protein | 3.54E-03 | 0.53 | 4.02 |
| BofuT4_P043530.1 |  | hypothetical protein | 1.51E-02 | 0.61 | 4.02 |
| BofuT4_P066090.1 | BC1G_11559 | hypothetical protein | 3.16E-02 | 0.65 | 4.02 |
| BofuT4_P048970.1 |  | predicted protein | 4.76E-02 | 0.58 | 3.99 |
| BofuT4_P115070.1 | BC1G_07863 | similar to high-affinity nicotinic acid transporter | 4.81E-03 | 0.73 | 3.98 |
| BofuT4_P161310.1 | BC1G_11847 | hypothetical protein | 2.57E-02 | 0.78 | 3.98 |
| BofuT4_P014290.1 | BC1G_08308 | hypothetical protein | 3.59E-02 | 0.77 | 3.96 |
| BofuT4_P157560.1 | BC1G_11639 | similar to short-chain dehydrogenase/reductase SDR | 1.08E-02 | 0.79 | 3.94 |
| BofuT4_uP007260.1 |  | hypothetical protein | 5.47E-03 | 0.72 | 3.92 |
| BofuT4_uP099220.1 |  | hypothetical protein | 3.41E-02 | 0.61 | 3.91 |
| PD0ABA5YN12NM1r |  | unknown | 4.62E-02 | 0.48 | 3.91 |
| BofuT4_P146350.1 | BC1G_05881 | BcATRO. ABC transporter | 1.55E-02 | 0.56 | 3.90 |
| BofuT4_P055930.1 | BC1G_09930 | similar to regulator of G protein | 3.07E-02 | 0.45 | 3.90 |
| BofuT4_P079160.1 | BC1G_13407 | glycoside hydrolase family 78 protein | 3.23E-02 | 0.70 | 3.90 |
| BofuT4_P078070.1 | BC1G_06666 | glycoside hydrolase family 3 protein | 8.22E-03 | 0.65 | 3.87 |
| BofuT4_P020310.1 | BC1G_00693 | similar to serum paraoxonase/arylesterase | 3.61E-02 | 0.80 | 3.84 |
| BofuT4_P161880.1 | BC1G_10515 | hypothetical protein | 4.02E-02 | 0.60 | 3.81 |
| BofuT4_P112450.1 | BC1G_09171 | hypothetical protein | 4.44E-02 | 0.52 | 3.80 |
| BofuT4_P033390.1 | BC1G_08148 | hypothetical protein | 1.68E-02 | 0.56 | 3.80 |
| BofuT4_P019170.1 | BC1G_00813 | similar to isoaspartyl dipeptidase | 1.96E-02 | 0.44 | 3.80 |
| BofuT4_P092540.1 | BC1G_14273 | similar to YD repeat-containing protein | 2.05E-02 | 0.77 | 3.79 |
| BofuT4_P118170.1 | BC1G_02778 | similar to ThiJ/PfpI family protein | 7.38E-03 | 0.47 | 3.79 |
| BofuT4_P105810.1 | BC1G_13249 | similar to copper amine oxidase | 4.45E-02 | 0.68 | 3.74 |
| BofuT4_P019180.1 | BC1G_00812 | similar to pyridine nucleotide-disulphide oxidoreductase AMID-like | 3.94E-02 | 0.61 | 3.73 |
| BofuT4_P033280.1 | BC1G_10472 | hypothetical protein | 2.73E-02 | 0.47 | 3.71 |
| BofuT4_P053210.1 | BC1G_07175 | hypothetical protein | 6.73E-03 | 0.75 | 3.68 |
| BofuT4_P088750.1 | BC1G_13335 | hypothetical protein | 1.86E-02 | 0.55 | 3.67 |
| BofuT4_P092060.1 | BC1G_10341 | glycoside hydrolase family 26 protein | 2.79E-02 | 0.51 | 3.66 |
| BofuT4_P091390.1 | BC1G_15016 | similar to MFS monocarboxylate transporter | 2.17E-02 | 0.68 | 3.64 |
| BofuT4_P083250.1 |  | hypothetical protein | 1.24E-02 | 0.72 | 3.61 |
| BofuT4_P121700.1 | BC1G_07996 | similar to acetoin dehydrogenase (diacetyl reductase) | 3.22E-05 | 0.40 | 3.60 |
| PD0ACA10YL04FM1 |  | unknown | 3.73E-02 | 0.60 | 3.60 |
| BofuT4_P152960.1 | BC1G_08036 | hypothetical protein | 2.34E-02 | 0.78 | 3.59 |
| BofuT4_P073780.1 | BC1G_05370 | similar to alpha/beta hydrolase fold protein | 4.50E-02 | 0.59 | 3.59 |
| BofuT4_P068590.1 | BC1G_10091 | similar to carboxylesterase (secreted protein) | 3.33E-02 | 0.80 | 3.56 |
| BofuT4_P081600.1 | BC1G_03117 | hypothetical protein | 1.48E-02 | 0.75 | 3.53 |
| BC1G_08054.1 | BC1G_08054 | unknown | 1.39E-02 | 0.70 | 3.53 |
| BofuT4_P021270.1 | BC1G_00594 | glycoside hydrolase family 12 protein | 4.82E-02 | 0.68 | 3.52 |
| BofuT4_P005550.1 | BC1G_11946 | hypothetical protein | 4.61E-02 | 0.48 | 3.52 |
| BofuT4_P084520.1 | BC1G_07571 | hypothetical protein | 2.62E-02 | 0.50 | 3.49 |
| BofuT4_P108800.1 | BC1G_02591 | glycoside hydrolase family 27 protein | 1.44E-02 | 0.44 | 3.48 |
| BofuT4_P035310.1 | BC1G_06442 | hypothetical protein | 2.50E-03 | 0.64 | 3.48 |
| BofuT4_P000050.1 | BC1G_08769 | hypothetical protein | 1.73E-02 | 0.68 | 3.47 |
| BofuT4_P018220.1 | BC1G_00912 | Polysaccharide lyase family 1 protein | 4.80E-02 | 0.67 | 3.47 |
| BofuT4_P010070.1 | BC1G_12727 | similar to aminopeptidase | 2.16E-03 | 0.80 | 3.47 |
| BofuT4_P161630.1 | BC1G_11810 | similar to MFS allantoate transporter | 2.30E-02 | 0.46 | 3.44 |
| PD0AEA12YG21CM1 |  | unknown | 3.45E-02 | 0.50 | 3.43 |
| BofuT4_P045590.1 | BC1G_00437 | similar to nitrite reductase | 8.31E-03 | 0.43 | 3.40 |
| BofuT4_P034360.1 | BC1G_14966 | similar to alpha/beta hydrolase fold-3 domain-containing protein | 3.51E-02 | 0.76 | 3.40 |
| BofuT4_P009660.1 | BC1G_12684 | hypothetical protein | 2.30E-02 | 0.78 | 3.39 |
| BofuT4_P096950.1 | BC1G_11737 | hypothetical protein | 1.14E-02 | 0.55 | 3.39 |
| BofuT4_P114270.1 | BC1G_15058 | similar to Rad21/Rec8 N terminal domain-containing protein | 3.16E-02 | 0.58 | 3.32 |
| BofuT4_P089260.1 | BC1G_11133 | hypothetical protein | 8.13E-03 | 0.73 | 3.32 |
| BofuT4_P102450.1 | BC1G_14032 | predicted protein | 2.90E-02 | 0.59 | 3.30 |
| BofuT4_P035870.1 | BC1G_03981 | similar to sulfate permease | 1.32E-02 | 0.85 | 3.30 |
| BofuT4_P062780.1 | BC1G_06394 | hypothetical protein | 1.21E-02 | 0.76 | 3.28 |
| BofuT4_P121470.1 | BC1G_08018 | hypothetical protein | 2.69E-03 | 0.87 | 3.28 |
| BofuT4_P097160.1 | BC1G_11761 | hypothetical protein | 4.91E-02 | 0.69 | 3.28 |
| BofuT4_P136670.1 | BC1G_01917 | hypothetical protein | 4.35E-02 | 0.68 | 3.27 |
| BofuT4_P143310.1 | BC1G_12658 | hypothetical protein | 1.57E-02 | 0.70 | 3.26 |
| BofuT4_P019140.1 | BC1G_00815 | hypothetical protein | 2.77E-02 | 0.73 | 3.25 |
| BofuT4_P118400.1 | BC1G_02756 | similar to short chain dehydrogenase/reductase | 9.32E-03 | 0.63 | 3.23 |
| BofuT4_P095750.1 |  | hypothetical protein | 4.14E-02 | 0.52 | 3.22 |
| BofuT4_P116350.1 |  | hypothetical protein | 2.12E-02 | 0.84 | 3.21 |
| BofuT4_uP043510.1 |  | hypothetical protein | 9.41E-03 | 0.72 | 3.20 |
| BofuT4_P156440.1 |  | hypothetical protein | 2.38E-02 | 0.42 | 3.20 |
| BC1G_05836.1 | BC1G_05836 | unknown | 1.66E-02 | 0.40 | 3.17 |
| BC1G_05182.1 | BC1G_05182 | unknown | 4.95E-03 | 0.83 | 3.17 |
| BofuT4_P134860.1 | BC1G_01734 | hypothetical protein | 2.25E-02 | 0.66 | 3.11 |
| BofuT4_P053830.1 | BC1G_07120 | hypothetical protein | 1.34E-03 | 0.56 | 3.11 |
| BofuT4_uP045380.1 |  | predicted protein | 3.66E-02 | 0.50 | 3.09 |
| BofuT4_P141600.1 | BC1G_02312 | similar to alcohol dehydrogenase | 3.59E-02 | 0.68 | 3.08 |
| BofuT4_P019660.1 |  | hypothetical protein | 5.76E-03 | 0.81 | 3.08 |
| BofuT4_P073630.1 | BC1G_05383 | hypothetical protein | 3.50E-02 | 0.64 | 3.08 |
| BofuT4_P046420.1 |  | similar to MFS sugar transporter | 6.48E-03 | 0.59 | 3.08 |
| BofuT4_P147630.1 | BC1G_10827 | similar to glutaminase | 4.78E-02 | 0.72 | 3.08 |
| BofuT4_P106720.1 | BC1G_05512 | hypothetical protein | 3.30E-02 | 0.76 | 3.06 |
| BofuT4_P009670.1 | BC1G_12687 | hypothetical protein | 1.28E-02 | 0.43 | 3.05 |
| BofuT4_P049000.1 | BC1G_00118 | similar to transcription factor bZIP | 7.63E-03 | 0.56 | 3.05 |
| CL_bt4ctg_1304_011.Contig1_v5 | | unknown | 1.67E-02 | 0.67 | 3.05 |
| BC1G_00238.1 | BC1G_00238 | similar to sugar transporter | 2.43E-03 | 0.54 | 3.02 |
| BofuT4_P012200.1 | BC1G_12530 | hypothetical protein | 3.52E-02 | 0.74 | 3.02 |
| BofuT4_P158540.1 | BC1G_09312 | hypothetical protein | 1.90E-02 | 0.72 | 3.01 |
| BofuT4_P059690.1 | BC1G_09028 | similar to calpain | 9.33E-04 | 0.54 | 3.00 |
| BofuT4_P059700.1 | BC1G_09029 | hypothetical protein | 5.62E-03 | 0.84 | 3.00 |
| CL_bt4ctg_1151_008.Contig6_v5 | | unknown | 1.30E-02 | 0.49 | 3.00 |
| BC1G_13482.1 | BC1G_13482 | unknown | 1.31E-02 | 0.60 | 3.00 |
| BC1G_15164.1 | BC1G_15164 | unknown | 3.32E-02 | 0.75 | 2.99 |
| BofuT4_P117260.1 | BC1G_15663 | similar to dipeptidyl-peptidase V (secreted protein) | 4.17E-02 | 0.64 | 2.96 |
| BofuT4_uP114060.1 | BC1G_09437 | hypothetical protein | 1.21E-03 | 0.73 | 2.93 |
| CL_bt4exctg_0558_001.Contig1_v5 | | unknown | 2.15E-02 | 0.85 | 2.93 |
| BofuT4_P136590.1 | BC1G_01909 | hypothetical protein | 2.23E-02 | 0.65 | 2.92 |
| BofuT4_P131330.1 |  | hypothetical protein | 1.69E-02 | 0.72 | 2.91 |
| BofuT4_P046960.1 | BC1G_00314 | similar to MFS multidrug transporter | 4.37E-03 | 0.71 | 2.90 |
| BofuT4_P068250.1 | BC1G_10120 | hypothetical protein | 2.60E-02 | 0.78 | 2.89 |
| BofuT4_P087090.1 | BC1G_09504 | hypothetical protein | 2.44E-02 | 0.41 | 2.88 |
| BofuT4_P064720.1 | BC1G_10759 | hypothetical protein | 2.12E-02 | 0.44 | 2.87 |
| BofuT4_P015820.1 | BC1G_11693 | hypothetical protein | 8.68E-03 | 0.62 | 2.86 |
| BofuT4_P114470.1 | BC1G_15830 | similar to tyrosinase central domain protein | 4.78E-02 | 0.56 | 2.85 |
| BofuT4_uP159200.1 |  | hypothetical protein | 4.05E-02 | 0.55 | 2.84 |
| BofuT4_P068840.1 | BC1G_05306 | hypothetical protein | 3.92E-02 | 0.64 | 2.83 |
| BofuT4_P086730.1 | BC1G_10217 | hypothetical protein | 1.21E-02 | 0.54 | 2.82 |
| BofuT4_P147900.1 | BC1G_10802 | similar to beta-lactamase | 4.25E-02 | 0.44 | 2.80 |
| BofuT4_P125850.1 | BC1G_04309 | hypothetical protein | 2.93E-02 | 0.59 | 2.80 |
| BofuT4_P016920.1 | BC1G_06159 | hypothetical protein | 1.98E-03 | 0.45 | 2.79 |
| BofuT4_P115650.1 | BC1G_10620 | hypothetical protein | 5.07E-03 | 0.43 | 2.79 |
| BC1G_06412.1 | BC1G_06412 | unknown | 2.38E-02 | 0.43 | 2.78 |
| BofuT4_P002970.1 | BC1G_03020 | hypothetical protein | 4.72E-03 | 0.83 | 2.78 |
| BofuT4_P106730.1 | BC1G_05515 | similar to extracellular lipase (secreted protein) | 6.65E-04 | 0.43 | 2.77 |
| BofuT4_P018410.1 | BC1G_00892 | hypothetical protein | 5.67E-03 | 0.89 | 2.77 |
| CL_bt4ctg_0595_006.Contig2_v5 | | unknown | 1.20E-02 | 0.40 | 2.76 |
| BofuT4_P124460.1 | BC1G_05975 | similar to acetamidase | 1.89E-02 | 0.65 | 2.75 |
| BofuT4_P009610.1 | BC1G_12251 | hypothetical protein | 7.86E-03 | 0.44 | 2.74 |
| **BofuT4_P078590.1*** | **BC1G_14375** | **similar to MFS sugar transporter** | 9.61E-04 | 0.60 | 2.73 |
| BC1G_00324.1 | BC1G_00324 |  | 4.88E-02 | 0.51 | 2.71 |
| BC1G_13698.1 | BC1G_13698 | unknown | 1.80E-02 | 0.47 | 2.70 |
| BofuT4_P099050.1 | BC1G_05158 | similar to D-lactate dehydrogenase (cytochrome) | 7.44E-03 | 0.58 | 2.69 |
| BofuT4_P159540.1 | BC1G_08139 | hypothetical protein | 1.76E-02 | 0.85 | 2.68 |
| BofuT4_P081410.1 | BC1G_03099 | similar to cyclin B1 interacting protein 1 | 2.91E-02 | 0.65 | 2.67 |
| BofuT4_P053230.1 | BC1G_07173 | hypothetical protein | 1.93E-02 | 0.57 | 2.67 |
| BofuT4_P151890.1 |  | predicted protein | 3.07E-02 | 0.54 | 2.64 |
| BofuT4_P109230.1 | BC1G_02551 | glycoside hydrolase family 45 protein | 6.43E-03 | 0.58 | 2.63 |
| BofuT4_P011600.1 | BC1G_02130 | hypothetical protein | 1.98E-02 | 0.41 | 2.62 |
| BofuT4_P001710.1 | BC1G_07485 | similar to cytochrome P450 monooxygenase | 2.44E-02 | 0.58 | 2.61 |
| BofuT4_P032170.1 | BC1G_00957 | similar to methyltransferase domain-containing protein | 4.49E-04 | 0.83 | 2.60 |
| BofuT4_P031400.1 | BC1G_01030 | similar to cytochrome P450 monooxygenase | 9.95E-03 | 0.50 | 2.60 |
| BofuT4_P130020.1 | BC1G_05079 | similar to MFS transporter | 2.76E-02 | 0.50 | 2.59 |
| BC1G_10710.1 | BC1G_10710 | unknown | 2.81E-02 | 0.44 | 2.58 |
| BofuT4_P120800.1 | BC1G_03584 | hypothetical protein | 3.73E-02 | 0.67 | 2.57 |
| BofuT4_P008930.1 | BC1G_03932 | hypothetical protein | 4.95E-02 | 0.70 | 2.57 |
| BofuT4_P163430.1 | BC1G_13902 | similar to MFS multidrug transporter | 4.67E-02 | 0.61 | 2.54 |
| BofuT4_P002470.1 | BC1G_07416 | hypothetical protein | 4.75E-02 | 0.77 | 2.54 |
| BofuT4_uP146750.1 | BC1G_05915 | predicted protein | 4.57E-02 | 0.70 | 2.54 |
| BofuT4_P122600.1 | BC1G_08577 | hypothetical protein | 5.97E-03 | 0.61 | 2.53 |
| BofuT4_P150950.1 | BC1G_13469 | similar to extracellular dioxygenase | 7.61E-03 | 0.43 | 2.52 |
| BofuT4_P153300.1 | BC1G_14910 | hypothetical protein | 4.04E-02 | 0.48 | 2.52 |
| BofuT4_uP114290.1 | BC1G_15055 | hypothetical protein | 2.90E-02 | 0.71 | 2.50 |
| BofuT4_P132230.1 | BC1G_06771 | similar to RTA1 domain protein | 4.08E-02 | 0.71 | 2.50 |
| BofuT4_P123620.1 | BC1G_14604 | hypothetical protein | 7.56E-03 | 0.57 | 2.47 |
| BofuT4_uP016670.1 | BC1G_06181 | predicted protein | 4.29E-02 | 0.51 | 2.46 |
| BofuT4_P137320.1 | BC1G_13020 | similar to glucose-methanol-choline (gmc) oxidoreductase | 3.27E-02 | 0.71 | 2.46 |
| BofuT4_uP120660.1 | BC1G_03570 | hypothetical protein | 1.55E-02 | 0.82 | 2.46 |
| BofuT4_P062970.1 | BC1G_06411 | hypothetical protein | 4.81E-02 | 0.57 | 2.44 |
| BofuT4_P008800.1 | BC1G_03916 | hypothetical protein | 7.32E-03 | 0.51 | 2.42 |
| **BofuT4_P152970.1*** | **BC1G_08033** | **BcPG6. endopolygalacturonase 6** | 4.01E-02 | 0.73 | 2.42 |
| BofuT4_P042310.1 | BC1G_01545 | hypothetical protein | 3.19E-02 | 0.55 | 2.40 |
| BofuT4_P029970.1 | BC1G_01163 | hypothetical protein | 1.09E-02 | 0.49 | 2.40 |
| BofuT4_P072690.1 | BC1G_11414 | hypothetical protein | 1.15E-02 | 0.84 | 2.38 |
| BofuT4_P081200.1 | BC1G_04587 | hypothetical protein | 4.69E-03 | 0.57 | 2.37 |
| BofuT4_uP059990.1 | BC1G_11046 | predicted protein | 3.71E-02 | 0.65 | 2.37 |
| PD0AGA11YE23CM1 |  | unknown | 1.19E-02 | 0.69 | 2.34 |
| BofuT4_P035980.1 | BC1G_03991 | glycoside hydrolase family 53 protein | 7.12E-03 | 0.67 | 2.33 |
| BofuT4_P088490.1 | BC1G_06860 | hypothetical protein | 8.53E-03 | 0.46 | 2.33 |
| BC1G_13810.1 | BC1G_13810 | unknown | 1.58E-02 | 0.60 | 2.32 |
| BofuT4_uP125260.1 |  | hypothetical protein | 7.21E-03 | 0.56 | 2.32 |
| BofuT4_P086680.1 | BC1G_10222 | hypothetical protein | 8.59E-03 | 0.52 | 2.32 |
| BofuT4_P015980.1 | BC1G_11708 | similar to prolyl aminopeptidase | 3.11E-02 | 0.52 | 2.32 |
| BofuT4_uP044590.1 | BC1G_13280 | hypothetical protein | 1.14E-02 | 0.46 | 2.32 |
| BofuT4_P001750.1 |  | hypothetical protein | 3.51E-02 | 0.59 | 2.31 |
| BofuT4_P097220.1 | BC1G_11766 | similar to yippee zinc-binding-like protein | 6.14E-03 | 0.57 | 2.28 |
| BofuT4_P125200.1 |  | hypothetical protein | 2.92E-02 | 0.70 | 2.28 |
| PD0AHA1YH13CM1 |  | unknown | 2.85E-02 | 0.67 | 2.28 |
| BC1G_14243.1 | BC1G_14243 | unknown | 2.46E-02 | 0.74 | 2.26 |
| BofuT4_P067620.1 | BC1G_13943 | hypothetical protein | 1.03E-02 | 0.80 | 2.25 |
| BofuT4_uP002930.1 |  | hypothetical protein | 4.18E-02 | 0.55 | 2.25 |
| BofuT4_P108290.1 | BC1G_02639 | similar to MFS sugar transporter | 2.48E-02 | 0.58 | 2.24 |
| BofuT4_P060410.1 | BC1G_11086 | glycoside hydrolase family 76 protein | 2.41E-02 | 0.52 | 2.21 |
| BofuT4_P049060.1 | BC1G_00112 | similar to phytanoyl-CoA dioxygenase family protein | 3.10E-02 | 0.45 | 2.20 |
| BofuT4_P110300.1 | BC1G_02448 | similar to transcription factor Cys6 | 3.44E-02 | 0.58 | 2.19 |
| BofuT4_P059250.1 | BC1G_08989 | glycoside hydrolase family 6 protein | 1.42E-02 | 0.72 | 2.19 |
| PD0ADA5YJ18CM1 |  | unknown | 2.75E-02 | 0.50 | 2.18 |
| BofuT4_uP025350.1 | BC1G_07662 | hypothetical protein | 1.92E-02 | 0.41 | 2.17 |
| BofuT4_P072200.1 | BC1G_11465 | similar to P-type ATPase | 3.29E-02 | 0.48 | 2.17 |
| B3BC_116_217_D06 |  | unknown | 2.20E-02 | 0.45 | 2.16 |
| BC1G_16408.1 | BC1G_16408 | unknown | 1.47E-02 | 0.63 | 2.15 |
| BofuT4_P093840.1 | BC1G_15081 | hypothetical protein | 2.02E-02 | 0.77 | 2.15 |
| B5BC_116_304_F02 |  | unknown | 9.84E-03 | 0.74 | 2.14 |
| BofuT4_P101800.1 | BC1G_03263 | similar to PAP2 domain containing protein | 8.93E-03 | 0.90 | 2.14 |
| BC1G_02089.1 | BC1G_02089 | unknown | 3.63E-02 | 0.46 | 2.14 |
| BC1G_00942.1 | BC1G_00942 | unknown | 2.45E-02 | 0.42 | 2.13 |
| BofuT4_uP119100.1 |  | predicted protein | 4.05E-02 | 0.79 | 2.13 |
| BofuT4_P145410.1 |  | hypothetical protein | 7.52E-03 | 0.47 | 2.13 |
| BofuT4_P068640.1 | BC1G_05327 | similar to pyruvate carboxylase | 3.87E-02 | 0.47 | 2.13 |
| BofuT4_P116280.1 | BC1G_09791 | similar to prolyl aminopeptidase (secreted protein) | 4.13E-02 | 0.55 | 2.13 |
| SCL_bt4ctg_1333_bt4ctg_1334.Contig1_v5 | | unknown | 2.04E-02 | 0.68 | 2.11 |
| BofuT4_P122800.1 | BC1G_08593 | hypothetical protein | 9.17E-03 | 0.49 | 2.11 |
| BofuT4_P086000.1 | BC1G_15480 | hypothetical protein | 3.13E-02 | 0.47 | 2.11 |
| PD0ABA38YH06FM1 |  | unknown | 2.28E-02 | 0.45 | 2.10 |
| AL113735 |  | unknown | 2.42E-02 | 0.58 | 2.09 |
| BC1G_07187.1 | BC1G_07187 | unknown | 3.10E-02 | 0.55 | 2.09 |
| BofuT4_uP036660.1 |  | hypothetical protein | 1.22E-02 | 0.53 | 2.09 |
| BC1G_14780.1 | BC1G_14780 | unknown | 3.68E-02 | 0.69 | 2.08 |
| BofuT4_P040870.1 | BC1G_01404 | hypothetical protein | 9.97E-03 | 0.49 | 2.07 |
| BofuT4_P103080.1 | BC1G_13118 | similar to thioredoxin domain-containing protein | 3.85E-02 | 0.72 | 2.07 |
| BofuT4_P052080.1 | BC1G_06546 | glycoside hydrolase family 27 protein | 2.38E-02 | 0.48 | 2.05 |
| BofuT4_P128870.1 | BC1G_15220 | hypothetical protein | 5.95E-03 | 0.62 | 2.05 |
| BofuT4_P112290.1 | BC1G_13102 | similar to alpha/beta hydrolase fold | 1.43E-02 | 0.45 | 2.04 |
| BofuT4_P110210.1 | BC1G_02456 | BOP1. G protein-coupled receptor (GPCR) : Microbial opsin | 7.82E-03 | 0.65 | 2.04 |
| BofuT4_P048790.1 | BC1G_00138 | lcc1. laccase | 2.10E-02 | 0.50 | 2.04 |
| BofuT4_P023730.1 | BC1G_14877 | hypothetical protein | 8.93E-03 | 0.52 | 2.04 |
| BofuT4_P106050.1 | BC1G_13224 | hypothetical protein | 1.87E-02 | 0.45 | 2.04 |
| BofuT4_P103700.1 | BC1G_07385 | hypothetical protein | 1.73E-02 | 0.72 | 2.03 |
| BC1G_02919.1 | BC1G_02919 | unknown | 1.08E-02 | 0.43 | 2.03 |
| PD0AGA18YH14CM1 |  | unknown | 1.75E-02 | 0.54 | 2.03 |
| BofuT4_uP150460.1 |  | hypothetical protein | 3.56E-02 | 0.40 | 2.02 |
| CL_bt4ctg_0224_006.Contig1_v5 | | unknown | 8.57E-03 | 0.44 | 2.02 |
| BofuT4_P148150.1 | BC1G_09375 | similar to allantoicase | 1.70E-02 | 0.59 | 2.01 |
| BofuT4_P063230.1 | BC1G_06431 | hypothetical protein | 1.57E-02 | 0.43 | 2.01 |
| BofuT4_P074830.1 | BC1G_08972 | similar to oxidoreductase | 4.12E-02 | 0.62 | 2.00 |

* Genes whose expression profiles were confirmed by northern blot analyses (Fig. 9).
