# Supplementary material for: Natural Variation in the VELVET Gene bcvel1 Affects Virulence and Light-Dependent Differentiation in Botrytis cinerea
Source: PLoS One. 2012 Oct 31;7(10):e47840. doi: 10.1371/journal.pone.0047840 (PMC3485325; doi:10.1371/journal.pone.0047840)
Supplement: Table S3 — Identification of a BcVEL1-dependent gene cluster. (DOCX) [file pone.0047840.s014.docx]

**Table S3. Identification of a BcVEL1-dependent gene cluster.**

| **T4 annotation** | **Protein function** | **Size** | **Domain (InterProScan)** | **SP** | **TM** | **B05.10** | ***S. sclerotiorum*** |
| --- | --- | --- | --- | --- | --- | --- | --- |
| **BofuT4_P091900.1** | similar to transcription factor Cys6 | 617 aa | Cys6 transcription factor | - | - | BC1G_13502.1 | SS1G_11949.1 |
| BofuT4_uP091910.1 | hypothetical protein | 57 aa | no hit | - | - | no gene call | no gene call |
| BofuT4_uP091920.1 | predicted protein | 54 aa | no hit | - | - | no gene call | no gene call |
| **BofuT4_P091930.1** | similar to MFS multidrug transporter | 597 aa | major facilitator Superfamily | - | 12 TMs | BC1G_13501.1 | SS1G_11948.1 |
| BofuT4_uP091940.1 | predicted protein | 65 aa | no hit | - | - | no gene call | no gene call |
| BofuT4_uP091950.1 | predicted protein | 82 aa | no hit | - | - | BC1G_13499.1 | no gene call |
| BofuT4_P091960.1 | predicted protein | 195 aa | no hit | - | - | BC1G_13498.1 | no gene call |
| **BofuT4_P091970.1** | similar to bacterial integral membrane protein-like protein | 431 aa | ubiquitin family | - | - | BC1G_13497.1 | SS1G_11945.1 |
| BofuT4_uP091980.1 | predicted protein | 68 aa | no hit | - | - | no gene call | SS1G_11945.1 |
| **BofuT4_P091990.1** | hypothetical protein | 201 aa | no hit | 1 to 19 | - | no gene call | - |
| BofuT4_uP092000.1 | hypothetical protein | 58 aa | no hit | 1 to 25 | - | no gene call | - |
| **BofuT4_P092010.1** | similar to oxidoreductase | 254 aa | oxidoreductase activity | 1 to 21 | 5 to 28 | BC1G_10345.1 | - |
| **BofuT4_P092020.1** | hypothetical protein | 220 aa | DUF3328 (PF11807) | - | 41 to 61 | BC1G_10344.1 | - |
| **BofuT4_P092030.1** | hypothetical protein | 239 aa | no hit | 1 to 58 | 39 to 59 | BC1G_10343.1 | - |
| **BofuT4_P092040.1** | hypothetical protein | 184 aa | no hit | - | 44 to 64 | BC1G_10342.1 | - |
| **BofuT4_uP092050.1** | hypothetical protein | 62 aa | no hit | 1 to 22 | - | no gene call | - |
| **BofuT4_P092060.1** | glycoside hydrolase family 26 protein | 471 aa | carbohydrate metabolic process | 1 to 24 | - | BC1G_10341.1 | - |
| BofuT4_P092070.1 | predicted protein | 142 aa | no hit | - | - | no gene call | - |
| **BofuT4_P092080.1** | similar to transcription factor Zn, C2H2 | 509 aa | C2H2 transcription factor | - | - | BC1G_10340.1 | SS1G_11942.1 |
| BofuT4_P092090.1 | hypothetical protein | 159 aa | no hit | - | - | BC1G_10340.1 | no gene call |
| BofuT4_P092100.1 | hypothetical protein | 61 aa | no hit | 1 to 24 | - | no gene call | no gene call |
| **BofuT4_P092110.1** | similar to CBS and PB1 domain-containing protein | 716 aa | no hit | - | 691 to 711 | BC1G_10339.1 | SS1G_11941.1 |
| **BofuT4_P092120.1** | hypothetical protein | 365 aa | ribosomal protein S2 | - | - | BC1G_10338.1 | SS1G_11941.0 |

SP, signal peptide; TM, transmembrane domain
