# Supplementary material for: Natural Variation in the VELVET Gene bcvel1 Affects Virulence and Light-Dependent Differentiation in Botrytis cinerea
Source: PLoS One. 2012 Oct 31;7(10):e47840. doi: 10.1371/journal.pone.0047840 (PMC3485325; doi:10.1371/journal.pone.0047840)
Supplement: Table S4 — Primers used to amplify the microsatellites markers on strains T4, 32 and their progeny. (DOCX) [file pone.0047840.s015.docx]

**Table S4.** Primers used to amplify the microsatellites markers on strains T4, 32 and their progeny.

| **Microsatellite position on T4 supercontig** | | | | **Sequence (5’ ⭢ 3’)** | |
| --- | --- | --- | --- | --- | --- |
| **Name** | **Length** | **Start** | **End** | **Forward primer** | **Reverse primer** |
| **Bc389** | 446 | 2339 | 2784 | CATCTCTCCAGCTTCCTTGG | AGTATGTAAGTAGCACACGAGCAC |
| **Bc390** | 363 | 56309 | 56671 | CGATCCGGAGGAGGAACTAT | ATGGGTGAGTGGTTGGGTTA |
| **Bc246** | 163 | 97136 | 97298 | AGCGGGAAACATCAGAAAGA | GGAAAGGTACGCGTGATGAT |
| **Bc413** | 181 | 212150 | 212330 | GAATGCAATTGGATGTCTTTGA | GGGTGGAACGAGAGTGAAGA |
| **Bc427** | 165 | 212465 | 212629 | ACAGACCAAGCTCCACCATC | TGTTGCCCAACAAACAAAGA |
| **Bc433** | 168 | 250615 | 250782 | TGGAATCTCGAGTGCTGTGT | TCAATTTCCCCTTCGTCATC |
| **Bc430** | 230 | 274100 | 274329 | TCGAGACCTCTCTGCTGTCA | ACTTCTTTTGTGCGGAGGAA |
| **Bc420** | 182 | 498134 | 498315 | CCTTCGACTTGGTCGTTGTT | CTCCCTCTGAAACCCCCTAC |
| **Bc423** | 250 | 593521 | 593770 | TTTCTCTATTTTATTGAAACTGGATTT | GGAGAGTATAGTGGGAGGGTATATAAG |
