# Supplementary material for: Natural Variation in the VELVET Gene bcvel1 Affects Virulence and Light-Dependent Differentiation in Botrytis cinerea
Source: PLoS One. 2012 Oct 31;7(10):e47840. doi: 10.1371/journal.pone.0047840 (PMC3485325; doi:10.1371/journal.pone.0047840)
Supplement: Table S5 — Primers used for sequencing and mutant construction. (DOCX) [file pone.0047840.s016.docx]

**Table S5.** Primers used for sequencing and mutant construction.

| **Primer** | **Sequence (5’ ⭢ 3’)** | **Used for** |
| --- | --- | --- |
| ***bcvel1*-F1** | CACCACTCGACACCACCCACTC | Sequencing |
| ***bcvel1*-F2** | GGTCTCGAGCCGGCCACTGGAAG | Sequencing |
| ***bcvel1*-F3** | GGATCCACAACAGAGGCGTGG | Sequencing |
| ***bcvel2*-F1** | CTAGGCTGTCATTGACGACGGC | Sequencing |
| ***bcvel2*-R1** | GACCAGACGAAGGCCAGTCTTC | Sequencing |
| ***bcvel3*-F1** | GCTATGCGAGAGTCAACATGGGC | Sequencing |
| ***bcvel3*-R1** | CGATACAACGCATGTTTCGCCCG | Sequencing |
| ***bcvel4*-F1** | GCACCTCTTCCACATCTCTACTG | Sequencing |
| ***bcvel4*-R1** | CATACCATTGTCCAAATCTCTTCAC | Sequencing |
| ***bcvel1*-5F** | GTAACGCCAGGGTTTTCCCAGTCACGACGGTGCCATGTGTGTTGCTGAC | Gene replacement fragment |
| ***bcvel1*-5R** | ATCCACTTAACGTTACTGAAATCTCCAACCAGCCATTGGTGCCGACTCTC | Gene replacement fragment |
| ***bcvel1*-3F** | CTCCTTCAATATCATCTTCTGTCTCCGACCAACCATGTCGTCAGTCACG | Gene replacement fragment |
| ***bcvel1*-3R** | GCGGATAACAATTTCACACAGGAAACAGCACCTTCTACCTCCCATCTAC | Gene replacement fragment |
| ***hph*-F** | GTCGGAGACAGAAGATGATATTGAAGGAGC | Gene replacement fragment |
| ***hph*-R** | GTTGGAGATTTCAGTAACGTTAAGTGGAT | Gene replacement fragment |
| ***bcvel1*-hi5F** | GAGCGCCATTGGTAGGTAGTCCG | Diagnostic PCR |
| **pCSN44-trpC-T** | GGAATAGAGTAGATGCCGACCGG | Diagnostic PCR |
| ***bcvel1*-hi3R** | GGCTATTAGCTATTAGCTATTAGTCG | Diagnostic PCR |
| **pCSN44-trpC-P** | CCTCCACTAGCTCCAGCCAAGCCC | Diagnostic PCR |
| ***bcvel1*-WT-F** | GGGCATGCCAGTGTCTGGAATGG | Diagnostic PCR |
| ***bcvel1*-WT-R** | CTAGTTGAGTACGCAGGAGGATTGC | Diagnostic PCR |
| ***bcvel1*-COM-F** | GACTGCCCGGGTCACGCTAAAAG | Complementation construct A |
| ***bcvel1*-COM-R** | GCATAAACAACACCCCCGGGAGTCGTATAG | Complementation construct A |
| ***bcvel1*-COM-5R** | ATCCACTTAACGTTACTGAAATCTCCAACGGTATTCAACTCTGTGCAGTCC | Complementation construct B |
| ***bcvel1*-COM-3F** | CTCCTTCAATATCATCTTCTGTCTCCGACCCTACCTATTTACATGAGATGAA | Complementation construct B |
| ***hph-*R-trpC-T2** | TGGAGATTTCAGTAACGTTAAGTGGATCGTATCTTATCGAGATCCTGAACA | Complementation construct B |
| ***nat1-R1*** | CAGTGCCTCGATGGCCTCGGCGTC | Diagnostic PCR |
| ***bcvel1*-GFP-F** | CTTACCTCACCCTTGGAAACCATGTTAAGACCGGGGGCGGGTTTAT | GFP fusion construct |
| **B05.10-GFP-R** | CCATCACATCACAATCGATCCAAATGGCTGCGTCCATAGGTCCCAAAA | GFP fusion construct |
| **T4-GFP-R** | CTTACCTCACCCTTGGAAACCATATCAAAAGACGATTCGGCAGCGGC | GFP fusion construct |
| ***bcniiA*-hi5F** | GCGGGGTATGGCAGCATGAGTG | Diagnostic PCR |
| **T*gluc*-hiF** | CATACGTACATCTGATTTGACAACC | Diagnostic PCR |
| ***bcniiA*-hi3R** | CTTATAGCAAGCGCGATGTGTATC | Diagnostic PCR |
| ***nat1*-hiF** | CGGCGAGCAGGCGCTCTACATGAGC | Diagnostic PCR |
| ***bcniiA*-WT-F** | GGTTGAGGTGGTGGAAGATTTG | Diagnostic PCR |
| ***bcniiA*-WT-R** | CGACCACCAAGCCTCCAGCATC | Diagnostic PCR |
